# Multi-residue ultra-performance liquid chromatography coupled with tandem mass spectrometry method for comprehensive multi-class anthropogenic compounds of emerging concern analysis in a catchment-based exposure-driven study

**DOI:** 10.1007/s00216-019-02091-8

**Published:** 2019-09-07

**Authors:** Kathryn Proctor, Bruce Petrie, Ruth Barden, Tom Arnot, Barbara Kasprzyk-Hordern

**Affiliations:** 1 Department of Chemistry, University of Bath, Claverton Down, Bath, BA2 7AY UK; 2 Water Innovation & Research Centre, Department of Chemical Engineering, University of Bath, Claverton Down, Bath, BA2 7AY UK; 3 School of Pharmacy and Life Sciences, Robert Gordon University, Aberdeen, AB10 7JG UK; 4 Wessex Water Services Ltd., Claverton Down, Bath, BA2 7WW UK

**Keywords:** Chemicals of emerging concern, Analysis, Environment, Mass spectrometry

## Abstract

This paper presents a new multi-residue method for the quantification of more than 142 anthropogenic compounds of emerging concern (CECs) in various environmental matrices. These CECs are from a wide range of major classes including pharmaceuticals, household, industrial and agricultural. This method utilises ultra-performance liquid chromatography coupled with tandem mass spectrometry (UPLC-MS/MS) for analysis of five matrices (three liquid and two solid) from wastewater treatment processes and the surrounding environment. Relative recoveries were predominantly between 80 and 120%; however, due to the complexity of the matrices used in this work, not all compounds were recovered in all matrices, from 138/142 analytes in surface water to 96/142 analytes in digested solids. Method quantification limits (MQLs) ranged from 0.004 ng L^−1^ (bisoprolol in surface water) to 3118 ng L^−1^ (creatinine in wastewater treatment work (WwTW) influent). The overall method accuracy was 107.0%, and precision was 13.4%. To test its performance, the method was applied to the range of environmental matrices at WwTWs in South West England. Overall, this method was found to be suitable for application in catchment-based exposure-driven studies, as, of the total number of analytes quantifiable in each matrix, 61% on average was found to be above their corresponding MQL. The results confirm the need for analysing both the liquid and solid compartments within a WwTW to prevent under-reporting of concentrations.

**Electronic supplementary material:**

The online version of this article (10.1007/s00216-019-02091-8) contains supplementary material, which is available to authorized users.

## Introduction

The use of anthropogenic, household, industrial or agricultural chemicals such as pharmaceuticals, pesticides, plasticisers, UV filters, industrial chemicals and microplastics is ubiquitous, and they have been recognised as a source of environmental contamination. These compounds have been quantified at levels ranging from ng L^−1^ to μg L^−1^, and their impact on the environment is not well known. These compounds are often designed to be biologically active and can be persistent in the environment, where they have the potential to bioaccumulate within the tissues of organisms [1–4]. For these reasons, among many others, they are known as compounds of emerging concern (CECs) [5–7].

CECs are primarily introduced to the environment via point sources such as wastewater treatment works (WwTWs), industrial discharge points and landfill leachates [8–11]. Diffuse sources, such as direct application to land in agriculture, have been identified as a source of select sub-classes of CECs, such as pesticides and veterinary pharmaceuticals. Additionally, the application of digested sludge, from WwTW processes, directly to the land in modern farming practices is a potential source of other classes of CECs [12]. Bisphenol A (BPA) for instance, has been found at high levels within this matrix [13, 14].

CECs have been detected across the world in a multitude of environmental matrices [6, 15, 16]. This is due to their widespread use and to partitioning that can occur from the aqueous phases into suspended solids and sediments, where it can affect terrestrial organisms and fauna. Whilst their presence does not necessarily mean harm, the ecotoxicological effects of many of the CECs have been quantified in laboratory-based studies for a variety of different organisms across trophic levels and toxic effects have been demonstrated [17–19]. These laboratory-based ecotoxicological studies broadly focus on a single compound versus a single organism, but the environment is a ‘cocktail’ of CECs and different microorganisms. Ermler et al. [20] addressed this lack of knowledge for anti-androgens and found that concentration addition is a good model for predicting the effect of mixtures for up to six compounds. Leading on from this work, Orton et al. [21] tested multi-component mixtures of up to 30 compounds with varying mixture ratios. At the point where one mixture caused a 10% inhibition of the cancer cell assay, the concentrations of the components were a factor of 5.8 lower than the concentration that would be needed for them to individually cause this effect [21]. This highlights the need for current analytical methods to have method quantification limits (MQLs) lower than the no-observed effect concentrations (NOECs) of individual CECs, to enable accurate measurements of very low concentrations, for a better understanding of the risk they pose to the environment in combination.

Work is being done to further understand the fate, behaviour and effect of CECs within our environment. However, the sheer number of them and their everchanging usage makes this a challenge. The European Inventory of Existing Commercial Chemical Substances (EINECS) contains over 100,000 substances [22], with further substances being registered across all EEA countries, through Registration, Evaluation, Authorisation and Restriction of Chemicals (REACH). Currently, there are 94,705 registrations containing 22,257 substances, or 22,096 unique substances that are in use [23]. This number is increasing every year, as new substances are developed and registered, and this pattern can be found across all classes of CECs.

The exponential growth of populations across the world, due to increased life expectancy and decreased infant mortality, is increasing the usage of pharmaceuticals. This puts further pressure on agriculture to produce food faster and cheaper, often via the use of pesticides, herbicides and other anthropogenic compounds. With the number of CECs in use, and more being developed each year, it is not feasible to determine the exposure and effects of all these compounds in a single catchment, let alone across a country or continent. To further complicate matters, many CECs degrade through different processes such as hydrolysis, photolysis and metabolism. These form transformation products and metabolites, which are often more harmful than the parent compounds [24].

Brack et al. [25] conducted a review of the EU Water Framework Directive (WFD) and concluded that there are specific challenges both at European scale and at a local scale. Regulation and national monitoring schemes such as the WFD and the UK Chemical Investigations Programme (CIP) are suitable for furthering the understanding of this problem on a wider scale. Through identification and assessment of the most widespread CECs, high-risk compounds can be identified and managed through harmonised methods across diverse areas. However, they are often limited by the sampling method, i.e. a few samples collected at many locations, using only grab samples, which are not very representative [26]. Sampling at a local scale is crucial to determine catchment-specific substances and mixtures that might be a specific problem in the local environment. This can allow more targeted management of risks and hazards at local catchment level. There is a further need for analytical methods which can detect CECs down to, or below, the predicted no-effect concentration (PNEC), as this will allow a more adequate risk assessment [27].

To gain a better understanding of the fate of CECs within an environmental catchment, analysis of the influent and effluent of contributing WwTWs is required, including analysis of solid particulate matter (SPM) and digested solids, as well as corresponding surface water. Overall, in the literature, there is still a lack of multi-residue methods for quantification of CECs in solid matrices. Even fewer publications consider both the liquid and solid phases in the WwTW or the environment. Many studies focus specifically on pharmaceuticals, personal care products, industrial chemicals, veterinary pharmaceuticals or pesticides. However, a catchment potentially contains compounds from a variety, if not all, classes of CECs, though it is rare that they are all the focus of analysis in a single campaign. One study by Gustavsson et al. [28] covers many different classes of CEC, leading to the analysis of 172 compounds; however, this was only achieved through multiple sample preparation methods. For a comprehensive understanding of the exposure and environmental risk of chemical mixtures, multi-residue quantitative methods covering a large variety of CECs are required.

The aim of this work is to develop and validate a new multi-residue method (< 190 compounds including internal standards) for a wide range of CECs prioritised for risk assessment at a catchment scale, and accounting for the highly urbanised and agricultural areas of one catchment. The classes of CECs covered by this method include the following: UV filters, parabens, plasticisers, steroid estrogens, antibacterials/antibiotics, antifungals, hypertension drugs, non-steroidal anti-inflammatory drugs (NSAIDs), lipid regulators, antihyperlipidaemics, antihypertensives, antihistamines, drugs for erectile dysfunction, drugs for diabetes, cough suppressants, beta blockers, H_2_ receptor agonists, X-ray contrast media, drug precursors, anticancer drugs, anaesthetics, antidepressants, anti-epileptics, calcium channel blockers, hypnotics, antipsychotics, drugs for dementia, human indicators, analgesics, stimulants, opioids, drugs used in veterinary medicine, pesticides, fungicides and herbicides and metabolites. The selection of analyte groups was based not only on prioritisation including existing and proposed legislation, European and national watch lists (UKWIR CIP, EU Watch List) [29–32] and the literature [33, 34] but also on exploring usage statistics (NHS prescriptions [35]), entry into the environment (metabolism, excretion from DrugBank [36]), persistence, bioaccumulation, transport throughout the environment and toxicity of organisms [mammals, aquatic and benthic (log *K*_ow_, log *K*_oc_, log *D*_ow_, water solubility, vapour pressure, Henry’s law constant, bioconcentration factor, EPI Suite, ACD/Labs [37, 38])].

## Materials and methods

This paper provides an expanded and broader scope method based on the method published by Petrie et al. [39]. Electronic Supplementary Material (ESM) Table S1 contains data on the suppliers of the compounds as well as their physiochemical properties.

The analytes were primarily purchased in solid form, before being accurately weighed and dissolved in HPLC grade methanol (MeOH) (Sigma-Aldrich), or other suitable solvents, at a concentration of 1.0 mg mL^−1^. These stock solutions were stored in silanised glass vials in the dark at − 20 °C, unless otherwise stated. Multi-analyte mixtures were prepared from these stock solutions. The CECs, their corresponding internal standards, data acquisition method and MS/MS detection parameters can be found in ESM Table S2. Chromatograms of all analytes can be found in ESM Fig. S1.

The internal standards 1*S*,2*R*-(+) ephedrine-d3, amphetamine-d5, benzoylecgonine-d8, cocaethylene-d8, cocaine-d3, codeine-d6, cotinine-d3, 2-ethylidene-1,5-dimethyl-3,3-diphenylpyrroloidine-d3 (EDDP-d3), estradiol (2,4,16,16-d4), estrone (2,4,16,16-d4), heroin-d9, ketamine-d4, 3,4-methylenedioxyamphetamine-d5 (MDA-d5), 3,4-methylenedioxymethamphetamine (MDMA-d5), mephedrone-d3, methadone-d9, methamphetamine-d5, methylparaben-13C, morphine-d3, norketamine-d4, quetiapine-d8 and tempazepam-d5 were purchased from LGC Standards (Middlesex, UK). Amitrpityline-d3, amoxicillin-d4, capecitabine-d11, ciprofloxacin-d8, citalopram-d6, diazepam-d5, erythromycin-13C,d3, fluoxetine-d5, gabapentin-d4, imidacloprid-d4, metazachlor-d6, metoprolol-d7, mirtazapine-d3, norsertraline-d4, nortriptyline-d3, ofloxacin-d3, oxazepam-d5, sildenafil-d8 and verapamil-d7 were purchased from Toronto Research Chemicals (TRC) (Toronto, Canada). Acetaminophen-d4, atenolol-d5, bisphenol A-d16, carbamazepine-13C6, ibuprofen-d3, ketoprofen-d3, metformin (dimethyl-d6), methiocarb-d3, naproxen-d3, propranolol-d7, sertraline-d3 and tamoxifen-13C2,15N were purchased from Sigma-Aldrich (Gillingham, UK), and bezafibrate-d6 was purchased from QMX Laboratories (Thaxted, UK). These were purchased as solutions at a concentration of 0.1 mg mL^−1^ or 1.0 mg mL^−1^ in methanol or other appropriate solvents. If no solutions were available, 1.0 mg powder was purchased, and the entire contents of the vial were dissolved in methanol. The MS/MS detection parameters for the internal standards can be found in ESM Table S3.

All glassware in this paper was silanised to prevent the analytes and internal standards from absorbing to the surface. This was done by coating the internal surfaces of the glassware with 5% dimethylchlorosilane (DMDCS) in toluene (Sigma-Aldrich), rinsing with toluene (Sigma-Aldrich) twice, then rinsing again with MeOH three times, and leaving to dry between each coating or rinse.

### Methods for sample collection

Sampling was carried out in a river catchment in South West England. Sampling involved collection of samples from all major WwTWs and receiving environmental waters. Twenty-four-hour composites using a 3700 ISCO sampler (RS Hydro) were collected for both influent and effluent wastewaters in each case. Grab samples were utilised in surface water samples. SPM was collected from influent samples.

SPM per litre was calculated by filtering 30 mL through a pre-dried and pre-weighed GF/F glass microfibre filter. This was then re-dried at 105 °C for 8 h, left to cool and re-weighed to quantify SPM in grams per litre. Data are shown in ESM Table S4. Digested sludge was collected on three consecutive days, both directly after digestion and prior to disposal. Data are shown in ESM Table S5.

All samples were kept on ice during sampling (ice was placed within the composite sampler to maintain a cool temperature of 0–4 °C and promote stability) or placed in a cool box and kept on ice until the samples were transported to the lab. Once at the lab, samples for liquid analysis were transferred to 125-mL PPE bottles (Fisherbrand) and frozen (− 20 °C) for further preparation and analysis at a later date. For the influent samples, the remainder of the sample was filtered to collect the SPM, which was then frozen (− 20 °C). Most compounds do not adsorb to the PPE bottles, with very few exceptions (ESM Table S6).

### Methods for extraction and analysis

**Fig. 1:**
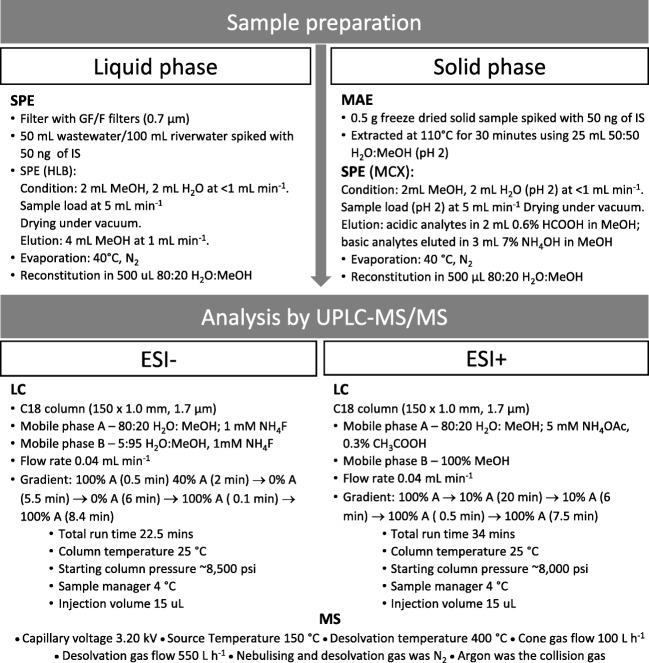
Flow chart from sample preparation to analysis, for analysis of liquid and solid samples by ESI− and ESI+ methods

The methods used for sample preparation of both liquid and solid matrices, as well as their analysis, can be found in Fig. 1; this is also discussed in more detail below. Development of an extraction method for liquid matrices with hydrophilic-lipophilic-balanced solid-phase extraction (HLB SPE) was developed based on the method published by Kasprzyk-Hordern et al. [40]. The microwave-assisted extraction (MAE) for solid matrices was developed based on the method published by Petrie et al. [39].

### Extraction for liquid matrices

The samples were filtered with a GF/F glass fibre filter (0.7 μm) (Whatman, Sigma-Aldrich) and adjusted to pH 7.5–8.5. 50 mL of influent or effluent (100 mL surface water) was accurately measured and spiked with 50 μL of 1 μg mL^−1^ (50 ng) internal standard mixture in MeOH. The 60-mg HLB SPE cartridges (OASIS, Waters, UK) were conditioned and equilibrated with 2 mL of MeOH, followed by 2 mL of deionised water (H_2_O) at a rate of < 1 mL min^−1^ (under gravity). The deionised H_2_O was obtained from a Milli-Q system (18.2 MΩ). The sample was loaded at 5 mL min^−1^ before the cartridges were dried under vacuum. Analytes were eluted using 4 mL MeOH at 1 mL min^−1^ (under gravity). Once eluted, the extracts were evaporated to dryness at 40 °C, with a steady flow of nitrogen using a TurboVap LV concentration workstation. Finally, the samples were reconstituted in 500 μL of 80:20 H_2_O:MeOH, mixed thoroughly to ensure completed dissolution and transferred to LC vials (polypropylene) (Waters, UK).

### Extraction for solid matrices

The solid samples, digested sludge and SPM were initially frozen and freeze-dried (ScanVac, CoolSafe freeze dryer, Lynge, Denmark). The freeze-dried samples were homogenised, and 0.25–0.5 g was weighed out and spiked with 50 μL of 1 μg mL^−1^ (50 ng) internal standard mixture in MeOH. This was left for 30 min to 1 h, for the methanol to evaporate off. The samples were then transferred to MAE perfluoroalkoxy (PFA) tubes with 25–30 mL of 50:50 acidified H_2_O (pH 2):MeOH. The MAE tubes were placed in 800 W MARS 6 microwave (CEM, UK). The temperature was ramped to 110 °C, over 10 min, then held at this temperature for 30 min, before allowing the samples to cool. SPE was then carried out using 60-mg mixed-mode cationic exchange (MCX) cartridges (Oasis, Water, UK). These were conditioned and equilibrated using 2 mL MeOH and 2 mL acidified H_2_O (pH 2) at < 1 mL min^−1^ under gravity. The samples were then loaded at 5 mL min^−1^ and dried under vacuum. Once dried, the acid analytes were eluted first with 2 mL of 0.6% formic acid (HCOOH) (Sigma-Aldrich) in MeOH. The basic analytes were eluted second with 3 mL of 7% ammonium hydroxide (NH_4_OH) in MeOH (Sigma-Aldrich). These extracts were evaporated to dryness at 40 °C, with a steady flow of nitrogen using a TurboVap. The residue was reconstituted in 500 μL of 80:20 H_2_O:MeOH, mixed thoroughly to ensure completed dissolution and transferred to LC vials.

### Analysis of samples

The analytes were separated by UPLC performed on the Waters ACQUITY UPLC™ system (Waters, UK). The column used was a reversed-phase C18 column (Waters, UK), 150 mm × 1.0 mm, with a particle size of 1.7 μm. The samples were analysed with Xevo Triple Quadrupole (TQD) Mass Spectrometer (Waters, UK), equipped with an electrospray ionisation (ESI) source in positive and negative modes. To optimise ionisation, two different sets of parameters were used for the ESI-positive and ESI-negative modes. The parameters for these can be seen in Fig. 1. The systems were controlled using MassLynx (Waters, UK). Argon (99.998%) gas, supplied by a BOC cylinder, was used as a collision gas. The nebulising gas was nitrogen, provided by a high-purity nitrogen generator (Waters, Manchester, UK). Two mobile phases were used in the gradient mode ESI+: mobile phase A contained 80:20 H_2_O:MeOH with 5 mM ammonium acetate (NH_4_OAc) and 3 mM acetic acid (CH_3_COOH) (pH 4.7), and mobile phase B contained 100% MeOH. Starting conditions were 100% A, decreasing to 10% A over 20 min, maintained at this level for 6 min, before increasing back to 100% A over 0.5 min and held for 7.5 min to return the column to equilibrium; in ESI−: mobile phase A contained 80:20 H_2_O:MeOH with 1 mM ammonium fluoride (NH_4_F) and mobile phase B, which was 5:95 H_2_O:MeOH containing 1 mM NH_4_F. The gradient began at 100% A for 0.5 min and reduced to 40% A over 2 min, before being further reduced to 0% A over the next 5.5 min. It was held at 0% A for 6 min before increasing back up to 100% A over 0.1 min. This was maintained for 8.4 min to re-equilibrate the column. The HPLC grade MeOH, NH_4_OAc (Fluka) and CH_3_COOH were obtained from Sigma-Aldrich (Gillingham, UK). The NH_4_F (Fluka) was obtained from Fisher Scientific (Loughborough, UK) and deionised water (18.2 MΩ) obtained from a Milli-Q system. The temperature of the built-in sample manager was 4.0 °C with an injection volume of 20 μL. The mobile phases were run at a rate of 0.04 mL min^−1^, as a gradient of a high ratio of aqueous (80%) to 100% MeOH in both positive and negative ionisation modes. The exact gradients and composition of the mobile phases can be also seen in Fig. 1. Data processing was carried out using TargetLynx software, which is an extension to MassLynx (version 4.1, Waters).

### Instrument performance

To quantify the analytes, an internal standard approach with an 18-point calibration curve was used. For the majority of the compounds, the analysed range covers 6 orders of magnitude, from ng L^−1^ to mg L^−1^. Each point was repeated every 24 h over 3 days. These calibration samples were prepared in a ratio of 80:20 H_2_O:MeOH unbuffered solutions. The signal-to-noise ratios of these samples were used to determine the concentration of the instrument detection limit (IDL) and instrument quantification limit (IQL), where S/N ≥ 3 or S/N ≥ 10, respectively. Determination coefficients (*r*^2^) were calculated for the full linear range (IDL to ≤ 1000 μg L^−1^). Inter- and intra-day precision and accuracy were calculated from repeated injections, at regular intervals (*n* = 3) of three concentrations (10 μg L^−1^, 100 μg L^−1^, 500 μg L^−1^) in 80:20 H_2_O:MeOH, across 24 h (intra-day) and across 72 h (inter-day).

### Method performance

As SPE was used as a preconcentration and clean-up step, the recovery of each analyte must be assessed. Absolute and relative recoveries for SPE of liquid matrices were calculated from matrices spiked in duplicate (*n* = 2) at three concentrations for A-ESI+ (100 ng L^−1^, 1000 ng L^−1^ and 5000 ng L^−1^ for effluent and influent, and 50 ng L^−1^, 500 ng L^−1^ and 2500 ng L^−1^ for surface water). For B-ESI+ and C-ESI−, the matrices were spiked at two concentrations (100 ng L^−1^ and 1000 ng L^−1^ for effluent and influent, and 50 ng L^−1^ and 500 ng L^−1^ for surface water). For influent suspended particulate matter (SPM) and digested sludge (DS), the absolute and relative recoveries take into account MAE and SPE. The samples were spiked at 50 ng g^−1^ and 100 ng g^−1^.

Method detection limits (MDLs) and MQLs were calculated using Eq. 1

1 $$ \mathrm{MDL}=\frac{\mathrm{IDL}\times 100}{\mathrm{Rec}\times \mathrm{Cf}} $$

where IDL is the instrumental detection limit, which is calculated as discussed in the section “Method performance”; 100 is the conversion factor for recovery of a specific matrix (Rec); and Cf is the concentration factor for the specific liquid matrix, e.g. 200 for surface waters or 100 for effluent of effluent. For solid matrices, Cf is replaced with a conversion factor of 2, which converts the volume into grams, based on the 0.25 g of solid matrix being extracted into a 0.5-mL vial for analysis. MQL is calculated with the same equation but by replacing IDL with IQL.

Furthermore, the accuracy and precision of the overall method, including SPE, are also required. These were calculated from samples of 3 matrices taken from 5 different WwTWs in the South West UK. These samples were spiked at 100 ng L^−1^ and 1000 ng L^−1^ for influent and effluent samples and at 50 ng L^−1^ and 500 ng L^−1^ for surface water for A-ESI+ and at 100 ng L^−1^ for all matrices for B-ESI+ and C-ESI−. The accuracy of the method was determined from the percentage deviation from the known concentration of analyte added to the sample. Precision was calculated as the relative standard deviation (RSD) of the replicates.

It has been found that complex matrices such as influent can affect the detection of analytes, especially when these samples have undergone SPE with HLB cartridges, as these cartridges extract a huge range of compounds. Therefore, matrix suppression was determined for the liquid matrices, which were extracted by employing a method using this approach. Samples for calculating matrix suppression were prepared by spiking samples with 50 μL of 1 μg mL^−1^ of internal standards, after the elution step of SPE, and prior to evaporation and reconstitution. Once analysed, matrix suppression for each analyte was calculated using the following equation:

2 $$ \mathrm{Matrix}\ \mathrm{suppression}=1-\left(\frac{\mathrm{PA}\ \mathrm{in}\ \mathrm{SS}-\mathrm{PA}\ \mathrm{in}\ \mathrm{US}}{\mathrm{PA}\ \mathrm{in}\ \mathrm{MP}\ \mathrm{QC}}\right) $$

where PA is the peak area of the analyte in spiked sample (SS), unspiked sample (US) and mobile phase quality control (MP QC) sample.

All matrices used were collected via grab sampling and homogenised, and all analyses were carried out on this single sample to ensure consistent results. Due to the use of these environmental matrices and the potential presence of analytes within the matrices prior to spiking, the ‘blank’ or unspiked (with analytes) portion of the sample was spiked with internal standards and analysed to confirm the concentration of analytes, prior to spiking, for recoveries and matrix effect analysis.

### Quality control

Quality control samples were analysed before and after each batch at three concentrations (10 μg L^−1^, 100 μg L^−1^, 500 μg L^−1^) along with procedural blanks, to ensure the method and instrumental performance and to monitor for contamination of the equipment.

All samples were spiked with the internal standards listed in ESM Table S3, for accurate quantification, and to account for loss from the point of spiking until analysis and to limit matrix effects.

## Results and discussion

This paper provides an expanded and broader scope method based on a method published by Petrie et al. [39], enabling the analysis of household and agricultural chemicals whilst utilising one sample preparation protocol and comprehensive UPLC-MS/MS methodology.

### UPLC-MS/MS method

All analytes were analysed using MRM and ESI− and ESI+ modes and conditions previously selected by Petrie et al. [39]. Following EU guidelines, two MRM transitions were used for most of the 195 compounds (142 analytes and 53 ISTDs). This is except for cefalexin, ketoprofen, diclofenac, ibuprofen, 1,7-dimethylxanthine and norfluoxetine, which are to be considered as semi-quantitative. For ESI−, the parent ion [M−H]^−^ was selected, and for ESI+ mode, [M−H]^+^ was selected. The most abundant daughter ion was used for quantification and the second most for confirmation. ESM Table S2 includes the MRMs for all analytes, the acquisition method was used to analyse them and the assigned internal standard. The MRMs for the internal standards can be found in ESM Table S3.

The reversed-phase BEH C18 column provided good separation and sensitivity for all compounds. These conditions achieved good separation and peak shape for most analytes. Further information can be found in Fig. 1. A more acidic mobile phase was also trialled in the ESI+ method, and although the peak shape and separation for the acidic compounds, particularly the fluoroquinolones, were improved slightly, they only benefitted a small number of compounds and had a detrimental effect against many others. Satisfactory separation of analytes was achieved within a < 40-min retention time.

Quality control criteria as recommended by the EU directive [41] utilised quality control samples, standard tolerances of ion ratio, chromatographic retention time, relative retention time and signal-to-noise ratio.

### Instrument performance

The instrument performance was assessed by considering linear response, inter- and intra-day precision and accuracy and, finally, instrumental detection and quantification limits. This data can be found in Table 1.

**Table 1:** Instrumental performance data for ECs of interest in the mobile phase (ordered by class)

Class of analyte	Analyte	RT	RRT	Linearity		Intra-day instrumental performance		Inter-day instrumental performance		IDL_S/N_ (μg L^−1^)	IQL_S/N_ (μg L^−1^)
				Range (μg L^−1^)	*r*^2^	Precision (deviation) (%)	Accuracy (%)	Precision (%)	Accuracy (%)		
UV filter	Benzophenone-1	9.6	0.9	0.06–684.0	0.996	2.3	106.8	3.3	106.7	0.01	0.06
	Benzophenone-2	7.7	1.0	0.05–583.8	0.997	1.1	99.6	4.2	97.6	0.01	0.05
	Benzophenone-3	21.2	1.2	0.05–404.0	0.995	3.2	84.9	4.5	86.8	0.01	0.05
	Benzophenone-4	6.9	0.9	1.01–502.5	0.997	2.3	103.0	3.8	105.1	0.31	1.01
Parabens	Methylparaben	7.5	1.0	0.06–1122	0.998	1.1	93.3	6.0	97.4	0.01	0.06
	Ethylparaben	8.3	1.0	0.11–663.6	0.997	2.6	112.3	2.1	113.1	0.03	0.11
	Propylparaben	9.2	1.0	0.12–462.0	0.997	5.7	96.4	4.3	98.4	0.04	0.12
	Butylparaben	10.1	1.0	0.06–696.6	0.997	5.0	97.1	3.6	100.3	0.01	0.06
Plasticizer	Bisphenol A	9.0	1.1	0.10–626.4	0.997	2.4	103.6	1.3	104.6	0.03	0.10
Steroid estrogens	E1	9.8	1.0	0.49–989.0	0.998	1.8	96.9	2.1	98.6	0.10	0.49
	E2	9.8	1.0	0.47–949.0	0.997	3.1	96.6	2.6	96.3	0.09	0.47
	EE2	9.7	1.0	0.48–950.0	0.997	2.6	94.6	3.3	93.2	0.10	0.48
Antibiotics and antibacterials	Sulfasalazine	7.1	0.8	0.90–904.0	0.999	3.9	105.2	2.4	104.7	0.27	0.90
	Clarithromycin	18.9	1.1	0.06–561.0	0.999	2.6	99.8	2.4	101.8	0.01	0.06
	Azithromycin	14.0	0.9	0.001–1000	0.998	4.5	108.9	1.5	102.0	0.01	0.05
	Trimethoprim	8.4	1.0	0.10–500.0	0.998	3.0	96.9	2.2	99.5	0.03	0.10
	Sulfamethoxazole	9.6	1.0	0.10–1000	0.999	3.5	95.1	2.4	96.0	0.03	0.10
	Triclosan^a^	12.3	1.2	1.13–225.6112.8–1128	0.997/0.998	9.4	69.1	6.5	71.4	0.34	1.13
	Amoxicillin	3.1	0.2	0.06–439.5	0.995	5.3	105.7	6.7	94.4	0.02	0.06
	Metronidazole	5.3	1.0	1.00–1000	0.999	2.5	105.0	1.2	102.9	0.06	0.21
	Sulfadiazine	4.8	0.9	0.05–795.2	0.999	2.8	105.3	1.5	104.4	0.01	0.03
	Cefalexin^b^	9.2	0.3	15.9–200	0.995	9.5	111.3	12.3	102.9	4.78	15.94
	Ofloxacin	9.6	1.0	0.23–986.0	0.998	4.2	97.4	2.8	95.9	0.07	0.23
	Ciprofloxacin	9.9	1.0	1.18–902	0.999	8.7	89.0	5.5	90.2	0.35	1.18
	Tetracycline	10.0	1.0	0.06–864.0	0.999	6.8	115.1	8.5	113.1	0.02	0.06
	Danofloxacin	10.2	1.0	1.05–1000	0.998	7.3	106.0	6.0	99.2	0.32	1.05
	Oxytetracycline	10.4	1.1	2.36–800.8	0.997	4.6	93.5	3.0	88.9	0.71	2.36
	Chloramphenicol	12.6	0.6	1.74–400	0.999	3.8	103.5	3.0	100.8	0.52	1.74
	Penicillin G	13.1	0.5	4.68–93.6	0.994	10.3	115.5	4.4	111.7	0.02	0.07
	Penicillin V	14.5	0.8	5.00–200	0.993	4.4	88.5	15.0	96.8	0.15	0.49
	Erythromycin	17.2	1.0	204.4–1022	0.999	2.3	94.4	2.9	95.2	0.20	0.65
	Prulifloxacin	18.0	1.9	100–1000	0.997	4.4	98.7	8.9	86.4	2.44	8.13
	Norfloxacin	9.7	1.0	0.01–1000	0.996	4.1	85.5	4.4	85.1	0.002	0.01
Antifungal	Griseofulvin	17.2	0.9	0.26–205.2	0.999	1.6	89.2	3.0	91.6	0.08	0.26
	Ketoconazole	21.7	1.2	0.02–800.0	0.999	3.8	94.8	2.5	91.7	0.01	0.02
Hypertension	Valsartan	7.6	0.9	1.12–1122	0.998	1.9	115.8	3.5	118.6	0.34	1.12
	Irbesartan	8.6	1.0	0.50–603.6	0.998	2.6	96.9	4.1	98.3	0.10	0.50
	Lisinopril	7.1	0.9	0.93–372.5	0.995	2.2	97.2	7.2	95.2	0.09	0.93
NSAIDs	Ketoprofen^b^	7.9	0.9	0.54–1085	0.998	2.2	99.9	2.6	99.4	0.11	0.54
	Ibuprofen^b^	9.8	1.0	0.05–1071	0.998	2.4	93.7	2.3	94.2	0.01	0.05
	Naproxen	8.1	1.0	0.49–989.0	0.998	1.5	97.7	2.5	98.3	0.10	0.49
	Diclofenac^b^	9.0	1.0	0.10–619.2	0.997	7.9	89.6	4.5	91.8	0.03	0.10
	Acetaminophen	5.1	1.0	0.54–1070	0.998	1.6	97.4	2.6	99.0	0.11	0.54
Lipid regulator	Bezafibrate	7.9	1.0	0.10–976.0	0.998	2.3	97.8	2.8	97.9	0.03	0.10
	Atorvastatin	9.3	1.1	0.05–500.0	0.997	2.6	98.0	3.5	100.9	0.01	0.05
Antihyperlipidaemic	Gemfibrozil	23.3	1.2	1.01–100.5	0.994	7.8	118.5	6.9	121.1	0.11	0.35
Antihyperintensive	Candesartan cilexetil	23.0	0.9	226.8–680.4	0.995	5.2	100.5	0.9	106.9	1.58	5.28
Antihistamine	Fexofenadine	8.4	1.0	0.09–937.5	0.998	2.1	106.3	6.5	104.6	0.03	0.09
	Cetirizine	18.7	1.0	0.08–417.7	0.999	1.3	100.5	1.3	100.8	0.02	0.08
GUD/ED	Sildenafil	18.3	1.0	0.01–1000	1.000	3.5	99.5	3.0	99.1	0.002	0.01
Diabetes	Metformin	2.8	1.0	0.43–862.5	0.998	1.5	96.3	1.3	97.0	0.09	0.43
	Gliclazide	17.8	1.0	0.05–508.0	0.997	2.1	93.2	2.8	95.3	0.01	0.05
	Sitagliptin	11.8	0.7	0.08–646.4	0.998	3.2	111.7	3.0	110.3	0.01	0.02
Cough suppressant	Pholcodine	3.7	0.9	1.14–570.0	0.999	4.7	99.5	3.3	99.2	0.35	1.14
Beta blocker	Atenolol	4.3	1.0	0.10–502.5	0.999	2.1	95.3	2.3	96.8	0.03	0.10
	Metoprolol	11.2	1.0	0.05–507.5	0.999	1.3	96.8	2.0	96.1	0.01	0.05
	Propranolol	15.1	1.0	0.09–434.9	0.999	2.0	105.4	1.0	106.2	0.03	0.09
	Bisoprolol	13.7	0.8	0.10–1004	0.999	4.8	100.4	2.0	96.0	0.0004	0.0012
H_2_ receptor agonist	Ranitidine	4.6	1.1	5.17–517.0	0.998	2.5	100.1	9.7	97.4	1.03	5.17
	Cimetidine	5.3	1.0	0.52–1043	0.999	4.2	104.1	9.0	99.3	0.10	0.52
X-ray contrast media	Iopromide	4.9	0.9	5.79–1158	0.997	5.0	101.2	12.0	105.4	1.16	5.79
Various	Buprenorphine	21.8	1.2	0.08–100	0.996	8.9	94.5	11.5	88.2	0.02	0.08
Drug precursor	Ephedrine/pseudoephedrine	7.2	1.0	0.10–500.0	0.997	4.1	94.0	3.4	97.3	0.03	0.10
	Norephedrine	6.3	0.9	0.50–1000	0.999	4.3	96.3	5.1	95.2	0.01	0.50
Anticancer	Azathioprine	7.8	0.9	0.10–490.0	0.999	7.6	97.5	13.9	97.4	0.03	0.10
	Methotrexate	7.9	1.0	0.92–458.0	0.997	8.7	108.0	4.1	112.2	0.28	0.92
	Ifosfamide	12.7	1.1	0.05–509.0	0.999	2.4	93.6	2.7	95.3	0.01	0.05
	Tamoxifen	22.4	1.0	0.03–668.4	0.998	4.0	96.0	2.4	96.8	0.01	0.03
	Imatinib	15.4	0.8	0.88–88.4	0.994	2.5	103.8	1.5	101.3	0.08	0.28
	Capecitabine	16.1	0.9	0.01–594.6	0.999	2.3	89.2	2.8	89.7	0.001	0.004
	Bicalutamide	18.2	0.9	0.10–784.0	0.995	2.7	90.1	2.9	92.0	0.03	0.10
Anaesthetic and metabolite	Ketamine	10.6	1.0	0.05–500.0	0.998	1.8	92.5	1.3	93.6	0.01	0.05
	Norketamine	11.1	1.0	0.10–500.0	0.999	1.8	94.1	3.2	94.0	0.03	0.10
	Venlafaxine	14.1	1.3	0.04–434.8	0.998	2.5	91.2	1.7	90.5	0.01	0.04
	Desmethylvenlafaxine	10.8	1.0	0.10–500.0	0.998	2.8	101.3	2.1	102.3	0.03	0.10
	Fluoxetine	18.4	1.0	0.05–1000	0.999	1.7	96.8	1.8	98.3	0.01	0.05
	Norfluoxetine^b^	18.4	1.0	0.05 – 500.0	0.998	1.5	102.7	3.1	103.1	0.01	0.05
	Sertraline	19.2	1.0	0.05–500.0	1.000	1.6	95.3	1.7	95.7	0.01	0.05
	Mirtazapine^a^	13.5	1.0	0.05–100.0, 50.0–500.0	0.999/0.997	3.4	94.8	2.7	97.6	0.01	0.05
	Citalopram	15.1	1.0	0.50–1000	0.999	0.7	101.2	2.6	101.8	0.05	0.50
	Desmethylcitalopram	15.2	1.0	0.05–500.0	0.998	1.8	103.0	3.0	103.4	0.01	0.05
	Paroxetine	17.3	0.9	5.00–600	0.998	3.2	103.4	1.3	102.1	0.01	0.03
	Duloxetine	17.8	1.0	1.00–1000	0.997	3.0	91.2	13.6	78.3	0.003	0.01
	Amitriptyline	18.2	1.0	0.11–885.0	1.000	4.5	99.6	2.4	96.8	0.03	0.11
	Nortriptyline	18.4	1.0	0.22–800	0.999	4.0	95.5	3.1	92.9	0.07	0.22
	Norsertraline	19.8	1.0	0.23–100	0.999	8.7	99.0	11.0	91.8	0.07	0.23
Anti-epileptic	Carbamazepine	16.2	1.0	0.05–514.0	1.000	2.0	91.7	1.6	92.7	0.01	0.05
	Carbamazepine-10,11-epoxide	13.5	0.8	0.10–1000	0.997	1.6	88.9	2.1	89.9	0.03	0.10
	10,11-Dihydro-10-hydroxycarbamazepine	13.5	0.8	0.50–100.0	0.997	2.8	92.2	5.6	93.8	0.05	0.50
Calcium channel blocker	Diltiazem	16.7	1.0	0.10–486.2	0.996	2.3	92.7	2.3	93.6	0.01	0.10
	Verapamil	16.2	1.0	0.01–600	0.998	2.9	103.1	2.4	101.9	0.001	0.004
Hypnotic	Temazepam	18.2	1.0	0.05–500.0	0.998	1.0	97.0	1.6	97.9	0.01	0.05
	Oxazepam	17.8	1.0	0.10–800	0.999	3.3	94.8	3.4	94.3	0.02	0.08
	Diazepam	19.5	1.0	0.01–1000	1.000	1.6	100.7	4.5	99.6	0.003	0.01
Antipsychotic	Quetiapine	17.9	1.0	0.05–1000	0.997	1.4	95.3	1.2	96.4	0.01	0.05
	Risperidone	13.7	0.8	0.01–200	0.997	3.2	101.6	1.2	96.8	0.002	0.01
Dementia	Donepezil	13.9	0.9	0.01–1000	0.998	2.6	110.8	1.3	107.7	0.17	0.58
	Memantine	15.7	1.0	0.05–506.4	0.998	3.5	106.3	0.9	104.3	0.02	0.05
Human indicators	Creatinine	2.7	1.0	1.00–1000	0.999	1.4	100.5	2.8	100.1	0.30	1.00
	Nicotine	3.3	0.8	1.00–500.0	0.998	1.2	98.3	2.4	98.4	0.30	1.00
	Caffeine	8.3	1.2	0.50–500.0	0.999	1.7	99.6	2.8	100.4	0.10	0.50
	Cotinine	7.2	1.0	0.05–1000	0.999	1.5	98.4	1.5	98.8	0.01	0.05
	1,7-Dimethylxanthine^b^	6.8	0.9	1.00–500.0	0.999	6.0	94.3	9.9	94.9	0.30	1.00
Analgesics and metabolites	Morphine	3.5	1.0	1.00–500.0	0.998	2.9	99.1	2.5	97.5	0.30	1.00
	Dihydromorphine	3.3	1.0	0.05–500.0	0.997	4.4	106.0	2.7	108.5	0.01	0.05
	Normorphine	3.4	1.0	1.00–500.0	0.999	1.5	100.9	2.2	99.8	0.30	1.00
	Methadone	17.6	1.0	0.05–400.0	0.998	1.5	98.7	1.4	100.2	0.01	0.05
	EDDP	14.8	1.0	0.05–500.0	0.999	1.2	96.5	1.1	96.4	0.01	0.05
	Codeine	6.1	1.0	0.50–500.0	0.997	2.0	93.5	4.0	95.1	0.10	0.50
	Norcodeine	6.5	1.1	1.00–500.0	0.998	2.8	98.5	4.8	98.6	0.30	1.00
	Dihydrocodeine	5.5	0.9	0.10–500.0	0.999	1.6	94.2	2.1	94.6	0.03	0.10
	Tramadol	11.0	1.0	1.00–500.0	0.999	1.6	100.1	1.9	98.4	0.01	1.00
	*N*-Desmethyltramadol	11.9	1.1	0.50–500.0	0.998	2.5	92.5	2.2	94.4	0.01	0.50
	*O*-Desmethyltramadol	8.3	1.2	1.00–400.0	0.997	3.3	95.3	4.9	98.5	0.01	1.00
Stimulants and metabolites	Amphetamine	8.4	1.0	0.10–500.0	0.999	4.4	100.8	1.6	100.7	0.03	0.10
	Methamphetamine	8.5	1.0	0.10–500.0	0.999	2.2	101.0	1.3	101.1	0.03	0.10
	MDMA	8.6	1.0	0.05–1000	0.999	1.3	99.2	1.7	99.8	0.01	0.05
	MDA	8.6	1.0	0.10–1000	0.998	1.1	98.4	0.7	100.0	0.03	0.10
	Cocaine	11.3	1.0	0.05–500.0	0.999	2.2	97.2	1.5	99.0	0.01	0.05
	Benzoylecgonine^a^	9.7	1.0	0.05–100.0, 50.0–500.0	0.998/0.999	2.4	103.4	0.9	103.2	0.01	0.05
	Anhydroecgonine methyl ester	3.5	1.3	0.50–500.0	0.999	2.3	101.1	2.4	98.7	0.10	0.50
	Cocaethylene	12.9	1.0	0.05–500.0	0.999	2.8	95.1	1.7	94.7	0.01	0.05
	Mephedrone	9.8	1.0	0.05–500.0	0.998	1.8	87.1	2.9	85.7	0.01	0.05
	MDPV	12.1	0.9	0.05–500.0	0.999	2.2	99.6	0.7	101.4	0.01	0.05
Opioid and metabolite	Heroin	10.9	1.0	0.50–500.0	0.999	1.9	98.2	1.8	99.3	0.10	0.50
	6-Acetylmorphine	7.7	1.1	0.10–500.0	0.997	6.1	95.3	5.1	100.1	0.03	0.10
Pesticides, fungicides and herbicides	Thiamethoxam	8.3	0.4	1.00–100	0.994	4.7	93.8	5.4	96.9	0.02	0.06
	Imidacloprid	10.1	0.6	0.10–595.2	0.996	2.8	100.5	5.5	103.5	0.01	0.04
	Clothianidin	10.4	0.5	1.00–800	0.999	3.2	97.9	3.3	98.6	0.01	0.04
	Metazachlor	17.1	1.0	0.05–1011	0.999	2.5	106.0	2.6	104.7	0.004	0.01
	Terbuthylazine	19.3	1.0	0.05–519	1.000	2.4	99.8	3.3	97.5	0.01	0.02
	Methiocarb	19.4	1.0	0.08–1007	0.999	1.9	101.8	1.8	100.6	0.02	0.08
	Dichlofluanid	20.4	1.1	6.83–1092	0.994	3.8	94.9	4.4	90.9	1.29	4.30
	Flufenacet	20.5	1.2	0.01–986.0	0.997	2.0	104.2	2.9	106.2	0.002	0.01
	Oxadiazon	24.2	1.2	1.00–99.6	0.996	4.0	95.5	2.8	97.1	0.02	0.08
	Chlorpyrifos^c^	24.8	1.5	1.87–98.5	0.985	11.8	80.7	7.8	83.3	0.56	1.87
	Triallate	24.9	1.3	0.03–79.0	0.992	7.6	81.3	13.2	70.6	0.01	0.03
Veterinary pharmaceuticals	Tylosin	17.3	1.0	0.56–560.0	0.999	2.2	99.5	4.0	100.2	0.11	0.56
	Sulfapyridine	6.4	1.2	0.05–800	0.999	2.6	110.7	1.1	109.5	0.01	0.03
	Sarafloxacin	10.9	0.7	0.88–442	0.995	5.2	112.1	2.3	107.1	0.22	0.75
	Ceftiofur	12.1	1.3	0.28–800.0	0.993	3.6	89.5	2.0	86.4	0.08	0.28
	Diazinon	21.9	1.2	0.11–2100	0.998	2.7	98.9	4.1	96.0	0.01	0.02

Where possible, instrumental performance was determined at concentrations of 10 μg L^−1^, 100 μg L^−1^ and 500 μg L^−1^; i.e. those analytes where these concentrations were outside the range of linearity or results were <LOQ were not included

*IDL* instrumental detection limit, *IQL* instrumental quantification limit

^a^Linear range was split into two overlapping ranges to ensure the *r*^2^ value ≥ 0.997

^b^Semi-quantitative, due to only one MRM transition

^c^Semi-quantitative, due to poor *r*^2^ value

For the linear response, a linear range covering several orders of magnitude with the *r*^2^ value of ≥ 0.997 was ideal. However, for a few analytes (triclosan, benzoylecgonine and mirtazapine), this could only be achieved through splitting the linear range into two overlapping ranges, each with the *r*^2^ value ≥ 0.997. This allowed adequate quantification across the entire range. Of the 142 compounds, 120 have *r*^2^ values ≥ 0.997. Twenty-one of the remaining compounds have *r*^2^ values ≥ 0.992. Although this is not ideal, it is still adequate for accurate quantification, as the other parameters indicate. Chlorpyrifos has the lowest *r*^2^ value, likely due to the lack of an analogous internal standard; however, it passes further instrumental performance criteria.

The intra-day instrumental performance is high across many compounds. Out of the 142 analytes, the majority of which are very precise, with a deviation of ≤ 5%. The precision of 20 compounds was between 5 and 10%, and only one compound was > 10% (chlorpyrifos (11.8%), likely due to poor *r*^2^). However, all deviations are inside the recommended maximum of 20%. For 119/142 compounds, the accuracy lay within the ideal range of 90–110%. Of the remaining compounds, only triclosan has an inaccuracy of > 20%.

Regarding the inter-day instrumental performance, the precision is high across many analytes with 115 analytes with a deviation of ≤ 5%. Of the 27 analytes with > 5% deviation, only 8 were > 10%. The inter-day accuracy of these compounds was also high, with only 20 compounds that deviated from the QC by > 10%, 4 of which were only slightly greater than 20%. Data for both intra- and inter-day precision and accuracy can be found ESM Table S7.

IDLs ranged from 0.4 ng L^−1^ (bisoprolol) to 4783 ng L^−1^ (cefalexin), and IQLs ranged from 1.2 ng L^−1^ (bisoprolol) to 15,940 ng L^−1^ (cefalexin). Whilst many of these IQL concentrations are very low, the samples may still need to be concentrated (utilising SPE), as the concentrations of most compounds in environmental matrices are likely to be even lower.

### Method performance

Sample extraction was carried out using SPE following the method shown in Fig. 1 (sections “Extraction for liquid matrices” and “Extraction for solid matrices”) and has shown good extraction performance for many CECs. The Oasis HLB sorbent is essential to multi-residue methods, as it has the ability to retain a large range of polar analytes at neutral pH and is therefore widely used in analysis of environmental matrices. However, two drawbacks have been found in the use of this sorbent. Firstly, HLB might result in low recovery of very polar compounds such as metformin and creatinine. This is easily remedied, as metformin and creatinine along with acetaminophen, nicotine, caffeine and 1,7-dimethylxanthine are present in the environment as such high levels that direct injection are utilised instead. The second drawback of HLB is its lack of selectivity. Whilst enabling the extraction of a large variety of polar analytes, in complex matrices, much of the matrix can be extracted along with the chosen analytes, causing significant signal interference. It is therefore important to assess matrix suppression. Previously, it was found that the use of HLB with digested solids provided poor results. This not only necessitated the use of an alternative sorbent, MCX, but also splitting the eluents into acidic and basic fractions (see Fig. 1) [39].

**Fig. 2:**
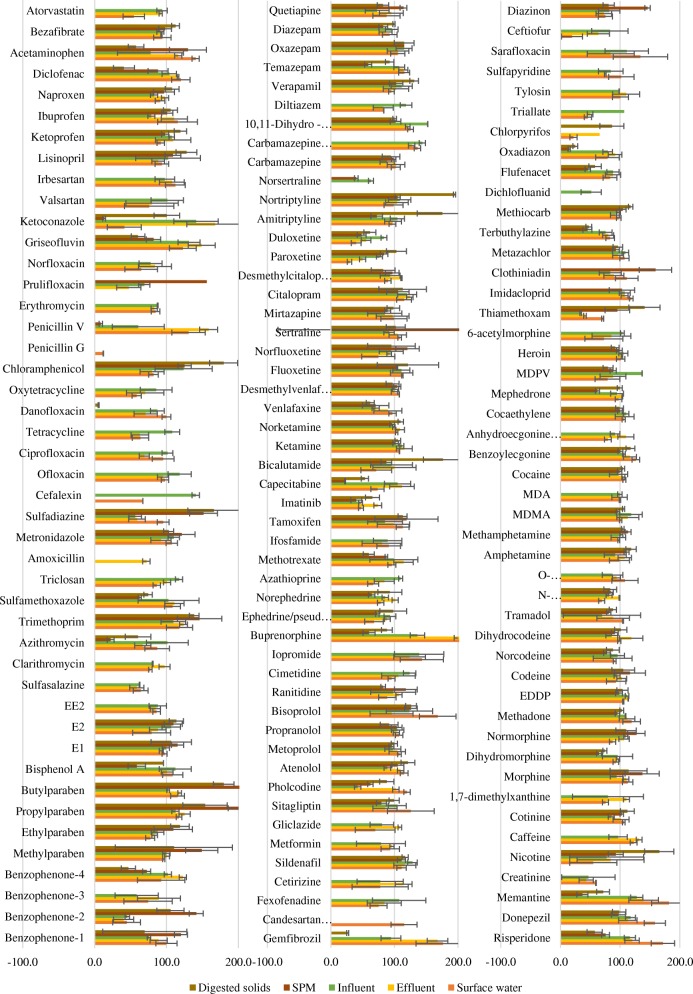
Relative recoveries for all matrices. Error bars show the range of standard deviation

Figure 2 shows relative recoveries for all matrices: surface water, effluent, influent, SPM and digested solids. These very different matrices exhibit similar ranges of relative recovery. Most of the compounds had recoveries between 80 and 120%. Due to the complexity of environmental matrices, not all compounds were recovered adequately across all matrices; therefore, the number of compounds accurately quantifiable in each matrix varies from 138 analytes in surface water to 96 analytes in digested solids. The data for relative and absolute recoveries for all matrices can be found in ESM Tables S8 and S9 and Fig. S2.

**Fig. 3:**
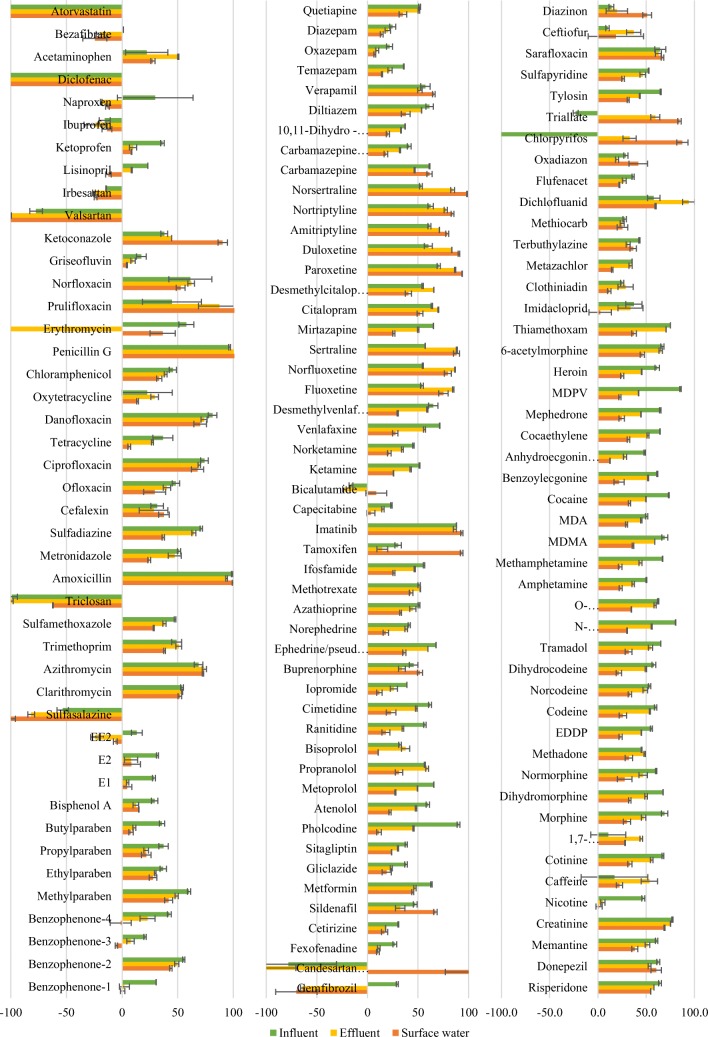
Matrix suppression for all liquid matrices. Error bars show the range of standard deviation

Matrix suppression was analysed for liquid matrices (see Fig. 3 and ESM Table S10). Proximity to zero shows limited matrix effects. Most analytes experienced matrix suppression, shown in Fig. 3 as a positive percentage. For all matrices, at least 108 analytes were below 70% suppression. However, a few compounds, primarily from ESI−, experienced significant (> 20%) signal enhancement. Some compounds experienced signal enhancement in some matrices and suppression in others (naproxen, erythromycin, EE2, bicalutamide, candesartan cilexetil, gemfibrozil, chlorpyrifos). This considerable variation is to be expected for a multi-residue method with this variety of analytes. It especially highlights the importance of using relevant analogous internal standards, as well as thoroughly exploring the different matrices relevant to this work.

The method accuracy of the method was 107.0%, and the precision was 13.4%. MQLs for liquid matrices range from 0.004 ng L^−1^ for bisoprolol, in surface water, to 3118 ng L^−1^ for creatinine, in influent (Table 2). For the remaining analytes, there is not enough environmental data on their presence in the UK to comment on whether the MDLs and MQLs are low enough to measure these levels in the catchment of interest. Therefore, this method was applied to five different matrices at a WwTW in the South West UK, which were influent, effluent, surface water, SPM and digested solids, to underline the utilisation of this method to relevant environmental matrices.

**Table 2:** Method performance data for ECs of interest in the mobile phase (ordered by class)

Class of analyte	Analyte	Surface water (ng L^−1^)		Effluent (ng L^−1^)		Influent (ng L^−1^)		Solid particulate matter (ng g^−1^)		Digested solids (ng g^−1^)	
		MDL	MQL	MDL	MQL	MDL	MQL	MDL	MQL	MDL	MQL
UV filter	Benzophenone-1	0.07	0.35	0.14	0.71	0.23	1.15	0.004	0.02	0.14	0.70
	Benzophenone-2	0.16	0.79	0.34	1.68	0.36	1.82	0.004	0.02	0.09	0.44
	Benzophenone-3	0.15	0.77	0.19	0.97	0.37	1.87	–	–	–	–
	Benzophenone-4	2.09	6.90	5.78	19.1	7.83	25.8	0.21	0.70	4.01	13.2
Parabens	Methylparaben	0.08	0.40	0.19	0.94	0.28	1.41	0.003	0.02	0.06	0.31
	Ethylparaben	0.24	0.79	0.46	1.52	0.49	1.61	0.01	0.05	0.17	0.57
	Propylparaben	0.25	0.83	0.47	1.54	0.63	2.08	0.01	0.03	0.22	0.72
	Butylparaben	0.08	0.38	0.14	0.71	0.24	1.21	0.002	0.01	0.10	0.52
Plasticizer	Bisphenol A	0.26	0.86	0.56	1.84	0.85	2.79	0.03	0.09	0.27	0.88
Steroid estrogens	E1	0.78	3.92	0.15	7.69	1.96	9.78	0.04	0.21	1.68	8.38
	E2	0.90	4.48	1.41	7.03	1.84	9.22	0.04	0.21	1.48	7.41
	EE2	0.98	4.91	1.46	7.32	1.83	9.15	–	–	–	–
Antibiotics and antibacterials	Sulfasalazine	4.31	14.2	9.66	31.9	12.6	41.4	–	–	–	–
	Clarithromycin	0.18	0.90	0.28	1.40	0.34	1.69	–	–	–	–
	Azithromycin	0.08	0.26	0.21	0.68	0.14	0.45	0.03	0.10	0.01	0.04
	Trimethoprim	0.26	0.85	0.51	1.67	0.73	2.41	0.01	0.03	0.07	0.22
	Sulfamethoxazole	0.19	0.63	0.47	1.56	0.72	2.38	0.02	0.08	0.12	0.41
	Triclosan	2.93	9.68	4.55	15.0	4.93	16.3	–	–	–	–
	Amoxicillin	–	–	0.26	0.86	–	–	–	–		
	Metronidazole	0.29	0.98	0.68	2.27	0.57	1.90	0.03	0.09	0.03	0.10
	Sulfadiazine	0.05	0.18	0.18	0.59	0.18	0.62	0.003	0.01	0.003	0.01
	Cefalexin	35.6	118.7	10.2	33.9	18.9	63.1	–	–	–	–
	Ofloxacin	0.35	1.17	0.72	2.40	0.58	1.93	–	–	–	–
	Ciprofloxacin	1.85	6.17	5.10	17.0	3.48	11.6	–	–	–	–
	Tetracycline	0.15	0.50	0.30	1.01	0.18	0.59	–	–	–	–
	Danofloxacin	1.58	5.28	4.45	14.85	3.62	12.08	–	–	2.84	9.45
	Oxytetracycline	6.04	20.1	10.1	33.6	8.26	27.5	–	–	–	–
	Chloramphenicol	3.18	10.6	6.52	21.7	4.21	14.0	0.21	0.69	0.15	0.48
	Penicillin G	0.89	2.98	–	–	–	–	–	–	–	–
	Penicillin V	0.56	1.86	0.92	3.06	2.40	8.00	0.84	2.80	–	–
	Erythromycin	1.15	3.83	2.35	7.85	2.22	7.41	–	–	–	–
	Prulifloxacin	–	–	51.3	171.0	35.3	117.6	–	–	–	–
	Norfloxacin	0.01	0.04	0.02	0.06	0.02	0.07	–	–	–	–
Antifungal	Griseofulvin	0.32	1.06	0.52	1.74	0.59	1.98	0.05	0.16	0.06	0.21
	Ketoconazole	0.06	0.21	0.03	0.10	0.04	0.12	0.02	0.07	0.00	0.01
Hypertension	Valsartan	2.81	9.26	6.40	21.1	7.24	23.9	–	–	–	–
	Irbesartan	0.89	4.47	1.88	9.38	2.50	12.5	–	–	–	–
	Lisinopril	2.17	21.7	4.25	42.5	3.25	32.5	0.04	0.43	0.25	2.47
NSAIDs	Ketoprofen	0.74	3.72	1.60	8.00	2.38	11.9	0.06	0.28	0.47	2.35
	Ibuprofen	0.06	0.31	0.08	0.42	0.19	0.93	0.005	0.02	0.07	0.36
	Naproxen	0.61	3.07	1.17	5.85	6.29	31.5	0.05	0.25	0.60	3.02
	Diclofenac	0.22	0.73	0.44	1.44	0.67	2.22	0.02	0.06	0.75	2.46
	Acetaminophen	1.20	6.02	2.39	12.0	138.0*	1017*	0.04	0.21	2.74	13.7
Lipid regulator	Bezafibrate	0.22	0.66	0.38	1.25	0.64	2.11	0.02	0.05	0.18	0.60
	Atorvastatin	0.14	0.70	0.17	0.84	0.17	0.85	–	–	–	–
Antihyperlipidemic	Gemfibrozil	0.30	1.00	0.63	2.11	1.12	3.75	–	–	0.20	0.67
Antihyperintensive	Candesartan Cilexetil	6.89	23.0	–	–	–	–	–	–	–	–
Antihistamine	Fexofenadine	0.21	0.69	0.40	1.32	0.56	1.85	–	–	–	–
	Cetirizine	0.26	0.87	0.32	1.06	0.52	1.72	–	–	–	–
GUD/ED	Sildenafil	0.01	0.03	0.02	0.05	0.01	0.05	0.001	0.003	0.001	0.003
Diabetes	Metformin	156.0*	515.0*	163.0*	460.0*	457.0*	1509*	–	–	–	–
	Gliclazide	0.15	0.77	0.16	0.82	0.22	1.09	–	–	–	–
	Sitagliptin	0.03	0.09	0.08	0.27	0.06	0.22	0.004	0.01	0.003	0.01
Cough suppressant	Pholcodine	2.25	7.42	8.02	26.5	25.3	83.3	0.28	0.92	1.52	5.00
Beta blocker	Atenolol	0.20	0.66	0.56	1.84	0.71	2.35	0.01	0.05	0.10	0.33
	Metoprolol	0.07	0.35	0.19	0.96	0.28	1.40	0.01	0.03	0.03	0.14
	Propranolol	0.29	0.96	0.73	2.41	0.68	2.25	0.01	0.04	0.13	0.42
	Bisoprolol	0.001	0.004	0.004	0.01	0.003	0.01	0.0001	0.0005	0.0001	0.0005
H_2_ receptor agonist	Ranitidine	7.96	39.8	22.3	111.4	14.8	73.8	0.44	2.19	4.81	24.1
	Cimetidine	1.60	7.98	3.12	15.6	5.06	25.3	–	–	–	–
X-ray contrast media	Iopromide	5.97	29.9	14.1	70.6	24.5	123.0	–	–	–	–
Various	Buprenorphine	0.06	0.20	0.11	0.36	0.18	0.61	0.02	0.07	0.01	0.05
Drug precursor	Ephedrine/pseudoephedrine	0.60	1.97	1.62	5.36	1.32	4.36	0.02	0.07	0.11	0.35
	Norephedrine	0.18	8.82	0.35	17.3	0.37	18.6	0.01	0.39	0.04	1.85
Anticancer	Azathioprine	0.17	0.55	0.36	1.20	0.41	1.36	–	–	–	–
	Methotrexate	6.13	20.2	9.04	29.8	7.11	23.5	0.16	0.53	1.64	5.42
	Ifosfamide	0.08	0.40	0.24	1.22	0.31	1.53	–	–	–	–
	Tamoxifen	14.5	72.6	0.76	3.82	0.70	3.50	0.004	0.01	2.23	11.14
	Imatinib	0.88	2.93	1.13	3.76	1.78	5.95	0.10	0.35	0.06	0.21
	Capecitabine	0.01	0.02	0.01	0.03	0.01	0.03	0.002	0.01	0.001	0.003
	Bicalutamide	0.22	0.72	0.31	1.02	0.32	1.07	0.02	0.06	0.01	0.03
Anaesthetic and metabolite	Ketamine	0.07	0.37	0.19	0.93	0.24	1.20	0.005	0.02	0.03	0.17
	Norketamine	0.23	0.76	0.56	1.86	0.72	2.37	0.02	0.05	0.10	0.33
	Venlafaxine	0.07	0.37	0.24	1.20	0.37	1.83	0.01	0.03	0.08	0.38
	Desmethylvenlafaxine	0.24	0.80	0.66	2.18	0.85	2.79	0.01	0.05	0.09	0.29
	Fluoxetine	1.14	5.71	1.42	7.08	0.50	2.52	0.005	0.02	0.11	0.53
	Norfluoxetine	1.64	8.21	1.27	6.35	0.42	2.12	0.004	0.02	0.14	0.68
	Sertraline	1.61	8.07	1.21	6.05	0.74	3.72	0.002	0.01	0.17	0.86
	Mirtazapine	0.09	0.44	0.25	1.25	0.39	1.94	0.01	0.03	0.05	0.27
	Citalopram	0.61	6.08	1.41	14.1	1.24	12.4	0.02	0.24	0.16	1.64
	Desmethylcitalopram	0.14	0.69	0.36	1.82	0.31	1.54	0.01	0.03	0.05	0.24
	Paroxetine	0.18	0.59	0.21	0.69	0.13	0.45	0.01	0.02	0.005	0.02
	Duloxetine	0.04	0.13	0.05	0.18	0.04	0.12	0.003	0.01	0.002	0.01
	Amitriptyline	0.16	0.55	0.33	1.09	0.30	1.02	0.02	0.07	0.01	0.03
	Nortriptyline	0.33	1.11	0.63	2.11	0.61	2.03	0.03	0.10	0.02	0.06
	Norsertraline	–	–	–	–	1.07	3.58	0.09	0.28	–	–
Anti-epileptic	Carbamazepine	0.08	0.38	0.19	0.93	0.27	1.37	0.01	0.03	0.10	0.48
	Carbamazepine-10,11-epoxide	0.16	0.53	0.55	1.82	0.53	1.76	–	–	–	–
	10,11-Dihydro-10-hydroxycarbamazepine	0.34	3.37	0.84	8.41	0.99	9.94	0.02	0.25	0.43	4.35
Calcium channel blocker	Diltiazem	0.11	1.11	0.32	3.23	0.27	2.68	–	–	–	–
	Verapamil	0.01	0.02	0.01	0.04	0.01	0.03	0.001	0.002	0.0004	0.001
Hypnotic	Temazepam	0.08	0.38	0.14	0.69	0.18	0.92	0.01	0.04	0.16	0.82
	Oxazepam	0.11	0.36	0.22	0.72	0.20	0.66	–	–	0.01	0.03
	Diazepam	0.02	0.06	0.04	0.13	0.04	0.12	0.002	0.01	0.002	0.01
Antipsychotic	Quetiapine	0.10	0.48	0.21	1.07	0.26	1.32	0.004	0.02	0.05	0.26
	Risperidone	0.01	0.02	0.02	0.06	0.02	0.06	0.001	0.004	0.002	0.01
Dementia	Donepezil	0.55	1.83	1.54	5.12	1.48	4.93	0.09	0.30	0.09	0.29
	Memantine	0.04	0.14	0.11	0.36	0.12	0.39	0.02	0.07	0.01	0.04
Human indicators	Creatinine	511*	1686*	771*	2544*	945*	3118*	–	–	–	–
	Nicotine	3.34	11.0	5.44	18.0	508*	2296*	0.16	–	0.66	2.19
	Caffeine	0.37	1.83	1.11	5.57	121*	581*	–	–	–	–
	Cotinine	0.07	0.35	0.21	1.06	0.27	1.34	0.005	0.02	0.24	1.22
	1,7-Dimethylxanthine	3.19	10.5	11.4	37.6	560*	2165*	–	–	–	–
Analgesics and metabolites	Morphine	2.65	8.75	6.34	20.9	8.85	29.2	0.11	0.37	1.92	6.33
	Dihydromorphine	0.11	0.55	0.32	1.59	0.05	2.51	0.01	0.04	0.09	0.45
	Normorphine	3.54	11.7	7.84	25.9	9.99	33.0	0.12	0.39	1.74	5.75
	Methadone	0.11	0.54	0.21	1.04	0.20	1.01	0.01	0.03	0.03	0.17
	EDDP	0.21	1.05	0.29	1.47	0.23	1.13	0.01	0.03	0.04	0.20
	Codeine	0.74	3.71	1.46	7.31	2.56	12.8	0.04	0.21	0.33	1.66
	Norcodeine	2.88	9.52	8.32	27.4	8.53	28.2	0.19	0.64	1.26	4.17
	Dihydrocodeine	0.23	0.75	0.55	1.83	0.88	2.89	0.02	0.05	0.11	0.36
	Tramadol	0.08	8.20	0.21	21.3	0.30	30.0	0.01	0.62	0.03	3.26
	*N*-Desmethyltramadol	0.12	5.92	0.30	15.0	0.56	27.9	0.01	0.30	0.04	2.02
	*O*-Desmethyltramadol	0.09	8.53	0.28	27.8	0.31	31.4	–	–	–	–
Stimulants and metabolites	Amphetamine	0.68	2.23	1.11	3.65	1.23	4.07	0.01	0.05	0.09	0.29
	Methamphetamine	0.32	1.05	0.71	2.35	0.95	3.13	0.01	0.04	0.09	0.30
	MDMA	0.10	0.50	0.27	1.35	0.34	1.70	0.01	0.03	0.04	0.18
	MDA	0.53	1.74	1.00	3.30	0.99	3.26	–	–	–	–
	Cocaine	0.07	0.35	0.22	1.11	0.46	2.31	0.01	0.03	0.03	0.15
	Benzoylecgonine	0.07	0.34	0.18	0.91	0.21	1.07	0.005	0.02	0.03	0.14
	Anhydroecgonine methyl ester	0.93	4.67	1.99	9.96	2.95	14.8	–	–	–	–
	Cocaethylene	0.07	0.35	0.21	1.04	1.31	6.54	0.01	0.03	0.03	0.17
	Mephedrone	0.22	1.09	0.44	2.19	0.55	2.75	0.01	0.04	0.06	0.31
	MDPV	0.04	0.22	0.12	0.59	0.48	2.41	0.01	0.03	0.04	0.20
Opioid and metabolite	Heroin	0.92	4.62	3.44	17.2	4.18	20.9	0.05	0.25	0.56	2.79
	6-Acetylmorphine	0.28	0.94	0.76	2.50	0.89	2.95	–	–	–	–
Pesticides, fungicides and herbicides	Thiamethoxam	0.13	0.42	0.44	1.46	0.53	1.76	0.01	0.03	0.01	0.02
	Imidacloprid	0.04	0.15	0.10	0.33	0.10	0.33	0.01	0.02	–	–
	Clothianidin	0.06	0.19	0.14	0.47	0.15	0.50	0.004	0.01	–	–
	Metazachlor	0.02	0.06	0.04	0.14	0.04	0.13	0.002	0.01	0.002	0.01
	Terbuthylazine	0.03	0.11	0.07	0.22	0.07	0.23	0.01	0.02	0.01	0.02
	Methiocarb	0.13	0.43	0.27	0.91	0.26	0.86	0.01	0.04	0.01	0.04
	Dichlofluanid	–	–	–	–	25.2	83.8	–	–	–	–
	Flufenacet	0.01	0.04	0.02	0.07	0.02	0.07	0.002	0.01	0.002	0.01
	Oxadiazon	0.15	0.49	0.26	0.85	0.30	0.98	0.08	0.26	0.05	0.16
	Chlorpyrifos	12.9	42.9	8.54	28.5	–	–	–	–	0.33	1.09
	Triallate	0.11	0.37	0.20	0.68	0.09	0.31	–	–	–	–
Veterinary pharmaceuticals	Tylosin	1.28	6.39	2.23	11.1	3.27	16.3	–	–	–	–
	Sulfapyridine	0.04	0.14	0.11	0.37	0.10	0.33	–	–	–	–
	Sarafloxacin	0.83	2.78	2.66	8.86	2.01	6.72	–	–	–	–
	Ceftiofur	2.17	7.23	1.32	4.41	1.02	3.39	–	–	–	–
	Diazinon	0.03	0.11	0.07	0.23	0.06	0.21	0.00	0.01	0.003	0.01

*Calculated for direct injection

Overall, this method provides a clear benefit when used in a catchment-based study, compared to more complex approaches with multiple sample preparation and analytical methods for different classes of CECs from the same matrices. The main advantage of the multi-residue method presented in this paper is the applicability of this method to liquid and solid matrices. In particular, SPM, which is an often forgotten but critical aspect of wastewater, as it contains many more hydrophobic compounds at significant concentrations within this matrix [28, 42, 43].

### Application to environmental matrices

The data is presented as average concentrations with variation across a week (Table 3 and ESM Tables S11–S15). As expected, the liquid influent fraction shows the highest variation in concentrations both across the week and between different CECs. Of the 138 analytes of this method, 70% were quantified in influent at this site. Creatinine had the highest average concentration of any analyte in this study (1.3 ± 0.3 mg L^−1^) and is often used a human indicator due to its correlation to population [44, 45]. Other human indicators were present at high levels throughout the week. The other CECs were present at a range of concentrations. Methiocarb, for example, is present intermittently across the week with an average concentration of 3.7 ± 0.6 ng L^−1^, which is very close to its MQL of 0.86 ng L^−1^. However, acetaminophen reaches concentrations several magnitudes higher than methiocarb, at 393.6 ± 100.0 μg L^−1^. This is slightly higher than those published in a review by Verlicchi et al. [11] which had an absolute highest concentration of 246 μg L^−1^. It is interesting to see the presence of the (pesticide) methiocarb in influent, as this is generally used for plant protection, particularly against slugs. It is thought to be an unlikely compound to make its way into influent and thought to enter surface waters via direct application to the environment. Its presence in the sewage treatment works, although in a low concentration, is notable as it may indicate incorrect disposal, although a higher concentration would be expected from this. It was recently banned for use as a molluscicide in 2014, the grace period of which ended the month before these samples were collected; however, it could still be used as an insect repellent and seed treatment [46]. Other pesticides found in influent at this site include imidacloprid and flufenacet, which are more widely used for vegetable and fruit crops and may be due to rinsing of food prior to consumption. However, this needs further detailed investigation.

**Table 3:** Weekly average concentrations of ECs found in several matrices at site A (mean concentration ± variation from mean across the week)

Class of analyte	Compound	Surface water (ng L^−1^)	Effluent (ng L^−1^)	Influent (ng L^−1^)	SPM (ng L^−1^)	Digested solids (ng g^−1^)
UV filter	Benzophenone-1	<MQL	<MQL	949.2 ± 1134.5	13.8 ± 12.0	N/A
	Benzophenone-2	<MQL	62.0 ± 36.0	1701.0 ± 2176.6	22.0 ± 25.9	10.2 ± 5.2
	Benzophenone-3	19.0 ± 1.9	96.7 ± 36.7	2051.5 ± 897.3	N/A	N/A
	Benzophenone-4	607.9 ± 344.8	4224.7 ± 3455.7	15,817.4 ± 13,404.6	9.9 ± 4.0	<MQL
Parabens	Methylparaben	7.0 ± 2.5	42.3 ± 49.4	23,401.4 ± 36,282.5	122.1 ± 99.8	360.2 ± 132.5
	Ethylparaben	<MQL	<MQL	2166.0 ± 2427.8	21.0 ± 17.8	<MQL
	Propylparaben	3.7 ± 0.7	32.1 ± 11.4	5456.2 ± 3190.2	33.2 ± 32.6	<MQL
	Butylparaben	<MQL	<MQL	337.4 ± 434.5	40.3 ± 66.7	<MQL
Plasticizer	Bisphenol-A	38.1 ± 15.1	473.6 ± 256.2	20,395.5 ± 15,740.7	1383.9 ± 1204.0	4365.6 ± 309.6
Steroid estrogens	E1	<MQL	6.3 ± 1.6	47.0 ± 10.7	<MQL	41.5 ± 2.9
	E2	<MQL	<MQL	<MQL	<MQL	<MQL
	EE2	<MQL	<MQL	<MQL	N/A	N/A
Antibiotics and antibacterials	Sulfasalazine	53.6 ± 6.8	241.4 ± 27.5	612.9 ± 248.7	N/A	N/A
	Clarithromycin	102.0 ± 26.8	776.2 ± 121.4	1321.1 ± 293.4	N/A	N/A
	Azithromycin	3.9 ± 3.2	108.7 ± 48.4	793.1 ± 256.1	<MQL	5.7 ± 5.5
	Trimethoprim	25.9 ± 5.7	175.6 ± 30.2	626.2 ± 151.9	16.7 ± 6.3	11.1 ± 2.8
	Sulfamethoxazole	33.2 ± 3.1	54.3 ± 6.2	126.7 ± 58.6	<MQL	<MQL
	Triclosan	<MQL	220.9 ± 52.2	2480.5 ± 801.6	N/A	N/A
	Amoxicillin	N/A	<MQL	N/A	N/A	N/A
	Metronidazole	10.1 ± N/A	37.0 ± 6.5	81.6 ± 23.6	<MQL	<MQL
	Sulfadiazine	<MQL	<MQL	<MQL	8.5 ± 3.3	<MQL
	Cefalexin	N/A	N/A	N/A	N/A	N/A
	Ofloxacin	71.5 ± 29.5	108.7 ± 52.7	<MQL	N/A	N/A
	Ciprofloxacin	N/A	<MQL	N/A	N/A	N/A
	Tetracycline	<MQL	<MQL	251.5 ± 70.5	N/A	N/A
	Danofloxacin	<MQL	<MQL	372.3 ± 414.1	N/A	<MQL
	Oxytetracycline	<MQL	<MQL	N/A	N/A	N/A
	Chloramphenicol	<MQL	157.9 ± 23.3	<MQL	<MQL	377.6 ± 46.3
	Penicillin G	N/A	<MQL	<MQL	N/A	N/A
	Penicillin V	<MQL	<MQL	<MQL	<MQL	N/A
	Erythromycin	2148.2 ± 168.5	6503.8 ± 619.1	13,591.5 ± 2445.9	N/A	N/A
	Prulifloxacin	N/A	<MQL	N/A	N/A	N/A
	Norfloxacin	N/A	N/A	N/A	N/A	N/A
Antifungal	Griseofulvin	<MQL	<MQL	<MQL	1.4 ± N/A	<MQL
	Ketoconazole	46.5 ± 6.1	64.3 ± 5.2	131.0 ± 40.9	171.6 ± 48.3	929.3 ± 212.1
Hypertension	Valsartan	<MQL	236.9 ± 23.0	854.9 ± 306.9	N/A	N/A
	Irbesartan	111.4 ± 14.6	287.9 ± 30.0	380.7 ± 73.3	N/A	N/A
	Lisinopril	<MQL	93.9 ± 16.1	890.5 ± 273.9	12.3 ± 2.7	<MQL
NSAIDs	Ketoprofen	<MQL	<MQL	<MQL	<MQL	<MQL
	Ibuprofen	60.5 ± 21.3	1029.2 ± 357.7	18,840.6 ± 5449.2	42.8 ± 15.9	231.8 ± 39.9
	Naproxen	234.9 ± 47.7	1452.5 ± 213.7	15,238.5 ± 3111.2	51.7 ± 17.9	108.4 ± 5.3
	Diclofenac	88.2 ± 7.3	345.6 ± 52.7	916.2 ± 367.3	<MQL	26.2 ± 3.4
	Acetaminophen	193.0 ± 49.7	1840.7 ± 449.3	393,559.0 ± 100,020.4	28.1 ± 23.7	<MQL
Lipid regulator	Bezafibrate	103.6 ± 15.8	591.7 ± 76.7	2038.3 ± 468.2	2.8 ± 1.2	7.5 ± 0.7
	Atorvastatin	44.4 ± 8.1	172.9 ± 27.0	1338.2 ± 456.4	N/A	N/A
Antihyperlipidaemic	Gemfibrozil	N/A	<MQL	N/A	N/A	849.6 ± 183.8
Antihyperintensive	Candesartan Cilexetil	N/A	N/A	N/A	N/A	N/A
Antihistamine	Fexofenadine	209.1 ± 31.9	732.6 ± 103.2	1012.1 ± 689.0	N/A	N/A
	Cetirizine	239.6 ± 35.8	1051.1 ± 154.5	1088.4 ± 106.6	N/A	N/A
GUD/ED	Sildenafil	2.2 ± 1.3	25.5 ± 12.4	29.0 ± 41.6	2.6 ± 2.9	20.8 ± 1.0
Diabetes	Metformin	3607.1 ± 413.6	14,050.8 ± 897.2	117,927.4 ± 24,688.7	N/A	N/A
	Gliclazide	47.3 ± 7.6	84.3 ± 7.5	136.4 ± 41.6	N/A	N/A
	Sitagliptin	142.5 ± 13.5	441.4 ± 67.2	524.7 ± 76.8	11.2 ± 2.9	27.6 ± 2.1
Cough suppressant	Pholcodine	<MQL	<MQL	<MQL	<MQL	<MQL
Beta blocker	Atenolol	53.9 ± 10.6	328.9 ± 31.4	2581.7 ± 584.4	23.3 ± 9.6	<MQL
	Metoprolol	<MQL	13.3 ± 2.9	32.1 ± 9.3	<MQL	<MQL
	Propranolol	20.6 ± 3.5	106.6 ± 6.1	182.3 ± 30.9	21.5 ± 6.4	198.5 ± 22.9
	Bisoprolol	<MQL	14.7 ± N/A	30.6 ± 29.3	<MQL	<MQL
H_2_ receptor agonist	Ranitidine	148.2 ± 31.8	1163.0 ± 98.1	1545.0 ± 277.6	<MQL	<MQL
	Cimetidine	<MQL	<MQL	170.3 ± 64.4	N/A	N/A
X-ray contrast media	Iopromide	<MQL	<MQL	<MQL	N/A	N/A
Various	Buprenorphine	6.3 ± 0.2	5.7 ± N/A	60.8 ± 37.7	0.6 ± 0.2	13.2 ± 4.5
Drug precursor	Ephedrine/pseudoephedrine	25.6 ± 2.0	115.5 ± 18.6	655.5 ± 248.6	4.5 ± 1.3	<MQL
	Norephedrine	<MQL	<MQL	<MQL	<MQL	<MQL
Anticancer	Azathioprine	<MQL	<MQL	<MQL	N/A	N/A
	Methotrexate	<MQL	<MQL	<MQL	<MQL	<MQL
	Ifosfamide	<MQL	<MQL	<MQL	N/A	N/A
	Tamoxifen	<MQL	<MQL	<MQL	<MQL	<MQL
	Imatinib	38.3 ± 1.3	143.3 ± 39.6	88.8 ± 14.4	47.6 ± 17.1	123.0 ± 40.3
	Capecitabine	<MQL	<MQL	7.3 ± 2.8	<MQL	<MQL
	Bicalutamide	59.4 ± 1.1	115.6 ± 6.7	154.3 ± 10.0	7.5 ± 2.0	50.0 ± 11.6
Anaesthetic and metabolite	Ketamine	11.8 ± 2.7	75.4 ± 11.3	166.2 ± 65.6	0.4 ± 0.2	2.8 ± 0.5
	Norketamine	<MQL	5.7 ± 2.8	12.2 ± 7.0	<MQL	0.8 ± 0.2
Antidepressants and metabolites	Venlafaxine	85.5 ± 13.0	509.5 ± 135.8	462.4 ± 137.3	8.6 ± 2.0	126.3 ± 26.0
	Desmethylvenlafaxine	229.2 ± 23.9	981.0 ± 128.6	733.9 ± 129.3	2.9 ± 1.1	22.4 ± 1.8
	Fluoxetine	1.2 ± 0.1	46.2 ± 4.0	53.2 ± 7.1	43.7 ± 32.1	193.7 ± 33.2
	Norfluoxetine	<MQL	<MQL	31.8 ± 6.5	9.6 ± 2.2	91.4 ± 26.5
	Sertraline	<MQL	13.1 ± 1.7	46.3 ± 7.0	114.1 ± 34.8	565.2 ± 71.3
	Mirtazapine	4.2 ± 1.6	41.3 ± 2.4	78.2 ± 13.1	6.9 ± 2.3	69.1 ± 8.7
	Citalopram	<MQL	321.9 ± 19.9	557.5 ± 109.9	87.5 ± 25.7	782.9 ± 75.2
	Desmethylcitalopram	12.0 ± 4.5	119.7 ± 10.7	216.2 ± 59.1	31.7 ± 8.8	295.7 ± 62.6
	Paroxetine	<MQL	<MQL	N/A	1.8 ± N/A	3.2 ± 2.9
	Duloxetine	<MQL	<MQL	N/A	<MQL	17.5 ± 5.8
	Amitriptyline	10.3 ± 3.2	53.3 ± 6.9	159.4 ± 25.2	115.6 ± 26.3	471.9 ± 44.1
	Nortriptyline	7.8 ± 4.0	30.6 ± 3.0	9.6 ± 4.2	9.9 ± 3.0	63.2 ± 15.2
	Norsertraline	N/A	N/A	157.8 ± N/A	133.8 ± 37.8	N/A
Anti-epileptic	Carbamazepine	160.4 ± 16.8	626.8 ± 60.5	521.2 ± 115.3	7.2 ± 2.6	118.7 ± 11.5
	Carbamazepine-10,11-epoxide	30.9 ± 4.2	138.2 ± 33.3	98.6 ± 29.9	N/A	N/A
	10,11-Dihydro-10-hydroxycarbamazepine	5.7 ± 2.1	43.2 ± 13.1	145.1 ± 55.1	<MQL	<MQL
Calcium channel blocker	Diltiazem	4.4 ± 2.0	59.5 ± 10.3	246.2 ± 55.5	N/A	N/A
	Verapamil	<MQL	<MQL	<MQL	5.8 ± 2.5	51.1 ± 6.1
Hypnotic	Temazepam	<MQL	21.5 ± 13.9	11.1 ± 7.4	<MQL	<MQL
	Oxazepam	2.0 ± 0.6	17.3 ± 4.1	16.6 ± 3.9	N/A	<MQL
	Diazepam	6.2 ± 0.8	11.6 ± 1.2	<MQL	0.9 ± 0.2	4.2 ± 0.4
Antipsychotic	Quetiapine	<MQL	<MQL	63.0 ± 31.4	2.9 ± 1.6	17.3 ± 4.7
	Risperidone	1.2 ± 1.4	2.6 ± 0.8	1.0 ± N/A	0.1 ± 0.1	<MQL
Dementia	Donepezil	<MQL	<MQL	N/A	0.9 ± 0.3	8.3 ± 0.2
	Memantine	<MQL	<MQL	<MQL	<MQL	<MQL
Human indicators	Creatinine	<MQL	<MQL	1,341,104.8 ± 314,821.7	N/A	N/A
	Nicotine	35.9 ± 16.1	183.9 ± 36.8	3461.2 ± 688.7	49.4 ± 26.3	227.1 ± 42.7
	Caffeine	426.5 ± 91.2	2938.9 ± 401.5	118,596.4 ± 24,610.9	N/A	N/A
	Cotinine	37.8 ± 5.7	214.5 ± 28.6	3028.3 ± 729.6	9.9 ± 2.9	40.1 ± 4.9
	1,7-Dimethylxanthine	923.5 ± 208.7	8352.1 ± 1091.1	153,776.3 ± 36,949.3	N/A	N/A
Analgesics and metabolites	Morphine	<MQL	141.7 ± 18.0	1366.1 ± 276.4	10.1 ± 3.8	63.2 ± 43.3
	Dihydromorphine	<MQL	<MQL	133.6 ± 32.7	<MQL	<MQL
	Normorphine	<MQL	<MQL	186.3 ± 50.7	<MQL	<MQL
	Methadone	3.0 ± 0.8	22.1 ± 1.5	41.6 ± 7.1	1.6 ± 0.5	12.9 ± 1.8
	EDDP	10.0 ± 1.5	50.3 ± 3.0	64.8 ± 12.9	11.0 ± 5.1	38.3 ± 22.3
	Codeine	85.6 ± 18.3	577.9 ± 58.6	2570.4 ± 555.0	23.3 ± 5.9	45.0 ± 42.5
	Norcodeine	<MQL	<MQL	181.0 ± 30.9	<MQL	<MQL
	Dihydrocodeine	22.1 ± 4.6	154.0 ± 19.3	445.2 ± 119.4	3.4 ± 0.9	24.8 ± 3.8
	Tramadol	321.7 ± 27.7	1273.7 ± 166.3	1247.5 ± 308.1	3.7 ± 1.0	34.5 ± 7.7
	*N*-Desmethyltramadol	255.3 ± 24.7	975.5 ± 118.3	1076.5 ± 355.9	1.5 ± 0.5	17.1 ± 5.6
	*O*-Desmethyltramadol	297.0 ± 32.9	1300.4 ± 200.0	861.6 ± 133.9	N/A	N/A
Stimulants and metabolites	Amphetamine	<MQL	<MQL	478.8 ± 135.7	<MQL	<MQL
	Methamphetamine	<MQL	9.1 ± 0.8	13.9 ± 3.7	<MQL	<MQL
	MDMA	4.3 ± 1.9	59.4 ± 33.3	183.2 ± 127.7	1.4 ± 0.9	6.1 ± 1.0
	MDA	10.0 ± 0.9	30.1 ± 7.6	45.5 ± N/A	N/A	N/A
	Cocaine	4.9 ± 1.6	47.1 ± 11.7	754.6 ± 295.3	6.9 ± 3.2	<MQL
	Benzoylecgonine	45.5 ± 9.9	291.2 ± 95.7	2078.4 ± 925.3	1.9 ± 1.1	<MQL
	Anhydroecgonine methyl ester	<MQL	<MQL	<MQL	N/A	N/A
	Cocaethylene	<MQL	3.6 ± 1.1	29.3 ± 18.5	0.4 ± 0.3	<MQL
	Mephedrone	<MQL	<MQL	<MQL	<MQL	<MQL
	MDPV	<MQL	<MQL	<MQL	<MQL	<MQL
Opioid and metabolite	Heroin	<MQL	<MQL	<MQL	<MQL	<MQL
	6-Acetylmorphine	<MQL	<MQL	<MQL	N/A	N/A
Pesticides, fungicides and herbicides	Thiamethoxam	<MQL	<MQL	<MQL	<MQL	<MQL
	Imidacloprid	53.0 ± 18.5	346.6 ± 133.7	339.2 ± 178.8	<MQL	N/A
	Clothianidin	<MQL	<MQL	<MQL	1.8 ± 0.8	N/A
	Metazachlor	4.5 ± N/A	<MQL	<MQL	4.2 ± 4.1	<MQL
	Terbuthylazine	<MQL	<MQL	<MQL	<MQL	<MQL
	Methiocarb	<MQL	3.0 ± N/A	3.7 ± 0.6	0.8 ± 0.6	1.7 ± 0.0
	Dichlofluanid	N/A	N/A	N/A	N/A	N/A
	Flufenacet	24.0 ± 2.4	61.8 ± 3.5	57.1 ± 12.9	4.1 ± 0.7	14.7 ± 0.5
	Oxadiazon	16.9 ± 3.4	29.8 ± 3.4	<MQL	9.3 ± 0.4	<MQL
	Chlorpyrifos	<MQL	<MQL	N/A	N/A	93.8 ± N/A
	Triallate	<MQL	<MQL	<MQL	N/A	N/A
Veterinary pharmaceuticals	Tylosin	<MQL	<MQL	<MQL	N/A	N/A
	Sulfapyridine	128.7 ± 19.6	576.0 ± 121.2	1339.5 ± 330.3	N/A	N/A
	Sarafloxacin	<MQL	<MQL	N/A	N/A	N/A
	Ceftiofur	31.2 ± N/A	216.1 ± 77.2	451.8 ± 129.2	N/A	N/A
	Diazinon	6.1 ± 0.3	12.0 ± 0.3	17.5 ± 0.4	11.9 ± 3.9	15.2 ± 3.6

Of further interest is the presence of veterinary pharmaceuticals in wastewater, and the presence of these can be justified by considering the number of household pets and the possible disposal route of pet waste down the lavatory. However, it is interesting to note that sulfapyridine (1339.5 ± 330.3 ng L^−1^) and ceftiofur (451.8 ± 129.2 ng L^−1^) are usually reserved for the use with individual pigs and cows. These levels suggest potential herd applications, incorrect disposal or unknown contribution of livestock wastewater to this WwTW. Further investigation is needed to determine the source and persistence of these levels.

For the solid portion of influent (SPM), 64% of the 98 analytes quantifiable with this method were found. Only a small fraction of the total concentration can be found in the SPM, as most CECs, particularly pharmaceuticals, prefer to partition to the aqueous phase. For example, only a fraction of the total concentration (6.4%) of bisphenol A (3.64 log *K*_ow_) appears in SPM. However, in this case, the concentrations are so high in influent that this results in bisphenol A contributing a large portion of the total measurable concentration of CECs. Concentrations range from an average concentration of 0.1 ng L^−1^ to 1383 ng L^−1^ in SPM (converted to ng L^−1^ for simple comparison to influent concentrations). Ketoconazole prefers to partition to the solid phase with 31% higher average concentration present in SPM. This is not surprising when considering its log *K*_ow_ of 4.45. The antidepressants fluoxetine (4.65 log *K*_ow_) and amitriptyline (4.95 log *K*_ow_) partition partially with SPM concentrations at 82% and 73% to that in influent. The results for fluoxetine are in line with the results published by Baker and Kasprzyk-Hordern [47]. However, sorption of amitriptyline is far higher in this study. This to be expected, as influent is highly variable, and many factors can affect sorption to solids. Therefore, it is important to analyse both the liquid and solid compartments of this matrix.

There are fewer CECs in effluent than in influent. Of the 137 analytes quantifiable in effluent, 62% were found at the WwTW. Generally, these results show lower concentrations after treatment. However, imatinib, *O*-desmethyltramadol, carbamazepine and its epoxide metabolite and venlafaxine and its metabolite increase in effluent. For metabolites, this may be due to degradation/metabolism of the parent compound during treatment. For the parent compounds, this may be due to the undetected presence of conjugated metabolites in influent transforming back to the parent compound. This phenomenon requires more detailed investigation. Comparison of the influent and effluent concentrations can provide data on treatment efficiencies. Trickling filters are employed at the WwTW for the treatment of influent. The results show poor removal of imidacloprid, tramadol, *N*-desmethyltramadol, bicalutamide, ranitidine, cetirizine and fexofenadine. Acetaminophen, on the other hand, shows high removal of 99%, similar to what is often seen in the literature for conventional activated sludge treatment [11, 48]. Caffeine, its metabolite 1,7-dimethylxanthine and metformin are highly removed but still at high concentrations of 2.9 ± 0.4 μg L^−1^, 8.4 ± 1.1 μg L^−1^ and 14.1 ± 0.9 μg L^−1^, respectively.

In the surface waters downstream of the WwTW, 51% of the 138 analytes that can be quantified are in this matrix. Of particular noteworthiness is the antidiabetic metformin, which is found at levels > 3000 ng L^−1^. Metformin has very high usage, and after administration, 100% of the dose is excreted unchanged [49]. Caldwell et al. [50] calculated the PNEC of metformin to be 1 mg L^−1^, based on critical evaluation of previously published work. This suggests that despite the high environmental levels, metformin is currently of low risk to this catchment. Within the literature, many compounds can be found to have different PNECs due to a lack of consistency in (a) assessment factors used, (b) limited sources or databases used in studying ecotoxicity data and (c) varying criteria in accepting ecotoxicity study results. This has highlighted the need to harmonise these methods to ensure PNECs are calculated consistently, to provide comparable comparisons between studies and to be clearer of the risks CECs pose to the environment.

Regarding digested solids, the concentrations are in ng g^−1^ of solid material, which cannot be directly compared to the concentrations of the other matrices, as it is a combination of sludge from various parts of the wastewater treatment process. However, it is an important consideration as a source of CECs in the environment, due to subsequent direct application to the land in agricultural practices. Of the 96 analytes that can be quantified with this method, 55% were found at this site. Antidepressants are high in concentration, with average concentrations of five of the analytes between 126.3 and 782.9 ng g^−1^. Of the more industrial CECs, methylparaben and bisphenol A are present in solids at high levels. In particular, bisphenol A has been quantified at levels exceeding 4000 ng g^−1^ at this site. Gemfibrozil, an antihyperlipidaemic, although not found in other matrices at this site, was quantified in digested solids at a concentration of 849.6 ± 183.8 ng g^−1^. This suggests accumulation of gemfibrozil in other sections of the WwTW that were not analysed. Due to the low MQL, the lack of incoming concentration during the sampling period suggests occasional loads high in gemfibrozil before the study.

## Conclusions

This work presents a validated multi-residue method for the analysis of 195 compounds in five matrices (3 liquid and 2 solid). These CECs cover a variety classes, both veterinary and human pharmaceuticals, industrial chemical, personal care products and pesticides. Application of the method to environmental matrices has shown that the method is appropriate for assessing treatment efficiency, partitioning to solids, and environmental concentrations. Discussion of the results has identified several key areas regarding environmental risk assessment, e.g. PNECs that need to be addressed; however, that is outside the scope of this paper. The achieved MDL and MQL concentrations appear low enough to be used to assess the environmental risk of these CECs. The results show a need for analysing both the liquid and solid phases within a WwTW; however, it also indicates a need to monitor all outgoing treated waste materials, e.g. effluent and digested sludge. This was due to the appearance of gemfibrozil, which was not present at quantifiable levels in any other matrix at this site. Overall, this method is suitable to be used in catchment-based exposure-driven studies to further increase knowledge of the contribution of CECs by WwTWs to the environment and the risk they pose.

## Electronic supplementary material

ESM 1 (PDF 2689 kb)
