# Supplementary material for: Multi-residue ultra-performance liquid chromatography coupled with tandem mass spectrometry method for comprehensive multi-class anthropogenic compounds of emerging concern analysis in a catchment-based exposure-driven study
Source: Anal Bioanal Chem. 2019 Sep 7;411(27):7061–86. doi: 10.1007/s00216-019-02091-8 (PMC6838033; doi:10.1007/s00216-019-02091-8)
Supplement: Supplementary file 1 — (PDF 2689 kb) [file 216_2019_2091_MOESM1_ESM.pdf]

Analytical and Bioanalytical Chemistry

Electronic Supplementary Material

Multi-residue ultra-performance liquid chromatography coupled with tandem mass spectrometry method for comprehensive multi-class anthropogenic compounds of emerging concern analysis in a catchment-based exposure-driven study

Kathryn Proctor, Bruce Petrie, Ruth Barden, Tom Arnot, Barbara Kasprzyk-Hordern

Contents	Page
Table S1 *General and chemical information (Class, Chemical, CAS No. Mol Formula, Molecular Weight, Water sol. Log Kow, Log Koc, Log Dow.)*	2
Table S2 *MS/MS detection parameters for analytes of interest sorted by class, includes analytes from ESI –ve and ESI +ve methods*	8
Table S3 *MS/MS detection parameters for internal standards sorted by analogue analyte class, includes internal standards from ESI –ve and ESI +ve methods*	13
Figure S1 *Chromatograms of all analytes in spiked influent sample from the WwTW using methods: A-ESI+, B-ESI+ and C-ESI-*	14
Table S4 *Solid sample weights*	15
Table S5 *Suspended solids weights (g L^{-1})*	15
Table S6 *Adsorbance*	16
Table S7 *Instrument parameters (LC MSMS): Intra- and Inter-day precision and accuracy*	20
Table S8 *Liquid matrix absolute and corrected recoveries*	24
Table S9 *Solid matrix absolute and corrected recoveries*	30
Figure S2 *Absolute recoveries for all matrices, error bars indicate the range of standard deviation*	34
Table S10 *Matrix suppression*	35
Table S11 *Surface water data*	39
Table S12 *Effluent data*	45
Table S13 *Influent data*	51
Table S14 *Solid particulate matter data*	57
Table S15 *Digested solids data*	63

Table S1 General and chemical information (Class, Chemical, CAS No. Mol Formula, Molecular Weight, Water sol. Log Kow, Log Koc, Log Dow.)

Compound	CAS no.	Molecular Formula	Molecular Weight	Water Solubility (mg/L)[i]	Log Kow[ii]	Log Koc[iii]	Log Dow[iv]	Henry's Law Constant (atm $m^3 mol^{-1}$)[v]	Vapour Pressure (Torr)[vi]	pKa (Most acidic)[Vii]	pKa (Most basic)[vii]	Supplier
Benzophenone-1	131-56-6	$C_{13}H_{10}O_3$	214.22	413	2.96	3.092	2.9	$2.65x10^{-11}$	$2.84x10^{-7}$	7.72±0.35	-	Sigma Aldrich
Benzophenone-2	131-55-5	$C_{13}H_{10}O_5$	246.22	399	2.78	3.326	2.35	$3.61x10^{-16}$	$6.69x10^{-12}$	6.98±0.35	-	Sigma Aldrich
Benzophenone-3	131-57-7	$C_{14}H_{12}O_3$	228.25	68.6	3.52	3.44	3.06	$1.50x10^{-8}$	$5.26x10^{-6}$	7.56±0.35	-	Sigma Aldrich
Benzophenone-4	4065-45-6	$C_{14}H_{12}O_6S$	308.31	$2.03x10^{4}$	0.37	1.548	-0.53	$7.06x10^{-15}$	-	-0.70±0.50	-	Sigma Aldrich
Methylparaben	99-76-3	$C_8H_8O_3$	152.15	$5.98x10^{3}$	2	2.11	1.63	$3.61x10^{-9}$	$5.55x10^{-3}$	8.31±0.13	-	Sigma Aldrich
Ethylparaben	120-47-8	$C_9H_{10}O_3$	166.18	$1.89x10^{3}$	2.49	2.393	1.99	$4.79x10^{-9}$	$7.59x10^{-4}$	8.31±0.13	-	Sigma Aldrich
Propylparaben	94-13-3	$C_{10}H_{12}O_3$	180.21	529	2.98	2.708	2.51	$4.25x10^{-12}$	$9.30x10^{-4}$	8.23±0.15	-	Sigma Aldrich
Butylparaben	94-26-8	$C_{11}H_{14}O_3$	194.23	159	3.47	3.001	2.96	$6.00x10^{-9}$	$3.56x10^{-4}$	8.22±0.15	-	Sigma Aldrich
Bisphenol A	80-05-7	$C_{15}H_{16}O_2$	228.29	173	3.64	3.095	4.04	$9.16x10^{-12}$	$5.34x10^{-7}$	10.29±0.10	-	Sigma Aldrich
E1	53-16-7	$C_{18}H_{22}O_2$	270.37	147	3.43	3.019	4.31	$3.80x10^{-10}$	$1.54x10^{-8}$	10.25±0.40	-	Sigma Aldrich
E2	50-28-2	$C_{18}H_{24}O_2$	272.39	82.0	3.94	2.899	3.74	$1.41x10^{-12}$	$9.82x10^{-9}$	10.27±0.60	-	Sigma Aldrich
EE2	57-63-6	$C_{20}H_{24}O_2$	296.41	116	4.12	2..710	3.9	$7.94x10^{-12}$	$3.74x10^{-9}$	10.24±0.60	-	Sigma Aldrich
Sulfasalazine	599-79-1	$C_{18}H_{14}N_4O_5S$	398.4	2.40	3.81	3.177	-1.76	$2.19x10^{-18}$	$5.95x10^{-20}$	2.70±0.10	0.90±0.10	Sigma Aldrich
Clarithromycin	81103-11-9	$C_{38}H_{69}NO_{13}$	747.97	0.30	3.18	1.371	2.31	$1.73x10^{-29}$	$5.06x10^{-30}$	13.08±0.70	8.16±0.70	Sigma Aldrich
Azithromycin	83905-01-5	$C_{38}H_{72}N_2O_{12}$	749	0.06	3.24	1.676	-2.48	$5.30x10^{-29}$	$2.51x10^{-31}$	13.28±0.70	8.59±0.70	Sigma Aldrich
Trimethoprim	738-70-5	$C_{14}H_{18}N_4O_3$	290.32	$2.33x10^{3}$	0.73	1.896	1.13	$2.39x10^{-14}$	$3.74x10^{-11}$	-	7.04±0.10	Sigma Aldrich
Sulfamethoxazole	723-46-6	$C_{10}H_{11}N_3O_3S$	253.28	$3.94\ x10^{3}$	0.48	1.536	-0.03	$9.56x10^{-13}$	$1.87x10^{-9}$	5.81±0.50	1.39±0.10	Sigma Aldrich
Triclosan	3380-34-5	$C_{12}H_7Cl_3O_2$	289.55	4.60	4.66	3.925	4.76	$2.13x10^{-8}$	$3.26x10^{-5}$	7.80±0.35	-	Sigma Aldrich
Amoxicillin	26787-78-0	$C_{16}H_{19}N_3O_5S$	365.4	$3.43\ x10^{3}$	0.97	0.709	-2.36	$2.49x10^{-21}$	$3.39x10^{-21}$	2.44±0.50	7.14±0.10	Sigma Aldrich
Metronidazole	443-48-1	$C_6H_9N_3O_3$	171.16	$2.57x10^{4}$	0	1.12	-0.46	$1.69x10^{-11}$	$2.67x10^{-7}$	14.44±0.10	2.58±0.34	Sigma Aldrich
Sulfadiazine	68-35-9	$C_{10}H_{10}N_4O_2S$	250.28	$2.81x10^{4}$	-0.34	1.392	-0.1	$1.58x10^{-10}$	$1.28x10^{-10}$	6.81±0.10	1.64±0.10	Sigma Aldrich (VETRANAL)
Cefalexin	15686-71-2	$C_{16}H_{17}N_3O_4S$	347.39	$1.79\ x10^{3}$	0.4	0.421	-2.19	$2.77x10^{-17}$	$3.27x10^{-22}$	3.12±0.50	6.84±0.10	Sigma Aldrich
Ofloxacin	82419-36-1	$C_{18}H_{20}FN_3O_4$	361.37	$2.83x10^{4}$	-0.2	-0.004	-0.39	$4.98x10^{-20}$	$6.70x10^{-14}$	5.19±0.40	7.37±0.42	Sigma Aldrich
Ciprofloxacin	85721-33-1	$C_{17}H_{18}FN_3O_3$	331.34	$1.15\ x10^{4}$	0	-0.004	-0.94	$5.09x10^{-19}$	$2.24x10^{-14}$	6.43±0.41	8.68±0.10	Sigma Aldrich (Fluka)
Tetracycline	60-54-8	$C_{22}H_{24}N_2O_8$	444.43	$3.88x10^{3}$	-1.33	-0.128	-2.35	$4.66x10^{-24}$	$2.40x10^{-26}$	4.50±1.00	11.02±0.70	Sigma Aldrich

Compound	CAS no.	Molecular Formula	Molecular Weight	Water Solubility (mg/L)[i]	Log Kow[ii]	Log Koc[iii]	Log Dow[iv]	Henry's Law Constant (atm m^3mol^{-1})[v]	Vapour Pressure (Torr)[vi]	pKa (Most acidic)[Vii]	pKa (Most basic)[vii]	Supplier
Danofloxacin	112398-08-0	$C_{19}H_{20}FN_3O_3$	357.38	-	-	-	-	-	$8.41x10^{-14}$	6.43±0.41	9.00±0.20	Sigma Aldrich
Oxytetracycline	79-57-2	$C_{22}H_{24}N_2O_9$	460.43	$1.40x10^{3}$	-2.87	0.093	-3.35	$1.70x10^{-25}$	$6.27x10^{-30}$	4.50±1.00	10.80±0.70	Sigma Aldrich
Chloramphenicol	56-75-7	$C_{11}H_{12}Cl_2N_2O_5$	323.13	389	1.14	0.926	-2.19	$2.29x10^{-18}$	$1.63x10^{-17}$	11.03±0.46	-1.73±0.70	Sigma Aldrich
Penicillin G	61-33-6	$C_{16}H_{18}N_2O_4S$	334.39	210	1.85	1.095	-2.45	$1.16x10^{-14}$	$1.69x10^{-18}$	2.45±0.50	-1.32±0.60	Sigma Aldrich
Penicillin V	87-08-1	$C_{16}H_{18}N_2O_5S$	350.39	101	1.87	1.295	-2.77	$4.42x10^{-15}$	$1.69x10^{-19}$	2.44±0.50	-1.68±0.60	Sigma Aldrich
Erythromycin	114-07-8	$C_{37}H_{67}NO_{13}$	733.93	0.52	2.48	1.406	1.66	$5.42x10^{-29}$	$4.94x10^{-31}$	13.09±0.70	8.16±0.70	Sigma Aldrich
Prulifloxacin	123447-62-1	$C_{21}H_{20}FN_3O_6S$	461.46	-	-	-	-	-	$6.62x10^{-17}$	5.85±0.40	7.66±0.10	Sigma Aldrich
Norfloxacin	70458-96-7	$C_{16}H_{18}FN_3O_3$	319.33	$1.78x10^{5}$	-0.31	-0.392	-1.05	$8.70x10^{-19}$	$3.45x10^{-13}$	0.16±0.20	8.68±0.10	Sigma Aldrich
Griseofulvin	126-07-8	$C_{17}H_{17}ClO_6$	352.77	82.0	1.92	2.543	2.17	$2.35x10^{-11}$	$5.07x10^{-13}$	-	-	Sigma Aldrich
Ketoconazole	65277-42-1	$C_{26}H_{28}Cl_2N_4O_4$	531.43	0.09	4.45	3.492	4.14	$5.59x10^{-20}$	$1.39x10^{-22}$	-	6.88±0.12	Sigma Aldrich
Valsartan	137862-53-4	$C_{24}H_{29}N_5O_3$	435.53	1.41	3.65	2.2	1.15	$1.82x10^{-18}$	$1.06x10^{-19}$	3.56±0.10	0.60±0.10	Sigma Aldrich
Irbesartan	138402-11-6	$C_{25}H_{28}N_6O$	428.53	-	-	-	4.97	-	$1.05x10^{-16}$	4.16±0.10	2.60+0.20	LGC
Lisinopril	76547-98-3	$C_{21}H_{31}N_3O_5$	405.5	8.60	-0.94	-0.516	-3.23	$1.89x10^{-22}$	$1.14x10^{-18}$	2.18±0.10	10.50±0.10	LGC
Ketoprofen	22071-15-4	$C_{16}H_{14}O_3$	254.29	120	3	2.077	0.08	$2.12x10^{-11}$	$3.32x10^{-8}$	4.23±0.10	-	Sigma Aldrich
Ibuprofen	15687-27-1	$C_{13}H_{18}O_2$	206.29	41.1	3.79	2.352	1.25	$1.52x10^{-7}$	$1.39x10^{-4}$	4.41±0.10	-	Sigma Aldrich
Naproxen	22204-53-1	$C_{14}H_{14}O_3$	230.27	145	3.1	1.971	-0.54	$3.39x10^{-10}$	$3.01x10^{-7}$	4.84±0.30	-	TRC
Diclofenac	15307-86-5	$C_{14}H_{11}Cl_2NO_2$	296.15	4.50	4.02	2.607	0.73	$4.73x10^{-12}$	$1.59x10^{-7}$	4.18±0.10	-2.26±0.50	Sigma Aldrich
Acetaminophen	103-90-2	$C_8H_9NO_2$	151.17	$3.04x10^{4}$	0.27	1.321	0.9	$6.42x10^{-13}$	$1.43x10^{-6}$	9.86±0.13	1.72±0.50	Sigma Aldrich
Bezafibrate	41859-67-0	$C_{19}H_{20}ClNO_4$	361.83	1.20	4.25	2.311	0.46	$2.12x10^{-15}$	$6.29x10^{14}$	3.29±0.10	-2.06±0.70	Sigma Aldrich
Atorvastatin	134523-00-5	$C_{33}H_{35}FN_2O_5$	558.66	$1.12x10^{-3}$	6.36	2.6	1.86	$2.41x10^{-23}$	$6.84x10^{-22}$	4.29±0.10	0.38±0.50	LGC
Gemfibrozil	25812-30-0	$C_{15}H_{22}O_3$	250.33	4.96	4.77	2.85	0.86	$1.19x10^{-8}$	$6.13x10^{-7}$	4.75±0.45	-	Sigma Aldrich
Candesartan Cilexetil	145040-37-5	$C_{33}H_{34}N_6O_6$	610.66	-	-	-	1.19	-	$1.11x10^{-28}$	4.16±0.10	3.94±0.10	Sigma Aldrich
Fexofenadine	83799-24-0	$C_{32}H_{39}NO_4$	501.67	0.02	2.81	0.822	2.93	$1.19x10^{-18}$	$2.08x10^{-20}$	4.43±0.10	9.42±0.10	LGC
Cetirizine	83881-51-0	$C_{21}H_{25}ClN_2O_3$	388.9	1.10	-0.61	0.875	0.69	$4.19x10^{-17}$	$1.39x10^{-12}$	3.46±0.10	6.71±0.10	LGC
Sildenafil	139755-83-2	$C_{22}H_{30}N_6O_4S$	474.58	11.3	2.3	2.661	1.13	$7.22x10^{-21}$	-	10.05±0.20	6.03±0.42	Sigma Aldrich (Cerilliant)
Metformin	657-24-9	$C_4H_{11}N_5$	129.17	$1.00x10^{6}$	-2.64	-0.666	-6.45	$7.64x10^{-16}$	1.33	-	12.27±0.10	Sigma Aldrich
Gliclazide	21187-98-4	$C_{15}H_{21}N_3O_3S$	323.41	138	2.12	2.205	0.79	$7.95x10^{-13}$	-	6.07±0.10	3.89±0.20	LGC

Compound	CAS no.	Molecular Formula	Molecular Weight	Water Solubility (mg/L)[i]	Log Kow[ii]	Log Koc[iii]	Log Dow[iv]	Henry's Law Constant (atm m^3mol^{-1})[v]	Vapour Pressure (Torr)[vi]	pKa (Most acidic)[Vii]	pKa (Most basic)[vii]	Supplier
Sitagliptin	486460-32-6	$C_{16}H_{15}F_6N_5O$	407.31	179	1.39	1.698	-0.51	$2.35x10^{-15}$	$2.59x10^{-11}$	7.2±0.10	-	TRC
Pholcodine	509-67-1	$C_{23}H_{30}N_2O_4$	398.51	$1.01x10^4$	0.59	0.731	-0.48	$3.42x10^{-19}$	$3.44x10^{-14}$	13.40±0.20	8.22±0.40	Sigma Aldrich
Atenolol	29122-68-7	$C_{14}H_{22}N_2O_3$	266.34	685	-0.03	0.611	-1.71	$1.37x10^{-18}$	$3.82x10^{-11}$	13.88±0.20	9.43±0.10	Sigma Aldrich
Metoprolol	51384-51-1	$C_{15}H_{25}NO_3$	267.37	$4.77x10^3$	1.69	1.475	-0.38	$1.40x10^{-13}$	$4.52x10^{-7}$	13.89±0.20	9.43±0.10	Sigma Aldrich
Propranolol	525-66-6	$C_{16}H_{21}NO_2$	259.35	228	2.6	2.451	0.45	$7.98x10^{-13}$	$2.48x10^{-8}$	13.84±0.20	9.50±0.30	Sigma Aldrich
Bisoprolol	66722-44-9	$C_{18}H_{31}NO_4$	325.44	$2.24\ x10^3$	1.84	1.379	0.06	$2.89x10^{-15}$	$1.06x10^{-8}$	13.86±0.20	9.42±0.10	Sigma Aldrich
Ranitidine	66357-35-5	$C_{13}H_{22}N_4O_3S$	314.41	$2.47x10^4$	0.29	1.141	-1.56	$3.42x10^{-15}$	$7.66x10^{-8}$	-	8.35±0.28	Sigma Aldrich
Cimetidine	51481-61-9	$C_{10}H_{16}N_6S$	252.34	$1.05x10^4$	0.57	1.85	-0.3	$9.55x10^{-16}$	$3.13x10^{-9}$	14.13±0.10	7.07±0.61	Sigma Aldrich
Iopromide	73334-07-3	$C_{18}H_{24}I_3N_3O_8$	791.12	23.8	-2.49	-1.672	-0.44	$1.00x10^{-28}$	$5.00x10^{-30}$	10.62±0.70	-2.60±0.70	LGC
Buprenorphine	52485-79-7	$C_{29}H_{41}NO_4$	467.64	0.66	4.9	3.335	1.37	$7.78x10^{-18}$	-	9.47±0.60	8.31±0.60	Sigma Aldrich
Ephedrine/pseudoephedrine	299-42-3	$C_{10}H_{15}NO$	165.24	$7.15x10^4$	0.68	1.095	-1.13	$8.65x10^{-11}$	$8.65x10^{-3}$	13.96±0.20	9.38±0.10	Sigma Aldrich
Norephedrine	492-39-7	$C_9H_{13}NO$	151.21	$1.49x10^5$	0.22	0.951	-1.4	$3.94x10^{-11}$	$1.10x10^{-3}$	12.07±0.45	8.47±0.10	Sigma Aldrich
Azathioprine	446-86-6	$C_9H_7N_7O_2S$	277.26	272	-0.09	2.395	1.21	$2.64x10^{-15}$	$5.94x10^{-11}$	-	7.47±0.20	Sigma Aldrich
Methotrexate	59-05-2	$C_{20}H_{22}N_8O_5$	454.45	$2.60x10^3$	-1.28	-0.387	-7.06	$1.54x10^{-31}$	-	3.47±0.10	5.56±0.10	LGC
Ifosfamide	3778-73-2	$C_7H_{15}Cl_2N_2O_2P$	261.09	$3.78x10^3$	0.97	1.439	0.1	$1.36x10^{-11}$	$1.15x10^{-4}$	-	1.44±0.20	Sigma Aldrich
Tamoxifen	10540-29-1	$C_{26}H_{29}NO$	371.53	0.20	6.3	4.4	5.07	$4.49x10^{-10}$	$1.85x10^{-9}$	-	8.69±0.28	LGC
Imatinib	152459-95-5	$C_{29}H_{31}N_7O$	493.6	-	-	-	2.04	-	-	13.28±0.70	7.55±0.10	Sigma Aldrich
Capecitabine	154361-50-9	$C_{15}H_{22}FN_3O_6$	359.35	$1.82x10^3$	0.56	0.173	0.74	$2.92x10^{-19}$	-	5.41±0.40	1.75±0.20	Sigma Aldrich
Bicalutamide	90357-06-5	$C_{18}H_{14}F_4N_2O_4S$	430.37	11.8	2.3	2.177	2.71	$2.82x10^{-15}$	$8.47x10^{-18}$	11.49±0.29	-3.75±0.50	Sigma Aldrich
Ketamine	6740-88-1	$C_{13}H_{16}ClNO$	237.73	$3.87x10^3$	3.12	2.283	3.18	$1.38x10^{-8}$	$1.76x10^{-5}$	-	6.46±0.20	Sigma Aldrich
Norketamine	35211-10-0	$C_{15}H_{11}ClN_2O$	233.7	-	-	-	2.71	$1.78x10^{-10}$	$1.26x10^{-5}$	-	6.25±0.20	Sigma Aldrich
Venlafaxine	93413-69-5	$C_{17}H_{27}N_1O_2$	277.41	267	3.28	2.318	1.32	$2.87x10^{-11}$	$4.92x10^{-7}$	14.84±0.20	9.26±0.28	Sigma Aldrich
Desmethylvenlafaxine	93413-62-8	$C_{16}H_{25}NO_2$	263.38	-	-	-	1.17	-	$3.03x10^{-7}$	10.04±0.26	9.33±0.28	Sigma Aldrich
Fluoxetine	54910-89-3	$C_{17}H_{18}F_3NO$	309.33	60.3	4.65	3.05	1.92	$8.90x10^{-8}$	$1.88x10^{-6}$	-	10.05±0.10	LGC (Cerilliant)
Norfluoxetine	83891-03-6	$C_{16}H_{16}F_3NO$	295.3	-	-	-	1.54	-	$5.21x10^{-6}$	-	9.05±0.13	LGC (Cerilliant)
Sertraline	79617-96-2	$C_{17}H_{17}Cl_2N$	306.24	3.50	5.29	3.808	3.11	$5.10x10^{-8}$	$3.85x10^{-7}$	-	9.47±0.40	LGC
Mirtazapine	85650-52-8	$C_{17}H_{19}N_3$	265.35	-	-	-	3.15	-	$1.11x10^{-7}$	-	8.10±0.20	Sigma Aldrich

Compound	CAS no.	Molecular Formula	Molecular Weight	Water Solubility (mg/L)[i]	Log Kow[ii]	Log Koc[iii]	Log Dow[iv]	Henry's Law Constant (atm m^3mol^{-1})[v]	Vapour Pressure (Torr)[vi]	pKa (Most acidic)[Vii]	pKa (Most basic)[vii]	Supplier
Citalopram	59729-33-8	$C_{20}H_{21}FN_2O$	324.4	31.1	3.74	3.23	1.5	$2.69x10^{-11}$	$1.53x10^{-7}$	-	9.57±0.28	Sigma Aldrich
Desmethylcitalopram	62498-67-3	$C_{19}H_{19}FN_2O$	310.37	-	-	-	0.14	-	$1.40x10^{-7}$	-	10.50±0.10	TRC
Paroxetine	61869-08-7	$C_{19}H_{20}FNO_3$	329.37	35.3	3.95	3.088	0.66	$1.78x10^{-12}$	$2.39x10^{-8}$	9.68±0.10	-	Sigma Aldrich
Duloxetine	116539-59-4	$C_{18}H_{19}NOS$	297.41	13.0	4.68	3.526	1.77	$5.43x10^{-10}$	$7.23x10^{-9}$	10.02±0.10	-	LGC
Amitriptyline	50-48-6	$C_{20}H_{23}N$	277.4	0.82	4.95	3.581	2.57	$6.85x10^{-8}$	$1.50x10^{-6}$	9.18±0.28	-	Sigma Aldrich
Nortriptyline	72-69-5	$C_{19}H_{21}N$	263.38	2.22	4.74	3.376	1.64	$3.12x10^{-8}$	$1.02x10^{-6}$	10.00±0.10	-	Sigma Aldrich
Norsertraline	87857-41-8	$C_{16}H_{15}Cl_2N$	292.2	-	-	-	1.68	-	$6.41x10^{-7}$	9.13±0.40	-	LGC
Carbamazepine	298-46-4	$C_{15}H_{12}N_2O$	236.28	17.7	2.25	2.227	2.77	$1.08x10^{-10}$	$5.78x10^{-7}$	13.94±0.20	-0.49±0.20	Sigma Aldrich
Carbamazepine 10,11-epoxide	36507-30-9	$C_{15}H_{12}N_2O_2$	252.27	-	-	-	1.97	-	$2.69x10^{-6}$	13.91±0.20	-0.50±0.20	LGC
10,11-Dihydro -10-hydroxycarbamazepine	29331-92-8	$C_{15}H_{14}N_2O_2$	254.28	-	-	-	1.73	-	$3.33x10^{-8}$	13.75±0.20	-0.53±0.40	LGC
Diltiazem	42399-41-7	$C_{22}H_{26}N_2O_4S$	414.52	12.3	2.79	2.296	1.97	$8.61x10^{-17}$	$4.27x10^{-14}$	-	8.94±0.28	Sigma Aldrich
Verapamil	52-53-9	$C_{27}H_{38}N_2O_4$	454.6	4.47	4.8	3.46	2.42	$8.79x10^{-15}$	$1.01x10^{-13}$	8.97±0.50	-	LGC
Temazepam	846-50-4	$C_{16}H_{13}ClN_2O_2$	300.75	164	2.15	1.678	2.79	$1.13x10^{-8}$	$6.33x10^{-13}$	11.66±0.40	1.58±0.50	Sigma Aldrich (Cerilliant)
Oxazepam	604-75-1	$C_{15}H_{11}ClN_2O_2$	286.71	179	2.32	1.727	2.92	$5.53x10^{-10}$	$4.40x10^{-11}$	10.94±0.70	1.17±0.50	Sigma Aldrich (Cerilliant)
Diazepam	439-14-5	$C_{16}H_{13}ClN_2O$	284.74	58.8	2.7	2.438	3.08	$3.64x10^{-9}$	$4.98x10^{-10}$	3.40±0.10	-	Sigma Aldrich (Cerilliant)
Quetiapine	111974-69-7	$C_{21}H_{25}N_3O_2S$	384.52	0.60	3.17	2.111	2.67	$7.45x10^{-18}$	$3.22x10^{-13}$	14.41±0.10	6.74±0.10	LGC
Risperidone	106266-06-2	$C_{23}H_{27}FN_4O_2$	410.48	2.76	3.49	2.743	0.86	$2.17x10^{-16}$	$4.14x10^{-13}$	8.07±0.10	-	LGC
Donepezil	120014-06-4	$C_{24}H_{29}NO_3$	379.49	2.93	4.86	3.855	0.71	$1.22x10^{-12}$	$3.11x10^{-11}$	8.84±0.10	-	LGC
Memantine	19982-08-2	$C_{12}H_{21}N$	179.3	894	3.34	2.382	-0.97	$1.47x10^{-5}$	$3.93x10^{-2}$	10.79±0.60	-	Sigma Aldrich
Creatinine	60-27-5	$C_4H_7N_3O$	113.12	$1.66x10^{5}$	-1.21	-0.39	-1.07	$2.42x10^{-12}$	0.31	-	6.89±0.20	Sigma Aldrich
Nicotine	54-11-5	$C_{10}H_{14}N_2$	162.24	$1.00x10^{6}$	1	1.683	-2.34	$3.00x10^{-9}$	$3.03x10^{-2}$	-	8.00±0.50	Sigma Aldrich
Caffeine	58-08-2	$C_8H_{10}N_4O_2$	194.19	$2.63x10^{3}$	0.16	0.98	-0.55	$3.58x10^{-11}$	$3.72x10^{-7}$	-	0.52±0.70	Sigma Aldrich
Cotinine	486-56-6	$C_{10}H_{12}N_2O$	176.22	$9.99x10^{5}$	0.34	1.093	0.21	$3.33x10^{-12}$	$4.21x10^{-4}$	-	4.72±0.12	Sigma Aldrich (Cerilliant)
1,7-dimethylxanthine	611-59-6	$C_7H_8N_4O_2$	180.17	$4.14x10^{3}$	-0.39	0.919	0.24	$1.75x10^{-12}$	-	8.50±0.50	0.21±0.70	Sigma Aldrich
Morphine	57-27-2	$C_{17}H_{19}NO_3$	285.35	$2.64x10^{4}$	0.72	1.163	-0.37	$1.33x10^{-16}$	$7.06x10^{-10}$	9.48±0.40	8.25±0.40	Sigma Aldrich (Cerilliant)

Compound	CAS no.	Molecular Formula	Molecular Weight	Water Solubility (mg/L)[i]	Log Kow[ii]	Log Koc[iii]	Log Dow[iv]	Henry's Law Constant (atm m^3mol^{-1})[v]	Vapour Pressure (Torr)[vi]	pKa (Most acidic)[Vii]	pKa (Most basic)[vii]	Supplier
Dihydromorphine	509-60-4	$C_{17}H_{21}NO_3$	287.36	2.38×10^{4}	0.93	1.185	-0.26	1.51×10^{-16}	7.65×10^{-10}	9.56±0.40	8.44±0.40	Sigma Aldrich (Cerilliant)
Normorphine	466-97-7	$C_{16}H_{17}NO_3$	271.32	2.56×10^{5}	0.5	0.599	-2.05	6.07×10^{-17}	2.99×10^{-10}	9.17±0.40	9.54±0.40	Sigma Aldrich (Cerilliant)
Methadone	76-99-3	$C_{21}H_{27}NO$	309.46	48.5	4.17	3.229	3.39	4.97×10^{-10}	2.20×10^{-7}	-	9.50±0.50	Sigma Aldrich (Cerilliant)
EDDP	30223-73-5	$C_{20}H_{23}N$	277.4	-	-	-	2.5	-	2.06×10^{-6}	-	7.71±0.60	LGC (Cerilliant)
Codeine	76-57-3	$C_{18}H_{21}NO_3$	299.37	1.22×10^{4}	1.28	1.218	-0.23	7.58×10^{-14}	2.47×10^{-9}	13.40±0.20	8.23±0.40	Sigma Aldrich
Norcodeine	467-15-2	$C_{17}H_{19}NO_3$	285.35	3.92×10^{4}	1.07	0.964	-2.28	3.45×10^{-14}	1.51×10^{-9}	13.34±0.20	9.28±0.40	Sigma Aldrich (Cerilliant)
Dihydrocodeine	125-28-0	$C_{18}H_{23}NO_3$	301.39	6.53×10^{3}	1.49	1.384	-0.11	8.61×10^{-14}	2.48×10^{-9}	14.22±0.20	8.43±0.40	Sigma Aldrich
Tramadol	27203-92-5	$C_{16}H_{25}NO_2$	263.38	1.15×10^{3}	3.01	1.959	0.72	1.54×10^{-11}	1.02×10^{-6}	14.47±0.40	9.61±0.28	Sigma Aldrich
N-desmethyltramadol	75377-45-6	$C_{15}H_{23}NO_2$	249.35	-	-	-	-0.27	-	9.16×10^{-6}	14.46±0.40	10.56±0.10	LGC
O-desmethyltramadol	144830-15-9(S,S)/ 144830-14-8 (R,R)	$C_{15}H_{23}NO_2$	249.35	-	-	-	0.38	-	3.14×10^{-7}	10.00±0.10	9.61±0.28	LGC
Amphetamine	300-62-9	$C_9H_{13}N_1$	135.21	2.80×10^{4}	1.76	1.877	-1.23	1.08×10^{-6}	0.31	-	9.94±0.10	LGC (Cerilliant)
Methamphetamine	537-46-2	$C_{10}H_{15}N$	149.24	1.33×10^{4}	2.22	2.027	-1.01	2.37×10^{-6}	0.15	-	10.38±0.10	LGC (Cerilliant)
MDMA	42542-10-9	$C_{11}H_{15}NO_2$	193.25	7.03×10^{3}	2.28	2.183	-1.38	2.75×10^{-9}	3.17×10^{-3}	-	10.32±0.10	LGC
MDA	101-77-9	$C_{13}H_{14}N_2$	198.27	1.96×10^{3}	2.18	1.761	-1.61	1.58×10^{-11}	1.52×10^{-6}	-	5.32±0.25	LGC (Cerilliant)
Cocaine	50-36-2	$C_{17}H_{21}NO_4$	303.36	1.30×10^{3}	2.17	2.001	0.92	4.24×10^{-11}	1.87×10^{-6}	-	8.97±0.60	LGC (Cerilliant)
Benzoylecgonine	519-09-5	$C_{16}H_{19}NO_4$	289.33	1.61×10^{3}	-1.32	-0.705	-0.6	1.03×10^{-13}	1.32×10^{-8}	3.35±0.40	10.83±0.40	Sigma Aldrich
Anhydroecgoninemethylester	43021-26-7	$C_{10}H_{15}NO_2$	181.23	-	-	-	0.21	-	2.19×10^{-2}	-	7.97±0.40	Sigma Aldrich (Cerilliant)
Cocaethylene	529-38-4	$C_{18}H_{23}NO_4$	317.38	-	-	-	1.35	-	6.80×10^{-7}	-	9.04±0.60	Sigma Aldrich (Cerilliant)
Mephedrone	1189805-46-6	$C_{11}H_{15}NO$	177.24	-	-	-	1.47	-	3.84×10^{-3}	-	7.41±0.10	Sigma Aldrich (Cerilliant)
MDPV	687603-66-3	$C_{16}H_{21}NO_3$	275.34	-	-	-	2.77	-	4.09×10^{-7}	-	8.41±0.20	Sigma Aldrich
Heroin	561-27-3	$C_{21}H_{23}NO_5$	369.42	2.15×10^{3}	1.8	1.658	-0.06	6.15×10^{-13}	7.38×10^{-10}	-	7.93±0.40	Sigma Aldrich (Cerilliant)
6-acetylmorphine	2784-73-8	$C_{19}H_{21}NO_4$	327.37	-	-	-	-0.22	-	1.83×10^{-9}	9.41±0.40	8.03±0.40	Sigma Aldrich (Cerilliant)

Compound	CAS no.	Molecular Formula	Molecular Weight	Water Solubility (mg/L)[i]	Log Kow[ii]	Log Koc[iii]	Log Dow[iv]	Henry's Law Constant (atm m^3mol^{-1})[v]	Vapour Pressure (Torr)[vi]	pKa (Most acidic)[Vii]	pKa (Most basic)[vii]	Supplier
Thiamethoxam	153719-23-4	$C_8H_{10}ClN_5O_3S$	291.71	$2.86x10^3$	0.8	1.365	1.29	$6.87x10^{-15}$	$1.36x10^{-9}$	0.99±0.10	-	LGC (Ultra)
Imidacloprid	138261-41-3	$C_9H_{10}ClN_5O_2$	255.66	$7.17x10^3$	0.56	1.527	-3.75	$1.04x10^{-13}$	$5.07x10^{-8}$	7.16±0.20	4.80±0.20	Sigma Aldrich (PESTANAL)
Clothianidin	210880-92-5	$C_6H_8ClN_5O_2S$	249.68	$6.00x10^3$	0.64	1.444	0.41	$9.21x10^{-16}$	$5.16x10^{-7}$	2.76±0.50	-0.2±0.10	Sigma Aldrich (PESTANAL)
Metazachlor	67129-08-2	$C_{14}H_{16}ClN_3O$	277.75	250	2.38	2.455	2.98	$5.80x10^{-11}$	$6.50x10^{-8}$	-	1.54±0.10	LGC (Dr Ehrenstorfer)
Terbuthylazine	5915-41-3	$C_9H_{16}ClN_5$	229.71	20.4	3.27	2.49	2.48	$5.94x10^{-9}$	$9.19x10^{-6}$	-	2.69±0.10	Sigma Aldrich
Methiocarb	2032-65-7	$C_{11}H_{15}NO_2S$	225.31	104	2.87	2.444	3.13	$1.14x10^{-9}$	$5.92x10^{-4}$	12.16±0.46	-1.52±0.70	Sigma Aldrich (PESTANAL)
Dichlofluanid	1085-98-9	$C_9H_{11}Cl_2FN_2O_2S$	333.23	5.41	2.72	3.068	3.22	$6.74x10^{-7}$	$1.10x10^{-4}$	-5.37±0.50	-	Sigma Aldrich (PESTANAL)
Flufenacet	142459-58-3	$C_{14}H_{13}F_4N_3O_2S$	363.33	9.52	2.39	3.102	3.22	$2.00x10^{-11}$	$1.18x10^{-6}$	-	0.31±0.50	LGC (Dr Ehrenstorfer)
Oxadiazon	19666-30-9	$C_{15}H_{18}Cl_2N_2O_3$	345.22	0.53	4.81	3.532	5.31	$3.22x10^{-8}$	$3.65x10^{-7}$	-2.73±0.40	-	Sigma Aldrich (PESTANAL)
Chlorpyrifos	2921-88-2	$C_9H_{11}Cl_3NO_3PS$	350.59	11.0	5.11	3.898	4.78	$2.52x10^{-6}$	$1.63x10^{-5}$	-	-5.28±0.10	LGC (Dr Ehrenstorfer)
Triallate	2303-17-5	$C_{10}H_{16}$-Cl_3NOS	304.66	1.36	4.57	3.343	3.8	$5.66x10^{-6}$	$2.86x10^{-4}$	-1.48±0.70	-	Sigma Aldrich (PESTANAL)
Tylosin	1401-69-0	$C_{46}H_{77}NO_{17}$	916.12	0.50	1.05	0.205	2.14	$5.77x10^{-38}$	0.00	13.06±0.70	7.39±0.70	LGC
Sulfapyridine	144-83-2	$C_{11}H_{11}N_3O_2S$	249.29	$1.20x10^4$	0.53	1.413	0.21	$1.08x10^{-13}$	$3.90x10^{-9}$	8.54±0.30	2.13±0.10	Sigma Aldrich
Sarafloxacin	98105-99-8	$C_{20}H_{17}F_2N_3O_3$	385.36	$1.14x10^3$	1.07	0.791	0.45	$1.92x10^{-19}$	$2.67x10^{-16}$	6.17±0.41	8.68±0.10	Sigma Aldrich (VETRANAL)
Ceftiofur	80370-57-6	C19H17N5O7S3	523.56	22.8	1.57	0.7	-2.49	$2.18x10^{-25}$	-	2.62±0.50	1.70±0.10	Sigma Aldrich
Diazinon	333-41-5	$C_{12}H_{21}N_2O_3PS$	304.35	6.46	3.86	3.339	4.19	$8.73x10^{-8}$	$7.07x10^{-5}$	1.21±0.30	-	Sigma Aldrich (PESTANAL)

[i] As calculated by EPI Suite [1] at 25°C

[ii] As calculated by EPI Suite [1] (KOWWIN v1.68 estimate)

[iii] As calculated by EPI Suite [1] based on Log Know

[iv] As calculated by Marvin Beans [2] at pH 7.5

[v] As calculated by EPI Suite [1] based on Bond SAR method

[vi] As stated on Scifinder calculated using Advanced Chemistry Development (ACD/Labs) Software v11.02 (© 1994-2015 ACD/Labs)[3]

[vii] As stated on Scifinder calculated using Advanced Chemistry Development (ACD/Labs) Software v11.02 (© 1994-2015 ACD/Labs) at 25°C [3]

Table S2 MS/MS detection parameters for analytes of interest sorted by class, includes analytes from ESI –ve and ESI +ve methods

Class of Analyte	Analyte	Method	Precursor (m/z)	Product Ion 1 (Q)	CV (V)	CE (eV)	Product Ion 2 (C)	CV (V)	CE (eV)	Internal Standard
UV filters	Benzophenone-1	C-ESI-	213.0	134.8	36	20	90.8	34	25	Ibuprofen-d3
	Benzophenone-2	C-ESI-	244.9	134.9	32	13	108.9	32	20	Bisphenol A-d16
	Benzophenone-3	B-ESI+	229.0	151.0	35	18	105.0	35	20	Methadone-d9
	Benzophenone-4	C-ESI-	307.0	227.1	44	24	211.1	42	35	Methylparaben-13C
Parabens	Methylparaben	C-ESI-	150.8	91.8	34	20	135.8	20	14	Methylparaben-13C
	Ethylparaben	C-ESI-	164.9	91.9	20	20	136.6	26	14	Naproxen-d3
	Propylparaben	C-ESI-	179.0	91.8	34	25	136.0	20	16	Bisphenol A-d16
	Butylparaben	C-ESI-	193.1	91.8	34	25	136.0	40	16	E1 (2,4,16,16)-d4
Plasticizer	Bisphenol-A	C-ESI-	227.3	212.1	40	22	132.7	40	25	Bisphenol A-d16
Steroid estrogens	E1	C-ESI-	269.0	145.0	55	40	158.0	55	40	E1 (2,4,16,16)-d4
	E2	C-ESI-	271.1	183.0	60	40	144.9	60	45	E2 (2,4,16,16)-d4
	EE2	C-ESI-	295.2	144.9	60	45	158.8	60	40	E2 (2,4,16,16)-d4
Antibacterials/antibiotics	Sulfasalazine	C-ESI-	369.8	197.0	45	25	239.9	45	25	Naproxen-d3
	Clarithromycin	B-ESI+	748.5	158.1	40	31	590.4	40	20	Methadone-d9
	Azithromycin	A-ESI+	749.5	116.1	60	54	83.1	60	60	Verapamil-d7
	Trimethoprim	B-ESI+	291.2	320.2	26	26	123.1	26	36	Methamphetamine-d5
	Sulfamethoxazole	B-ESI+	254.1	92.2	36	30	156.1	36	20	Benzoylecgonine-d8
	Triclosan	C-ESI-	288.8	34.8	18	10	36.8	18	10	E1 (2,4,16,16)-d4
	Amoxicillin	A-ESI+	365.8	113.9	22	23	207.8	22	13	Methiocarb-d3
	Ciprofloxacin	A-ESI+	331.5	288.0	43	18	313.6	43	21	Ciprofloxacin-d8
	Erythromycin	A-ESI+	734.3	576.0	34	21	558.3	34	21	Erythromycin-13C-d3
	Ofloxacin	A-ESI+	361.9	312.9	35	19	261.0	35	30	Ofloxacin-d3
	Metronidazole	A-ESI+	172.16	82.09	28	26	111.0	28	24	Metronidazole-d4
	Sulfadiazine	A-ESI+	250.9	107.9	30	26	157.8	30	15	Metronidazole-d4
	Norfloxacin	A-ESI+	320.22	233.2	58	26	204.9	58	38	Ofloxacin-d3
	Cefalexin	A-ESI+	348.22	158.04	48	6	174.0	48	4	Cefalexin-d5
	Penicillin V	A-ESI+	351.16	114.15	54	40	160.1	54	40	Metazachlor-d6
	Penicillin G	A-ESI+	335.16	160.06	48	20	217.1	48	16	Cefalexin-d5
	Chloramphenicol	A-ESI+	323.0	304.8	20	10	274.8	20	10	Methiocarb-d3
	Danofloxacin	A-ESI+	358.1	340.0	65	20	255.2	38	38	Ciprofloxacin-d8
	Prulifloxacin	A-ESI+	462.2	444.2	40	30	360.1	40	44	Ofloxacin-d3
Funcigide	Griseofulvin	A-ESI+	353.2	69.0	45	25	165.0	45	23	Methiocarb-d3

Class of Analyte	Analyte	Method	Precursor (m/z)	Product Ion 1 (Q)	CV (V)	CE (eV)	Product Ion 2 (C)	CV (V)	CE (eV)	Internal Standard
	Ketoconazole	A-ESI+	532.0	82.0	60	50	112.13	60	58	Amitriptyline-d3
Hypertension	Valsartan	C-ESI-	434.0	350.1	35	20	179.1	35	25	Naproxen-d3
	Irbesartan	C-ESI-	427.1	193.0	50	28	121.0	50	65	Bisphenol A-d16
	Lisinopril	B-ESI+	406.2	84.0	38	27	246.1	38	22	Amphetamine-d5
NSAIDs	Ketoprofen	C-ESI-	253.0	209.0	15	7	-	-	-	Ketoprofen-d3
	Ibuprofen	C-ESI-	204.9	161.5	26	6	-	-	-	Ibuprofen-d3
	Naproxen	C-ESI-	229.0	185.0	20	7	170.0	20	13	Naproxen-d3
	Diclofenac	C-ESI-	293.8	249.9	22	13	-	-	-	Naproxen-d3
	Acetaminophen	B-ESI+	151.9	110.0	26	16	211.2	22	10	Acetaminophen-d4
Lipid regulators	Bezafibrate	C-ESI-	360.1	274.0	30	19	154.0	30	28	Bezafibrate-d6
	Atorvastatin	C-ESI-	557.1	397.1	50	30	278.1	50	45	Naproxen-d3
Anti-hyperlipidemic	Gemfibrozil	A-ESI+	251	205.0	21	9	123.0	21	14	Methiocarb-d3
Anti-hyperintensive	Candesartan Cilexetil	A-ESI+	611.1	567.0	44	7	466.9	44	7	Cefalexin-d5
Antihistamines	Fexofenadine	C-ESI-	500.1	456.1	33	14	378.1	33	19	Naproxen-d3
	Cetirizine	B-ESI+	389.1	201.0	32	21	166.0	32	40	Temazepam-d5
GUD/ED	Sildenafil	A-ESI+	475.3	99.9	60	31	282.9	60	43	Sildenafil-d8
Diabetes	Metformin	B-ESI+	130.0	60.0	30	15	71.0	30	20	Metformin-d6
	Gliclazide	B-ESI+	324.1	127.0	41	20	110.0	41	20	Quetiapine-d8
	Sitagliptin	A-ESI+	407.8	234.9	46	19	192.9	46	26	Verapamil-d7
Cough suppressant	Pholcodine	B-ESI+	399.2	381.2	55	25	100.1	55	37	Atenolol-d7
Beta-blocker	Atenolol	B-ESI+	267.3	145.1	38	30	190.1	38	16	Atenolol-d7
	Metoprolol	B-ESI+	268.1	116.1	42	20	82.1	40	31	Metoprolol-d7
	Propranolol	B-ESI+	260.2	183.1	42	18	116.1	42	16	Propranolol-d7
	Bisoprolol	A-ESI+	326.0	116.0	45	18	204.0	45	19	Sildenafil-d8
H_2 receptor agonists	Ranitidine	B-ESI+	315.9	176.0	26	17	123.9	26	24	Atenolol-D7
	Cimetidine	B-ESI+	252.9	159.4	22	16	211.2	22	10	Acetaminophen-d4
X-ray contrast media	Iopromide	B-ESI+	792.0	573.0	46	25	558.9	46	32	Acetaminophen-d4
Various uses	Buprenorphine	A-ESI+	468.4	396.0	66	41	414.0	66	35	Amitriptyline-d3
Drug precursor and metabolite	Ephedrine/pseudoephedrine	B-ESI+	166.1	148.1	23	12	133.1	23	21	1S,2R-(+)-Ephedrine-d3
	Norephedrine	B-ESI+	152.2	134.1	23	10	117.1	23	16	1S,2R-(+)-Ephedrine-d3
Anti-cancer	Azathioprine	B-ESI+	278.0	142.0	28	13	85.0	28	20	Cotinine-d3
	Methotrexate	B-ESI+	455.1	308.1	40	20	175.1	40	35	Amphetamine-d5
	Ifosfamide	B-ESI+	261.0	92.0	40	28	154.0	40	22	Metoprolol-d7
	Tamoxifen	B-ESI+	372.2	72.0	50	25	129.0	50	28	Tamoxifen-13C2-15N

Class of Analyte	Analyte	Method	Precursor (m/z)	Product Ion 1 (Q)	CV (V)	CE (eV)	Product Ion 2 (C)	CV (V)	CE (eV)	Internal Standard
	Capecitabine	A-ESI+	359.9	243.9	25	11	173.8	25	23	Metazachlor-d6
	Imatinib	A-ESI+	494.2	394	57	27	378.3	57	48	Nortriptyline-d3
	Bicalutamide	A-ESI+	428.8	254.8	23	15	410.7	23	15	Methiocarb-d3
Anaesthetic and metabolite	Ketamine	B-ESI+	238.1	125.0	31	27	220.1	31	15	Ketamine-d4
	Norketamine	B-ESI+	224.0	207.1	23	12	125.0	23	27	Norketamine-d4
Anti-depressants and metabolites	Venlafaxine	B-ESI+	278.2	58.1	27	40	260.1	27	12	Metoprolol-d7
	Desvenlafaxine	B-ESI+	264.0	107.1	25	24	246.3	25	20	Metoprolol-d7
	Fluoxetine	B-ESI+	310.2	44.1	34	10	148.1	34	10	Fluoxetine-d5
	Norfluoxetine	B-ESI+	296.1	134.1	18	6	-	-	-	Fluoxetine-d5
	Sertraline	B-ESI+	306.0	159.0	23	27	275.0	23	10	Sertraline-d3
	Mirtazapine	B-ESI+	266.1	195.0	44	26	72.0	44	18	Mirtazapine-d3
	Citalopram	B-ESI+	325.1	262.1	46	18	109.9	46	26	Citalopram-d6
	Desmethylcitalopram	B-ESI+	311.4	109.0	46	27	262.0	46	18	Citalopram-d6
	Amitriptyline	A-ESI+	278.2	91.1	37	26	233.2	37	18	Amitriptyline-d3
	Nortriptyline	A-ESI+	264.2	233.1	33	16	91.0	33	23	Nortriptyline-d3
	Duloxetine	A-ESI+	297.9	153.9	16	5	187.9	16	5	Nortriptyline-d3
	Paroxetine	A-ESI+	330.0	192.0	45	21	150.9	45	22	Nortriptyline-d3
	Norsertraline	A-ESI+	291.8	158.6	25	23	128.8	25	22	Norsertraline-d4
Anti-epileptic and metabolites	Carbamazepine	B-ESI+	327.0	194.1	40	20	179.1	40	38	Carbamazepine-13C6
	Carbamazepine10,11-epoxide	B-ESI+	253.1	180.1	39	25	210.1	39	12	Carbamazepine-13C6
	10,11-Dihydro-10-hydroxycarbamazepine	B-ESI+	255.1	194.1	20	20	179.1	20	40	Carbamazepine-13C6
Calcium channel blocker	Diltiazem	B-ESI+	415.0	178.0	40	25	310.1	40	25	Carbamazepine-13C6
	Verapamil	A-ESI+	455.2	165.0	55	31	303.1	55	26	Verapamil-d7
Hypnotic	Temazepam	B-ESI+	301.1	255.1	37	21	283.1	37	14	Temazepam-d5
	Diazepam	A-ESI+	285.0	153.9	56	29	221.8	56	27	Diazepam-d5
	Oxazepam	A-ESI+	286.9	240.8	38	22	268.8	38	14	Oxazepam-d5
Anti-psychotic	Quetiapine	B-ESI+	384.1	253.1	50	21	221.1	50	40	Quetiapine-d8
	Risperidone	A-ESI+	411.0	190.9	49	30	109.9	49	51	Verapamil-d7
Dementia	Donepezil	A-ESI+	380.3	362.1	56	23	288.0	56	24	Verapamil-d7
	Memantine	A-ESI+	180.0	107.0	36	24	121.0	36	24	Verapamil-d7
Human indicators and metabolites	Creatinine	B-ESI+	114.0	44.0	30	15	86.1	31	11	Metformin-d6
	Nicotine	B-ESI+	163.1	130.0	37	20	117.0	37	20	Atenolol-d7
	Caffeine	B-ESI+	195.1	138.0	38	15	110.0	38	23	Cotinine-d3

Class of Analyte	Analyte	Method	Precursor (m/z)	Product Ion 1 (Q)	CV (V)	CE (eV)	Product Ion 2 (C)	CV (V)	CE (eV)	Internal Standard
	Cotinine	B-ESI+	177.1	80.0	34	21	98.1	34	22	Cotinine-d3
	1,7 dimethylxantine	B-ESI+	181.0	124.1	54	21	-	-	-	Cotinine-d3
Analgaesics and metabolites	Morphine	B-ESI+	286.2	165.1	53	38	152.1	53	56	Morphine-d3
	Dihydromorphine	B-ESI+	288.2	185.0	28	42	213.0	28	32	Morphine-d3
	Normorphine	B-ESI+	272.1	165.0	45	43	152.1	45	49	Morphine-d3
	Methadone	B-ESI+	310.2	265.1	31	15	105.1	31	28	Methadone-d9
	EDDP	B-ESI+	278.2	234.1	50	29	249.1	50	24	EDDP-d3
	Codeine	B-ESI+	300.2	215.1	49	25	152.1	49	57	Codeine-d6
	Norcodeine	B-ESI+	286.1	165.1	46	40	268.2	46	20	Codeine-d6
	Dihydrocodeine	B-ESI+	302.1	199.1	53	33	128.1	53	60	Codeine-d6
	Tramadol	B-ESI+	264.0	58.0	28	45	120.7	28	46	Metoprolol-d7
	N-desmethyltramadol	B-ESI+	250.1	44.0	25	12	232.1	25	8	Cocaine-d3
	O-desmethyltramadol	B-ESI+	250.2	58.0	30	18	232.1	30	10	Cotinine-d3
Stimulants and metabolites	Amphetamine	B-ESI+	136.2	91.1	18	16	119.1	18	8	Amphetamine-d5
	Methamphetamine	B-ESI+	150.2	91.1	18	16	119.1	24	10	Methamphetamine-d5
	MDMA	B-ESI+	194.1	163.1	24	13	105.1	24	24	MDMA-d5
	MDA	B-ESI+	180.0	163.1	21	11	105.1	21	22	MDA-d5
	Cocaine	B-ESI+	304.2	182.1	40	20	82.1	40	31	Cocaine-d3
	Benzoylecgonine	B-ESI+	290.2	168.1	38	19	105.1	38	30	Benzoylecgonine-d8
	Anhydroecgoninemethylester	B-ESI+	182.1	118.0	39	23	122.1	37	20	Atenolol-d7
	Cocaethylene	B-ESI+	318.2	196.2	38	20	82.1	38	30	Cocaethylene-d3
	Mephedrone	B-ESI+	178.1	160.1	10	12	145.0	10	22	Mephedrone-d3
	MDPV	B-ESI+	276.1	126.1	40	28	135.0	40	25	Cocaethylene-d3
Opioid and metabolite	Heroin	B-ESI+	370.2	165.1	51	50	268.1	51	29	Heroin-d9
	6-acetylmorphine	B-ESI+	328.1	165.1	52	39	211.1	52	26	Cotinine-d3
Pesticides, Fungicides and Herbicides	Dichlofluanid	A-ESI+	334.7	122.9	29	31	270.8	29	6	Amitriptyline-d3
	Oxadiazon	A-ESI+	348.1	331.0	18	7	313.1	18	12	Methiocarb-d3
	Triallate	A-ESI+	305.8	127.8	34	13	144.7	34	26	Methiocarb-d3
	Methiocarb	A-ESI+	225.9	208.8	34	15	174.9	34	19	Methiocarb-d3
	Clothianidin	A-ESI+	249.8	131.8	28	15	112.9	28	25	Methiocarb-d3
	Imidacloprid	A-ESI+	255.9	208.8	34	15	174.9	34	19	Metazachlor-d6
	Thiamethoxam	A-ESI+	291.8	210.9	44	12	131.9	44	22	Methiocarb-d3
	Terbuthylazine	A-ESI+	229.9	173.9	35	17	131.9	35	24	Diazepam-d5
	Metazachlor	A-ESI+	277.9	209.8	21	21	133.9	21	10	Metazachlor-d6

Class of Analyte	Analyte	Method	Precursor (m/z)	Product Ion 1 (Q)	CV (V)	CE (eV)	Product Ion 2 (C)	CV (V)	CE (eV)	Internal Standard
	Chlorpyrifos	A-ESI+	349.8	124.8	34	19	197.8	34	16	Metazachlor-d6
	Flufenacet	A-ESI+	363.8	152.0	24	18	194.0	24	10	Metazachlor-d6
Veterinary Pharmaceuticals	Diazinon	A-ESI+	304.9	168.9	36	22	152.9	36	22	Oxazepam-d5
	Tylosin	B-ESI+	916.5	174.2	80	45	101.0	80	56	Methadone-d9
	Oxytetracycline	A-ESI+	460.9	443.5	32	20	425.8	32	13	Ofloxacin-d3
	Sarafloxacin	A-ESI+	385.9	367.8	49	23	298.8	49	28	Verapamil-d7
	Sulfapyridine	A-ESI+	250.03	156.04	40	16	92.0	42	30	Metronidazole-d4
	Tetracycline	A-ESI+	444.9	409.9	33	20	427.5	33	14	Ciprofloxacin-d8
	Ceftiofur	A-ESI+	524.0	241.0	50	50	241.0	50	20	Ofloxacin-d3

Table S3 MS/MS detection parameters for internal standards sorted by analogue analyte class, includes internal standards from ESI –ve and ESI +ve methods

ISTD	Method	Precursor (m/z)	Product Ion 1 (Q)	CV (V)	CE (eV)	ISTD	Method	Precursor (m/z)	Product Ion 1 (Q)	CV (V)	CE (eV)
Metronidazole-d4	A-ESI+	175.9	127.9	26	15	Norketamine-d4	B-ESI+	228.1	128.9	32	28
Gabapentin-d4	A-ESI+	176	158.1	33	16	Metoprolol-d7	B-ESI+	275.4	123.1	44	20
Methiocarb-d3	A-ESI+	228.9	168.9	25	9	Cocaine-d3	B-ESI+	307.2	185.1	40	20
Nortriptyline-d3	A-ESI+	267.1	191	40	20	Cocaethylene-d3	B-ESI+	321.2	199.1	40	22
Amitriptyline-d3	A-ESI+	281.5	90.9	40	25	Mirtazapine-d3	B-ESI+	269.0	194.9	35	25
Metazachlor-d6	A-ESI+	284	215.9	21	10	EDDP-d3	B-ESI+	281.2	234.1	50	29
Diazepam-d5	A-ESI+	290.1	153.9	56	28	Propranolol-d7	B-ESI+	267.0	188.8	40	18
Oxazepam-d5	A-ESI+	292.0	245.9	38	22	Citalopram-d6	B-ESI+	331.0	109.0	46	28
Norsertraline-d4	A-ESI+	296	278.8	39	8	Carbamazepine-13C6	B-ESI+	243.1	200.1	40	20
Ciprofloxacin-d8	A-ESI+	340.1	321.9	55	21	Quetiapine-d8	B-ESI+	392.1	258.1	50	23
Cefalexin-d5	A-ESI+	353.2	194.5	43	10	Methadone-d9	B-ESI+	319.3	268.2	31	15
Ofloxacin-d3	A-ESI+	364.8	260.8	47	28	Temazepam-d5	B-ESI+	306.7	260.1	37	21
Verapamil-d7	A-ESI+	462.1	165	56	31	Fluoxetine-d5	B-ESI+	315.3	153.2	26	8
Sildenafil-d8	A-ESI+	482.9	108.0	60	31	Sertraline-d3	B-ESI+	309.1	159.0	23	27
Erythromycin-13C,D3	A-ESI+	738.5	162	46	33	Tamoxifen-13C2-15N	B-ESI+	375.1	75.0	50	25
Metformin-d6	B-ESI+	136.1	77.0	30	19	Methylparaben-13C	C-ESI-	156.9	97.9	30	20
Morphine-d3	B-ESI+	289.1	152.1	53	56	Ketoprofen-d3	C-ESI-	265.0	212.0	15	7
Atenolol-d5	B-ESI+	274.3	145.1	44	30	Bezafibrate-d6	C-ESI-	366.0	274.0	30	19
Acetaminophen-d4	B-ESI+	156.0	114.0	26	16	Naproxen-d13	C-ESI-	232.0	188.0	15	8
Codeine-d6	B-ESI+	306.2	218.1	52	28	Bisphenol A-d16	C-ESI-	241.1	223.1	40	20
Cotinine-d3	B-ESI+	180.1	80.0	44	24	Ibuprofen-d3	C-ESI-	208.0	164.0	20	6
1S,2R-(+) Ephedrine-d3	B-ESI+	169.2	151.0	23	18	E2 (2, 4, 16, 16)-d4	C-ESI-	275.1	147.0	60	40
Amphetamine-d5	B-ESI+	141.1	92.8	20	14	E1 (2, 4, 16, 16)-d4	C-ESI-	273.1	147.0	55	40
Methamphetamine-d5	B-ESI+	155.1	91.8	28	18						
MDA-d5	B-ESI+	185.1	168.1	21	11						
MDMA-d5	B-ESI+	199.1	165.1	26	13						
Benzoylecgonine-d8	B-ESI+	298.2	171.1	38	19						
Mephedrone-d3	B-ESI+	181.1	148.0	30	22						
Ketamine-d4	B-ESI+	242.1	129.1	31	27						
Heroin-d9	B-ESI+	379.2	165.8	51	50						

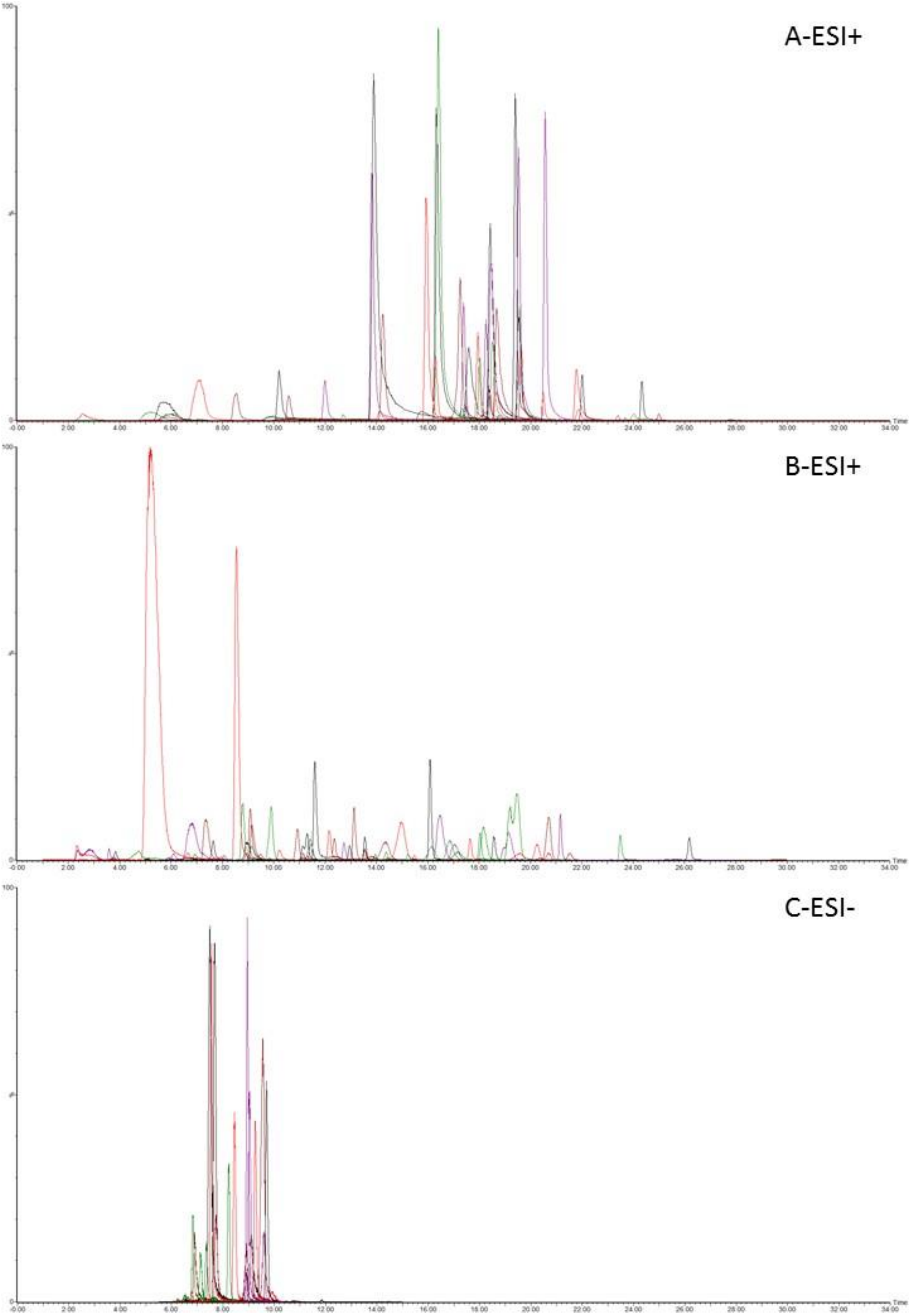

Fig. S1 Chromatograms of all analytes in spiked influent sample from Site A using methods: A-ESI+, B-ESI+ and C-ESI-. The run time for C-ESI- is 22.5 minutes, which is shorter than the 34 minute run time of A-ESI+ and B-ESI+, hence the much more condensed appearance of the peaks.

Table S4 Solid sample weights

Digested solids

Sample No.	1	2	3	4	5	6
Day	1	1	2	2	3	3
Weight (g)	0.24725	0.24989	0.24604	0.24189	0.24855	0.24137

Solid Particulate matter

Sample No.	1	2	3	4	5	6	7	8	9	10	11	12	13	14
Day	1	1	2	2	3	3	4	4	5	5	6	6	7	7
Weight (g)	0.23660	0.22825	0.23961	0.23985	0.23428	0.23437	0.23800	0.24990	0.24075	0.23251	0.23590	0.23546	0.24345	0.23930

Table S5 Suspended solids weights (g L^{-1})

**Wed**	**Thu**	**Fri**	**Sat**	**Sun**	**Mon**	**Tue**
**Day 1**	**Day 2**	**Day 3**	**Day 4**	**Day 5**	**Day 6**	**Day 7**
0.2094	0.2072	0.2376	0.2006	0.1634	0.1134	0.147

Table S6 Adsorbance

Compound	Spiked Surface water adsorbance to PPE bottle after 24h storage		Recovery 24h storage on SPE cartridge	
	Ads (%)	SD	Rec (%)	SD
Benzophenone-1	-	-	-	-
Benzophenone-2	-	-	-	-
Benzophenone-3	33.7	18.7	45.3	16.0
Benzophenone-4	-	-	-	-
Methylparaben	-	-	-	-
Ethylparaben	-	-	-	-
Propylparaben	-	-	-	-
Butylparaben	-	-	-	-
Bisphenol A	-	-	-	-
E1	-	-	-	-
E2	-	-	-	-
EE2	-	-	-	-
Sulfasalazine	-	-	-	-
Clarithromycin	-6.4	2.1	106.8	11.8
Trimethoprim	5.2	3.0	94.7	4.3
Sulfamethoxazole	9.3	0.1	98.8	3.9
Triclosan	-	-	-	-
Amoxicillin	-	-	-	-
Metronidazole	-6.8	4.8	107.4	2.1
Sulfadiazine	37.6	3.6	97.9	10.9
Cefalexin	-	-	-	-
Ofloxacin	18.1	16.4	96.8	6.4
Ciprofloxacin	-	-	-	-
Tetracycline	-	-	-	-
Danofloxacin	-	-	-	-
Oxytetracycline	-	-	-	-
Chloramphenicol	-9.7	16.1	126.5	10.7
Penicillin G	-	-	-	-
Penicillin V	-60.9	55.6	149.7	41.9
Erythromycin	-4.0	11.2	146.2	71.1
Azithromycin	16.6	2.3	115.0	21.6
Prulifloxacin	-	-	-	-
Norfloxacin	-	-	-	-
Griseofulvin	-0.3	8.4	98.2	3.2
Ketoconazole	-10.6	2.9	104.6	13.6
Valsartan	-	-	-	-
Irbesartan	-	-	-	-
Lisinopril	73.7	20.8	22.0	1.6
Ketoprofen	-	-	-	-
Ibuprofen	-	-	-	-
Naproxen	-	-	-	-
Diclofenac	-	-	-	-

Compound	Spiked Surface water adsorbance to PPE bottle after 24h storage		Recovery 24h storage on SPE cartridge	
	Ads (%)	SD	Rec (%)	SD
Acetaminophen	1.4	3.1	97.3	2.7
Bezafibrate	-	-	-	-
Atorvastatin	-	-	-	-
Gemfibrozil	-15.5	11.2	107.0	4.2
Candesartan Cilexetil	-141.2	53.2	322.5	208.8
Fexofenadine	-	-	-	-
Cetirizine	1.6	0.7	89.8	6.1
Sildenafil	-9.8	7.6	107.1	3.9
Metformin	-62.3	5.1	71.5	10.4
Gliclazide	6.3	6.6	107.3	3.3
Sitagliptin	15.6	5.0	149.4	34.5
Pholcodine	43.4	1.8	65.2	4.4
Atenolol	-6.8	3.8	109.6	1.5
Metoprolol	7.8	1.0	98.8	1.3
Propranolol	-12.4	7.3	108.2	16.1
Bisoprolol	6.0	10.1	118.5	22.0
Ranitidine	30.7	5.7	118.8	5.8
Cimetidine	52.5	0.7	99.2	2.4
Iopromide	-11.2	2.5	108.1	3.1
Buprenorphine	0.5	1.5	151.6	26.9
Ephedrine/pseudoephedrine	52.9	0.8	93.5	5.1
Norephedrine	18.0	1.8	129.5	23.5
Azathioprine	5.6	3.3	95.6	1.3
Methotrexate	30.2	0.8	57.0	7.2
Ifosfamide	5.5	0.1	99.3	0.3
Tamoxifen	60.2	5.9	73.0	26.7
Imatinib	11.7	2.2	101.9	17.7
Capecitabine	-1.1	2.2	94.2	4.7
Bicalutamide	-19.7	14.4	95.3	14.0
Ketamine	-2.7	0.7	110.2	2.5
Norketamine	5.4	1.3	98.4	1.0
Venlafaxine	3.8	1.3	101.9	2.6
Desvenlafaxine	-1.3	1.8	103.1	3.7
Fluoxetine	-22.2	21.6	121.4	32.9
Norfluoxetine	58.4	9.2	71.3	36.6
Sertraline	1.4	20.6	96.0	25.8
Mirtazapine	6.0	1.6	100.4	6.6
Citalopram	2.5	4.6	92.4	14.5
Desmethylcitalopram	-2.0	7.0	110.1	17.9
Paroxetine	35.8	15.7	85.4	16.4
Duloxetine	-	-	-	-
Amitriptyline	14.0	3.1	95.8	6.2
Nortriptyline	30.0	0.8	110.0	9.8
Norsertraline	-	-	-	-

Compound	Spiked Surface water adsorbance to PPE bottle after 24h storage		Recovery 24h storage on SPE cartridge	
	Ads (%)	SD	Rec (%)	SD
Carbamazepine	6.3	2.1	96.2	0.7
Carbamazepine 10,11 epoxide	-7.8	0.9	109.5	0.8
10,11 dihydro 10 hydroxycarbamazepine	17.4	0.7	82.4	0.1
Diltiazem	-2.0	3.9	91.6	17.3
Verapamil	9.1	3.0	94.5	4.9
Temazepam	-0.5	2.2	100.8	0.7
Oxazepam	14.9	0.4	89.6	0.5
Diazepam	-17.7	0.2	119.4	0.6
Quetiapine	-7.3	5.3	97.0	14.5
Risperidone	14.8	8.3	118.5	19.6
Donepezil	16.4	13.7	118.9	29.4
Memantine	3.2	10.4	174.0	41.0
Creatinine	75.4	0.7	37.8	7.3
Nicotine	-11.8	6.0	147.9	5.6
Caffeine	4.6	0.5	96.8	1.6
Cotinine	0.1	3.0	97.3	1.0
1,7 dimethylxantine	-4.4	3.7	99.1	6.2
Morphine	3.3	10.1	104.7	2.7
Dihydromorphine	24.1	2.6	90.2	0.5
Normorphine	17.0	4.5	107.4	3.3
Methadone	1.5	0.7	101.2	11.4
EDDP	53.7	1.3	98.3	11.8
Codeine	1.2	5.1	107.0	4.9
Norcodeine	6.6	7.0	106.7	5.3
Dihydrocodeine	14.7	0.2	95.1	0.7
Tramadol	1.9	2.3	101.8	0.1
N-desmethyltramadol	16.7	4.3	99.0	3.5
O-desmethyltramadol	4.0	0.4	99.2	2.0
Amphetamine	43.3	1.0	87.1	0.1
Methamphetamine	3.2	0.7	118.2	1.8
MDMA	21.0	2.5	90.2	2.8
MDA	44.4	2.5	82.1	4.1
Cocaine	1.4	0.1	99.7	2.0
Benzoylecgonine	1.0	0.7	95.9	0.2
Anhydroecgonine methylester	10.3	3.7	108.7	3.5
Cocaethylene	6.0	2.0	94.8	2.4
Mephedrone	33.6	4.3	90.1	7.3
MDPV	38.9	3.4	82.8	7.0
Heroin	17.8	0.8	87.7	4.4
O-6-MAM	15.6	2.8	93.8	2.9
Thiamethoxam	-1.7	6.0	106.1	2.2
Imidacloprid	1.1	1.0	95.2	2.3
Clothianidin	-1.8	9.7	107.5	5.9

Compound	Spiked Surface water adsorbance to PPE bottle after 24h storage		Recovery 24h storage on SPE cartridge	
	Ads (%)	SD	Rec (%)	SD
Metazachlor	2.1	6.7	96.9	14.2
Terbuthylazine	-13.9	3.7	119.0	0.8
Methiocarb	4.1	5.4	99.3	5.3
Dichlofluanid	-	-	-	-
Flufenacet	-9.7	0.6	103.1	1.3
Oxadiazon	-27.6	28.9	74.1	15.8
Chlorpyrifos	-3.3	0.3	112.0	5.8
Triallate	-44.7	33.0	136.8	9.9
Tylosin	11.4	7.5	86.0	1.4
Sulfapyridine	-2.9	3.9	103.7	1.0
Sarafloxacin	-	-	-	-
Ceftiofur	73.2	2.7	29.9	13.8
Diazinon	-8.9	10.9	106.0	16.8

Table S7 Instrument parameters (LC MSMS): Intra- and Inter-day precision and accuracy

	Inter-day precision (%)			Intra-day precision (%)			Inter-day Accuracy (%)			Intra-day Accuracy (%)		
**Compound**	**Low**	**Med**	**High**	**Low**	**Med**	**High**	**Low**	**Med**	**High**	**Low**	**Med**	**High**
Benzophenone-1	2.4	6.4	1.0	4.9	1.1	0.9	106.3	116.2	97.6	103.6	119.0	97.9
Benzophenone-2	4.1	6.1	2.6	1.3	1.2	0.8	90.8	106.4	95.6	90.4	110.0	98.2
Benzophenone-3	2.5	3.8	-	4.3	4.6	-	83.4	86.3	-	84.4	89.2	-
Benzophenone-4	7.0	3.3	1.1	2.5	2.7	1.8	117.0	92.3	105.8	115.0	89.0	105.0
Methylparaben	5.2	11.5	1.4	0.7	0.4	2.1	94.4	101.0	96.9	90.7	94.0	95.3
Ethylparaben	2.2	4.1	0.1	4.6	1.2	2.2	120.2	120.9	98.0	117.3	121.6	98.0
Propylparaben	2.8	9.3	-	2.3	12.2	-	99.8	102.9	-	97.1	100.4	-
Butylparaben	3.7	6.2	1.1	5.1	9.2	0.8	97.2	103.9	99.7	93.6	99.2	98.4
Bisphenol A	0.9	2.6	0.3	2.9	2.6	1.8	107.0	108.7	98.0	107.0	106.1	97.7
E1	3.5	1.8	1.0	2.0	1.6	1.7	99.1	97.7	98.9	96.2	96.7	97.9
E2	2.3	5.2	0.4	3.5	4.5	1.1	95.7	93.7	99.5	93.7	96.9	99.2
EE2	7.0	1.3	1.5	2.1	3.9	1.8	84.3	94.3	101.1	86.1	95.2	102.5
Sulfasalazine	3.4	1.4	2.4	4.0	4.3	3.3	112.3	100.4	101.3	115.4	101.3	98.8
Clarithromycin	5.1	1.5	1.3	4.0	2.0	1.2	97.5	99.9	102.0	102.6	99.3	103.6
Azithromycin	-	1.6	1.4	-	3.9	5.2	-	108.1	95.9	-	113.6	104.2
Trimethoprim	1.4	3.5	4.1	1.6	1.8	3.2	104.5	96.8	89.3	104.6	94.2	99.6
Sulfamethoxazole	2.0	3.0	5.6	0.7	2.2	4.3	104.3	91.6	89.3	106.1	93.6	88.3
Triclosan	2.9	13.7	2.8	10.0	16.1	2.0	69.8	72.4	72.1	69.4	66.3	73.2
Amoxicillin	8.1	6.9	5.2	8.8	3.2	3.9	93.5	88.8	100.8	104.2	99.1	113.8
Metronidazole	2.3	0.5	0.7	1.3	2.9	3.3	114.4	99.5	95.0	110.9	104.6	99.5
Sulfadiazine	1.1	1.8	1.6	1.9	3.3	3.0	126.7	90.7	95.9	123.8	93.0	99.2
Cefalexin	-	12.3	-	-	9.5		-	102.9	-	-	111.3	
Ofloxacin	2.8	3.8	1.9	6.8	3.4	2.3	98.0	95.3	94.3	97.6	98.3	96.3
Ciprofloxacin	7.0	6.0	3.3	16.8	6.9	2.4	75.5	97.5	97.7	58.1	104.4	104.4
Tetracycline	13.1	5.0	7.4	12.9	2.9	4.6	151.2	91.3	96.9	134.6	99.6	111.0
Danofloxacin	-	11.1	0.9	-	7.7	6.8	-	95.9	102.5	-	95.0	116.9
Oxytetracycline	-	5.0	1.1	-	3.9	5.2	-	85.7	92.0	-	87.4	99.7
Chloramphenicol	4.4	1.7		6.5	1.1		102.4	99.2		104.8	102.2	
Penicillin G	6.2	2.7		16.1	4.5		125.3	98.2		128.8	102.2	
Penicillin V	-	15.0	-	-	4.4		-	96.8	-	-	88.5	
Erythromycin	-	-	2.9	-		2.3	-	-	95.2	-		94.4
Prulifloxacin	-	3.0	14.7	-	2.2	6.5	-	100.7	72.2	-	106.3	91.0
Norfloxacin	-	1.5	7.3	-	4.1	4.0	-	82.1	88.1	-	84.4	86.6
Griseofulvin	3.4	2.6	-	2.2	1.0		88.8	94.4	-	86.1	92.4	
Ketoconazole	3.5	1.5	2.5	2.4	4.0	4.9	87.2	92.6	95.3	89.7	94.4	100.4

Compound	Inter-day precision (%)			Intra-day precision (%)			Inter-day Accuracy (%)			Intra-day Accuracy (%)		
	Low	Med	High	Low	Med	High	Low	Med	High	Low	Med	High
Valsartan	2.7	3.2	4.8	1.6	0.8	3.4	157.0	98.7	100.0	157.4	95.6	94.5
Irbesartan	6.1	3.8	2.2	4.2	2.9	0.8	85.1	108.6	101.3	83.3	108.7	98.8
Lisinopril	0.9	3.4	-	13.0	1.4	-	103.6	90.7	-	103.0	87.3	-
Ketoprofen	3.9	3.5	0.5	3.6	0.5	2.4	101.4	96.2	100.5	99.6	99.1	101.1
Ibuprofen	3.7	1.3	1.8	3.8	0.9	2.6	83.1	98.4	101.0	81.8	97.8	101.7
Naproxen	3.0	3.6	0.9	1.7	1.0	1.8	91.9	101.8	101.4	90.2	102.5	100.3
Diclofenac	5.5	5.0	2.9	8.6	9.9	5.3	80.4	97.9	97.2	78.0	95.5	95.3
Acetaminophen	1.6	1.6	1.6	4.3	0.9	2.7	96.4	98.0	97.7	98.1	99.3	99.5
Bezafibrate	4.3	1.4	2.7	4.2	2.0	0.7	92.6	100.3	100.8	91.4	99.5	102.3
Atorvastatin	1.6	7.9	1.1	4.7	2.5	0.6	100.9	98.2	103.8	101.9	89.6	102.6
Gemfibrozil	8.0	5.9	-	11.7	3.9		144.1	98.1	-	140.5	96.6	
Candesartan Cilexetil	-	-	0.9	-		5.2	-	-	106.9	-		100.5
Fexofenadine	4.4	13.6	1.3	1.6	3.4	1.3	101.5	103.9	108.4	98.4	111.4	109.0
Cetirizine	2.0	0.9	0.8	0.8	0.4	2.6	101.8	99.2	100.4	103.8	99.2	99.4
Sildenafil	1.7	4.8	2.6	3.8	2.2	4.6	99.3	106.0	91.9	95.8	110.6	92.2
Metformin	2.6	1.1	0.8	1.7	1.2	0.9	86.2	101.6	101.2	88.7	101.5	100.7
Gliclazide	0.8	4.4	1.1	2.5	5.0	0.8	86.7	92.5	100.3	87.3	97.2	101.5
Sitagliptin	1.8	5.7	1.5	0.9	4.1	4.6	141.1	88.7	101.2	141.5	92.8	100.8
Pholcodine	0.5	6.1	7.3	0.4	6.4	3.0	78.7	105.7	114.1	80.0	100.4	117.3
Atenolol	3.9	1.5	0.9	4.5	2.0	0.5	83.3	99.9	102.8	86.9	100.6	102.8
Metoprolol	2.0	1.2	0.6	0.9	3.2	1.8	91.3	98.1	101.1	90.9	96.7	100.7
Propranolol	2.1	1.5	2.5	0.4	0.7	1.9	122.5	96.8	97.0	125.4	98.4	94.8
Bisoprolol	3.3	0.7	2.1	8.0	2.9	3.4	86.6	109.8	91.5	90.4	115.0	95.7
Ranitidine	3.9	0.5	3.1	3.5	10.9	14.6	100.2	95.0	105.0	95.7	95.4	101.3
Cimetidine	5.2	2.6	4.7	3.0	13.8	10.1	105.3	98.2	108.8	99.0	96.0	102.9
Iopromide	7.0	5.2	2.6	14.7	10.3	11.2	94.0	100.6	109.0	100.1	103.8	112.2
Buprenorphine	13.7	9.4		11.4	6.5		80.1	96.3		92.2	96.7	
Ephedrine/pseudoephedrine	4.5	3.6	4.1	2.5	4.1	3.5	92.3	95.7	94.1	99.5	95.1	97.4
Norephedrine	5.6	4.1	3.2	7.2	4.8	3.3	101.2	92.3	95.4	97.6	89.5	98.6
Azathioprine	14.8	5.8	2.3	14.6	11.0	16.2	93.9	106.3	92.2	100.1	99.6	92.6
Methotrexate	11.9	7.5	6.8	0.8	5.0	6.5	108.1	103.6	112.3	119.7	110.1	106.8
Ifosfamide	2.9	3.1	1.3	4.0	3.3	0.7	94.5	90.6	95.6	96.4	92.5	96.9
Tamoxifen	4.5	1.8	5.6	2.2	0.7	4.3	88.8	106.1	93.2	93.0	105.2	92.3
Imatinib	-	1.5		-	2.5		-	101.3		-	103.8	
Capecitabine	3.4	3.7	1.4	2.7	2.1	2.1	79.9	93.9	95.2	79.8	93.4	94.4
Bicalutamide	-	2.9	2.9	-	1.9	3.5	-	80.4	103.6	-	79.9	100.2
Ketamine	2.7	1.7	1.1	0.4	1.8	1.6	75.6	103.6	98.5	77.3	105.3	98.2

Compound	Inter-day precision (%)			Intra-day precision (%)			Inter-day Accuracy (%)			Intra-day Accuracy (%)		
	Low	Med	High	Low	Med	High	Low	Med	High	Low	Med	High
Norketamine	2.9	1.9	0.6	4.8	3.7	0.9	80.3	99.4	102.4	82.2	98.1	101.8
Venlafaxine	3.9	2.0	1.6	1.4	1.3	2.4	74.6	96.2	102.7	72.9	96.7	101.7
Desmethylvenlafaxine	4.0	2.3	2.0	2.4	2.4	1.4	101.4	101.3	101.3	105.9	102.2	98.9
Fluoxetine	1.5	2.1	1.4	2.2	1.3	1.9	91.1	101.1	98.1	92.3	103.0	99.6
Norfluoxetine	2.5	1.6	0.4	4.1	2.9	2.3	107.0	103.3	97.8	106.3	104.8	98.1
Sertraline	3.0	1.0	0.9	0.7	1.7	2.7	84.0	100.8	101.1	84.3	101.9	100.9
Mirtazapine	3.9	4.2	2.1	2.2	3.0	2.9	90.3	99.0	95.0	94.0	101.6	97.3
Citalopram	0.5	0.8	0.8	1.7	4.9	1.2	101.6	100.5	101.7	102.0	101.3	102.1
Desmethylcitalopram	0.1	2.9	2.4	1.7	4.4	3.0	122.1	99.1	97.8	112.2	99.4	98.7
Paroxetine	-	1.5	1.1	-	2.6	3.8	-	101.4	102.7	-	105.4	101.4
Duloxetine	-	13.1	14.0	-	2.9	3.1	-	80.7	75.9	-	97.1	85.4
Amitriptyline	3.6	1.5	2.3	4.3	4.1	4.9	90.8	100.4	99.2	92.2	105.7	100.8
Nortriptyline	4.9	2.8	1.6	5.0	2.6	4.3	78.2	101.4	99.2	80.7	106.7	98.9
Norsertraline	15.2	6.8	-	13.1	4.2		76.9	106.7	-	85.8	112.3	
Carbamazepine	2.6	1.7	1.6	2.4	1.1	1.3	84.1	94.8	96.1	86.3	94.5	97.2
Carbamazepine 10,11-epoxide	0.9	0.1	3.7	2.0	2.4	2.0	94.8	80.4	91.3	94.0	80.5	95.1
10,11-Dihydro -10-hydroxycarbamazepine	3.7	2.0	2.8	5.1	3.8	7.9	94.6	96.5	85.4	94.8	98.6	88.1
Diltiazem	0.4	1.5	4.9	3.2	2.2	1.4	93.8	93.4	91.0	94.0	94.6	92.1
Verapamil	-	2.3	2.6	-	3.5	2.3	-	104.9	98.9	-	108.5	97.8
Temazepam	1.7	1.0	0.4	1.1	1.4	2.4	92.3	98.0	100.6	93.8	98.7	101.1
Oxazepam	6.3	2.3	1.8	3.4	3.6	3.0	82.8	100.7	99.4	81.3	103.7	99.5
Diazepam	4.7	5.0	3.7	2.8	0.8	1.3	105.6	99.1	94.2	107.2	101.0	93.9
Quetiapine	1.8	1.1	1.4	1.0	1.0	1.7	83.4	101.3	101.3	85.1	102.5	101.5
Risperidone	-	1.2		-	3.2		-	96.8		-	101.6	
Donepezil	-	1.8	0.7	-	3.3	1.9	-	111.0	104.3	-	115.5	106.1
Memantine	-	0.6	1.2	-	2.8	4.1	-	105.9	102.7	-	110.4	102.2
Creatinine	1.1	0.9	2.3	2.5	1.9	3.9	99.3	110.8	97.2	93.3	109.7	97.5
Nicotine	1.1	2.1	0.5	2.6	4.1	0.4	102.2	90.0	102.6	103.2	89.6	102.3
Caffeine	2.0	1.8	1.3	1.9	5.4	0.9	113.2	90.1	95.4	115.2	89.5	96.5
Cotinine	0.8	2.2	1.6	1.5	1.2	1.8	94.4	99.6	101.3	95.1	100.9	100.3
1,7-dimethylxanthine	13.0	2.9	2.1	6.8	9.0	13.9	84.2	99.9	98.7	90.0	98.4	96.3
Morphine	6.4	0.2	2.1	3.9	0.1	3.4	94.6	106.3	96.5	87.7	106.3	98.5
Dihydromorphine	3.8	2.5	6.8	0.4	2.5	5.3	110.6	102.0	105.3	115.1	104.0	106.3
Normorphine	2.2	1.5	0.9	3.6	1.1	1.9	101.1	102.7	99.1	98.6	102.3	98.5
Methadone	0.7	2.3	-	1.7	1.2	-	100.4	97.1	-	101.1	99.3	-
EDDP	1.4	0.5	1.8	1.3	1.2	1.0	92.6	102.1	94.5	91.2	102.6	95.5

Compound	Inter-day precision (%)			Intra-day precision (%)			Inter-day Accuracy (%)			Intra-day Accuracy (%)		
	Low	Med	High	Low	Med	High	Low	Med	High	Low	Med	High
Codeine	3.8	1.0	1.1	3.5	4.3	4.1	87.6	98.2	94.6	91.4	99.0	94.8
Norcodeine	3.5	2.4	2.4	6.7	4.2	3.4	105.7	94.7	95.2	106.3	95.1	94.2
Dihydrocodeine	2.7	0.5	1.4	0.8	1.7	3.7	84.8	103.4	94.4	87.4	102.8	93.4
Tramadol	1.7	1.7	1.5	1.2	2.6	1.9	100.6	100.6	99.0	99.0	99.0	97.3
N-desmethyltramadol	3.4	3.0	1.1	3.2	1.2	2.2	91.1	92.8	93.6	94.7	93.9	94.7
O-desmethyltramadol	2.0	4.6	-	3.1	6.7	-	92.6	97.9	-	94.2	102.8	-
Amphetamine	0.8	2.1	1.5	1.3	1.3	2.2	100.3	102.9	99.2	100.8	101.6	99.8
Methamphetamine	1.6	0.2	1.3	1.2	0.3	2.4	99.0	101.7	102.3	100.4	101.5	101.3
MDMA	0.6	1.2	2.1	3.3	0.5	1.2	102.5	96.7	98.3	103.2	97.9	98.3
MDA	1.4	1.1	0.7	0.6	0.4	1.1	98.4	99.6	97.3	100.0	100.8	99.3
Cocaine	2.2	2.7	1.7	1.1	1.1	2.2	96.7	98.1	96.8	98.2	100.4	98.4
Benzoylecgonine	1.4	3.3	2.5	0.7	1.1	0.9	104.3	101.6	104.4	105.2	103.2	101.4
Anhydroecgoninemethylester	1.6	2.5	2.8	1.2	2.3	3.8	109.1	90.8	103.5	107.1	88.5	100.4
Cocaethylene	5.9	0.8	1.6	2.0	1.2	1.8	88.2	101.8	95.2	88.3	101.2	94.5
Mephedrone	1.8	0.9	2.8	4.9	3.0	0.9	77.3	89.6	94.4	75.9	89.9	91.4
MDPV	4.2	2.3	0.2	0.3	0.6	1.1	105.8	94.1	99.0	108.7	96.5	99.1
Heroin	2.7	2.2	0.8	2.5	1.3	1.7	98.3	97.0	99.4	100.4	98.8	98.6
6-acetylmorphine	1.1	9.6	7.5	3.5	3.4	8.3	104.7	75.0	106.3	105.0	83.0	112.3
Thiamethoxam	8.6	2.3	-	5.6	3.8		93.0	100.9	-	89.9	97.7	
Imidacloprid	-	5.7	5.2	-	3.1	2.5	-	110.2	96.8	-	107.9	93.0
Clothianidin	4.1	3.7	2.0	3.5	2.8	3.3	88.7	106.4	100.8	91.1	105.3	97.4
Metazachlor	-	3.2	2.0	-	1.5	3.5	-	108.2	101.1	-	112.1	99.9
Terbuthylazine	1.1	2.5	6.5	1.8	2.3	2.9	91.4	103.7	97.3	94.3	106.1	98.9
Methiocarb	1.4	1.2	2.8	1.5	2.2	2.2	100.6	102.7	98.5	101.1	105.4	98.8
Dichlofluanid	-	3.2	5.5	-	1.3	6.3	-	89.1	92.6	-	93.3	96.5
Flufenacet	1.4	3.1	4.4	1.3	2.3	2.3	125.3	96.6	96.8	121.7	97.5	93.5
Oxadiazon	5.0	0.6		3.4	4.6		90.2	104.1		91.2	99.8	
Chlorpyrifos	10.4	5.3	-	12.1	11.5		71.8	94.9	-	73.1	88.2	
Triallate	13.2	-	-	7.6			70.6	-	-	81.3		
Tylosin	2.9	2.2	1.6	5.8	2.8	3.3	104.9	92.2	101.3	108.2	92.7	99.8
Sulfapyridine	1.5	0.5	1.3	2.6	2.3	3.1	145.9	86.6	96.1	143.7	89.4	99.2
Sarafloxacin	3.9	1.8	1.3	5.6	4.8	5.2	138.5	85.0	97.8	134.4	92.9	109.0
Ceftiofur	-	3.4	0.6	-	5.3	2.0	-	81.0	91.7	-	83.4	95.5
Diazinon	3.6	4.2	4.5	3.1	2.3	2.7	101.9	89.1	97.0	102.3	93.2	101.2

Table S8 Liquid matrix absolute and corrected recoveries

	Absolute						Corrected					
	Surface water		Effluent		Influent		Surface water		Effluent		Influent	
**Compound**	**Rec (%)**	**SD**	**Rec (%)**	**SD**	**Rec (%)**	**SD**	**Rec (%)**	**SD**	**Rec (%)**	**SD**	**Rec (%)**	**SD**
Benzophenone-1	81.3	0.1	80.2	1.1	49.5	1.4	99.9	14.8	89.2	11.9	75.6	2.4
Benzophenone-2	31.0	14.4	29.0	1.7	26.7	1.1	44.6	19.3	40.3	14.0	45.4	3.0
Benzophenone-3	33.0	52.5	51.9	10.0	27.0	32.2	74.4	33.7	89.4	29.9	58.5	30.1
Benzophenone-4	72.8	3.0	52.7	5.0	38.9	5.0	92.6	33.0	122.8	5.0	101.9	5.0
Methylparaben	70.2	27.1	59.4	27.4	39.9	37.5	96.3	1.6	100.8	3.3	99.8	5.0
Ethylparaben	70.1	0.5	73.0	5.1	68.5	0.8	77.5	6.2	83.5	3.8	87.5	8.7
Propylparaben	69.8	5.8	75.1	0.7	55.6	4.2	117.4	3.8	125.3	7.8	112.6	5.0
Butylparaben	76.8	0.1	82.3	1.8	47.9	13.9	115.4	9.6	118.3	2.6	102.4	2.0
Bisphenol A	60.5	3.9	56.8	22.0	37.4	47.1	108.7	14.0	100.7	8.2	112.2	22.3
E1	63.2	1.7	64.3	3.3	50.6	7.3	97.2	3.6	92.7	2.7	99.1	5.0
E2	52.9	23.0	67.5	10.9	51.5	15.1	79.3	26.3	87.9	11.4	106.6	13.0
EE2	48.3	6.1	64.9	11.0	51.9	13.1	86.3	5.4	84.6	7.0	87.9	12.0
Sulfasalazine	31.8	6.4	28.4	0.2	21.8	3.3	64.2	10.4	58.7	9.8	62.7	0.4
Clarithromycin	31.0	33.9	40.1	1.2	33.2	20.4	77.6	2.4	97.2	7.7	80.9	0.5
Azithromycin	21.9	11.4	24.5	6.3	22.2	12.4	86.9	17.0	67.0	11.3	101.1	29.1
Trimethoprim	59.2	27.5	59.8	0.8	41.4	6.6	118.0	18.3	123.7	5.4	109.6	17.1
Sulfamethoxazole	78.9	8.5	64.0	14.5	42.1	36.5	108.4	11.0	111.0	13.4	102.8	42.3
Triclosan	58.3	5.0	75.1	5.0	69.3	5.0	86.5	5.0	100.8	5.0	117.5	5.0
Amoxicillin	0.4	1.2	30.1	23.1	-	-	-	-	72.3	5.1	-	-
Metronidazole	63.3	6.9	56.3	10.1	51.8	11.0	107.3	8.0	92.3	14.2	110.2	7.9
Sulfadiazine	56.0	3.7	28.2	3.2	23.2	4.1	95.4	8.1	59.0	11.4	56.6	8.7
Cefalexin	30.7	N/A	429.0	413.2	-36.0	135.4	67.2	-	-	-	140.6	5.2
Ofloxacin	66.3	7.4	50.9	12.1	56.7	7.5	97.9	5.1	95.2	7.9	118.3	16.1
Ciprofloxacin	28.2	6.6	28.1	3.4	52.3	38.6	95.4	15.2	69.2	6.9	101.3	7.6
Tetracycline	18.0	14.5	12.3	24.9	14.0	26.9	63.7	11.3	63.0	12.0	108.0	10.2
Danofloxacin	29.6	6.2	30.4	4.7	30.3	2.9	99.9	6.1	71.0	16.1	87.3	9.7

Compound	Absolute						Corrected					
	Surface water		Effluent		Influent		Surface water		Effluent		Influent	
	Rec (%)	SD	Rec (%)	SD	Rec (%)	SD	Rec (%)	SD	Rec (%)	SD	Rec (%)	SD
Oxytetracycline	39.3	6.4	42.3	14.1	101.3	133.7	58.6	6.5	70.3	26.0	85.6	22.1
Chloramphenicol	131.4	119.6	26.6	35.9	85.9	23.8	82.0	6.8	80.0	16.6	123.8	39.7
Penicillin G	6.2	3.6	7.3	1.3	3.9	5.1	12.3	0.3	-	-	-	-
Penicillin V	110.1	22.7	105.5	8.1	25.1	27.8	130.7	23.3	158.7	12.5	60.6	36.1
Erythromycin	923.8	52.9	858.0	18.4	930.1	20.7	84.8	6.0	82.9	4.3	87.7	0.5
Prulifloxacin	1.9	3.5	29.2	15.1	44.7	10.7	-	-	47.5	16.9	69.1	7.8
Norfloxacin	44.7	7.3	48.4	7.4	42.5	5.5	64.8	22.8	83.9	22.9	77.9	15.9
Griseofulvin	98.2	5.9	89.9	2.3	84.8	1.9	122.9	18.6	149.0	18.8	131.3	15.4
Ketoconazole	8.9	5.9	67.2	51.7	67.9	40.3	41.4	23.7	167.8	39.5	141.2	30.8
Valsartan	60.6	4.1	53.1	4.4	46.9	9.1	76.0	34.3	79.9	36.4	101.1	22.4
Irbesartan	56.2	8.8	53.6	10.1	40.3	12.2	112.3	13.8	107.4	17.3	97.6	13.5
Lisinopril	21.4	33.2	21.9	4.3	28.6	61.0	93.7	9.5	88.5	8.8	102.3	45.2
Ketoprofen	72.9	1.1	67.8	2.0	45.6	0.0	92.0	5.2	109.2	24.5	107.2	3.8
Ibuprofen	85.6	5.6	126.2	18.4	57.9	5.0	115.5	27.6	110.8	18.3	78.9	5.0
Naproxen	80.5	2.9	84.5	5.0	15.7	5.0	91.0	6.6	98.0	5.0	82.7	5.0
Diclofenac	70.5	4.2	71.8	5.8	46.5	33.4	120.0	12.7	116.0	1.1	103.9	9.9
Acetaminophen	44.4	5.0	44.8	5.0	77.8	10.4	140.5	5.0	116.4	5.0	77.8	45.9
Bezafibrate	73.7	4.3	78.3	18.7	46.3	5.0	94.2	12.1	92.9	0.7	91.4	5.0
Atorvastatin	35.5	15.9	59.9	2.2	58.6	8.4	54.7	14.9	91.9	2.2	94.0	7.2
Gemfibrozil	177.3	49.3	129.5	28.1	66.6	12.7	176.0	42.1	167.5	16.2	94.3	15.5
Candesartan Cilexetil	32.0	7.8	289.8	149.1	401.6	59.5	114.9	20.4	-	-	-	-
Fexofenadine	67.6	5.5	71.0	22.9	50.7	17.2	74.9	13.4	83.8	26.7	107.3	41.9
Cetirizine	47.9	50.2	78.8	15.2	48.6	36.0	77.3	44.2	114.3	13.2	77.3	36.2
Sildenafil	46.9	12.9	79.3	39.3	70.8	26.0	111.1	7.4	116.2	17.2	128.3	7.5
Metformin	55.2	9.2	53.0	9.2	36.7	23.5	93.3	14.4	96.0	3.3	78.7	38.7
Gliclazide	32.9	0.8	61.8	7.4	46.4	12.6	69.5	31.3	108.4	2.9	80.1	18.5
Sitagliptin	38.3	3.1	36.2	3.8	35.1	5.6	125.5	36.8	84.9	19.1	104.3	14.3
Pholcodine	76.9	5.0	43.1	5.0	13.7	5.0	119.6	5.0	102.0	5.0	42.0	5.0

Compound	Absolute						Corrected					
	Surface water		Effluent		Influent		Surface water		Effluent		Influent	
	Rec (%)	SD	Rec (%)	SD	Rec (%)	SD	Rec (%)	SD	Rec (%)	SD	Rec (%)	SD
Atenolol	76.0	2.0	54.6	5.0	42.8	5.0	115.1	6.1	109.7	5.0	88.6	5.0
Metoprolol	71.8	7.6	53.1	15.0	36.2	16.7	107.8	2.9	104.3	12.9	97.4	12.7
Propranolol	45.4	22.9	36.1	18.9	38.7	12.8	107.2	5.9	102.7	4.8	105.0	9.7
Bisoprolol	73.0	5.9	51.8	7.0	54.3	10.4	167.9	28.8	95.4	33.9	123.4	36.4
Ranitidine	65.0	16.4	46.4	5.0	70.1	72.9	88.6	13.8	106.8	5.0	97.8	35.4
Cimetidine	32.7	7.4	33.4	5.0	20.6	41.0	95.3	5.5	106.2	26.3	123.7	9.7
Iopromide	97.0	11.2	82.1	12.4	47.2	10.1	143.0	33.9	123.7	24.9	138.5	38.7
Buprenorphine	30.6	3.7	39.6	5.7	33.3	10.4	209.6	1.0	228.8	34.9	135.8	11.2
Ephedrine/pseudoephedrine	25.4	15.1	18.7	51.4	23.0	12.3	67.8	14.5	88.6	4.7	92.8	9.2
Norephedrine	28.4	5.0	28.9	5.0	26.9	5.0	78.2	5.0	100.9	5.0	85.4	5.0
Azathioprine	88.9	29.7	81.5	33.9	72.0	25.6	66.4	8.7	83.8	15.7	109.5	3.2
Methotrexate	22.6	11.1	30.7	29.0	39.1	109.1	95.7	6.3	114.1	15.2	98.8	37.8
Ifosfamide	62.9	24.6	41.9	30.2	33.4	24.6	90.8	18.5	80.2	31.4	89.2	22.4
Tamoxifen	0.2	5.3	8.7	57.5	9.5	43.8	112.8	10.2	98.4	25.3	85.2	27.4
Imatinib	4.8	0.6	44.5	37.7	-12.8	41.0	47.2	4.1	73.6	6.1	46.6	3.2
Capecitabine	62.3	8.8	70.4	6.0	60.4	2.7	73.3	10.5	111.5	19.3	104.8	20.6
Bicalutamide	91.8	32.0	108.2	36.7	104.7	27.6	71.2	24.3	99.8	33.9	95.2	32.9
Ketamine	68.2	7.5	53.8	10.8	41.7	10.9	107.7	0.5	115.6	12.4	111.6	3.2
Norketamine	65.7	17.6	53.9	22.0	42.2	21.2	103.2	1.9	108.6	6.8	98.9	1.8
Venlafaxine	58.8	23.2	36.3	19.4	23.8	3.1	96.8	5.7	89.5	22.1	70.2	5.8
Desmethylvenlafaxine	62.5	24.2	45.9	5.0	35.9	5.0	107.4	0.1	100.0	5.0	101.5	5.0
Fluoxetine	4.4	31.8	7.1	5.5	19.8	6.1	112.3	1.0	111.1	17.6	110.7	13.3
Norfluoxetine	3.0	42.2	7.9	4.3	23.6	1.5	74.4	25.9	91.7	3.6	94.8	19.0
Sertraline	3.1	13.4	8.3	29.5	13.4	6.0	108.0	2.9	105.0	13.8	91.5	8.1
Mirtazapine	57.5	30.1	39.8	26.1	25.8	34.1	99.1	20.6	97.0	25.7	84.7	26.8
Citalopram	41.1	28.8	35.5	5.0	40.3	4.7	115.0	11.6	124.1	5.0	119.9	14.9
Desmethylcitalopram	36.2	5.0	27.5	25.6	32.5	35.1	89.5	5.0	110.9	0.9	93.1	15.6

Compound	Absolute						Corrected					
	Surface water		Effluent		Influent		Surface water		Effluent		Influent	
	Rec (%)	SD	Rec (%)	SD	Rec (%)	SD	Rec (%)	SD	Rec (%)	SD	Rec (%)	SD
Paroxetine	4.4	1.5	9.8	1.8	19.9	3.3	29.1	3.5	49.7	4.9	76.2	4.7
Duloxetine	5.1	3.3	8.5	4.5	18.7	8.3	39.8	7.7	55.3	7.6	83.2	4.7
Amitriptyline	17.6	5.5	20.7	3.1	25.7	6.7	96.7	5.4	97.5	13.2	104.5	11.1
Nortriptyline	12.6	3.6	16.1	3.1	23.4	5.7	98.4	14.9	103.7	17.8	108.0	17.5
Norsertraline	-	-	-		31.8	13.3	-	-	-	-	62.9	4.0
Carbamazepine	68.2	9.7	55.5	13.0	37.4	9.3	101.7	7.0	102.5	13.8	88.3	11.1
Carbamazepine 10,11-epoxide	94.8	5.0	55.0	5.0	56.7	5.0	136.1	5.0	126.9	5.0	143.7	5.0
10,11-Dihydro -10-hydroxycarbamazepine	74.1	13.8	59.5	26.4	50.3	30.2	125.1	4.7	120.6	3.7	152.2	0.1
Diltiazem	43.7	28.2	30.1	24.0	36.3	16.1	82.9	0.0	82.3	15.7	118.1	9.1
Verapamil	25.0	8.2	34.0	4.5	31.8	5.3	95.1	4.4	102.2	20.0	111.2	16.7
Temazepam	66.3	15.5	72.5	0.3	54.5	9.3	115.3	8.5	113.9	7.0	107.3	8.5
Oxazepam	92.2	9.1	82.6	9.8	75.1	3.7	105.1	11.2	105.5	17.0	115.0	12.7
Diazepam	83.5	6.3	70.5	6.3	70.8	2.7	92.4	11.5	85.8	10.2	95.6	9.9
Quetiapine	51.6	28.9	46.8	23.5	37.8	25.4	87.2	21.2	95.1	18.6	72.6	18.7
Risperidone	35.3	6.1	38.1	1.9	30.3	3.8	171.6	19.6	119.9	12.2	116.1	9.4
Donepezil	42.7	8.5	42.1	4.3	41.6	8.6	158.0	17.7	112.9	15.5	117.3	8.4
Memantine	58.6	6.8	42.5	8.0	35.6	8.9	181.6	28.8	139.0	24.8	127.8	10.2
Creatinine	31.0	10.9	24.8	13.0	22.8	17.0	59.6	0.6	42.9	11.8	46.9	44.7
Nicotine	45.3	4.8	55.7	15.1	98.5	11.7	54.9	39.6	83.9	56.1	77.3	61.8
Caffeine	137.0	40.8	89.8	5.0	82.9	11.1	122.8	5.7	131.7	5.0	96.6	15.4
Cotinine	72.0	19.8	47.3	26.8	37.4	5.0	105.9	2.1	101.5	13.4	85.2	5.0
1,7-dimethylxanthine	47.5	5.0	26.6	5.0	89.3	2.1	75.5	5.0	110.6	5.0	79.6	59.3
Morphine	57.1	14.8	47.8	0.9	34.2	6.0	112.5	8.7	109.8	4.7	93.9	10.4
Dihydromorphine	45.4	32.8	31.4	28.1	19.9	42.9	92.8	4.7	96.2	2.8	95.7	25.2
Normorphine	42.8	24.4	38.6	28.6	30.3	30.1	87.3	5.4	106.5	8.6	109.5	3.2
Methadone	46.5	15.0	47.9	6.9	49.6	10.6	119.2	14.9	111.7	13.0	109.0	0.5
EDDP	23.8	32.8	33.9	10.7	44.1	2.0	105.2	1.0	113.6	0.4	111.6	2.9

	Absolute						Corrected					
	Surface water		Effluent		Influent		Surface water		Effluent		Influent	
Compound	Rec (%)	SD	Rec (%)	SD	Rec (%)	SD	Rec (%)	SD	Rec (%)	SD	Rec (%)	SD
Codeine	67.5	10.6	68.4	56.6	39.0	5.0	93.1	16.2	102.8	8.3	94.8	5.0
Norcodeine	52.5	11.1	36.4	21.2	35.5	10.1	90.6	2.4	87.5	32.5	95.7	11.5
Dihydrocodeine	66.4	6.7	54.8	34.0	34.7	13.9	98.5	2.6	118.6	19.7	85.7	9.9
Tramadol	60.9	24.0	47.0	4.1	33.3	10.8	104.8	0.5	88.3	27.3	69.5	64.8
N-desmethyltramadol	42.2	5.0	33.4	15.0	17.9	5.0	69.1	5.0	98.8	0.7	81.3	5.0
O-desmethyltramadol	58.6	19.1	36.0	26.6	31.8	27.6	108.1	21.9	93.9	6.5	87.3	17.3
Amphetamine	22.5	38.1	27.4	23.9	24.5	15.3	98.7	16.7	107.8	11.2	91.7	18.5
Methamphetamine	47.7	4.4	42.5	15.5	31.9	8.2	97.6	1.4	97.1	12.1	102.1	2.4
MDMA	50.0	11.3	37.0	13.2	29.4	10.8	100.9	1.9	112.9	18.5	118.5	18.7
MDA	28.7	18.9	30.3	19.0	30.7	21.1	99.6	1.1	99.1	13.0	99.4	3.8
Cocaine	71.4	5.5	44.9	9.2	21.7	33.0	106.9	2.5	105.2	2.5	97.4	15.3
Benzoylecgonine	74.2	15.2	55.1	14.6	46.5	3.2	126.2	6.3	116.4	10.8	106.3	2.3
Anhydroecgoninemethylester	53.6	0.6	50.2	10.3	33.9	5.0	79.5	5.8	109.5	13.3	91.3	8.0
Cocaethylene	71.3	7.8	48.1	14.9	7.6	40.9	105.9	2.6	107.4	15.7	109.9	4.8
Mephedrone	22.8	12.9	22.8	11.8	18.2	25.1	97.3	5.6	103.7	0.3	94.8	10.6
MDPV	112.2	17.7	85.5	14.7	20.8	33.8	78.9	20.8	87.9	21.6	136.8	0.2
Heroin	54.1	20.3	29.1	18.3	23.9	16.0	100.9	7.1	103.0	1.2	105.3	7.6
6-acetylmorphine	52.9	27.4	40.0	30.9	33.9	26.6	72.6	31.8	85.4	32.5	103.6	3.1
Thiamethoxam	53.3	11.3	28.3	3.1	23.9	3.5	70.8	1.7	41.0	2.2	34.1	1.3
Imidacloprid	92.7	10.3	62.8	11.8	60.7	15.2	118.6	2.9	107.4	6.0	107.9	9.3
Clothianidin	76.5	7.5	63.1	9.0	64.0	10.5	111.1	18.7	89.5	16.6	83.4	18.4
Metazachlor	85.7	4.4	64.7	6.7	64.7	7.4	107.3	7.7	96.4	8.9	108.5	5.5
Terbuthylazine	72.9	3.4	64.1	2.5	56.2	2.8	83.2	7.2	79.8	9.3	75.7	10.8
Methiocarb	79.4	6.9	62.0	4.5	69.1	2.4	97.7	3.8	91.3	7.6	97.1	3.4
Dichlofluanid	23.3	9.6	2.3	2.9	12.6	3.4	-	-	-	-	51.3	16.9
Flufenacet	82.7	10.5	69.7	9.2	62.9	4.3	82.9	11.5	87.9	12.8	87.9	10.2
Oxadiazon	63.9	21.8	60.6	9.8	52.6	6.2	78.9	19.8	91.5	11.3	79.1	8.1

	Absolute						Corrected					
	Surface water		Effluent		Influent		Surface water		Effluent		Influent	
**Compound**	**Rec (%)**	**SD**	**Rec (%)**	**SD**	**Rec (%)**	**SD**	**Rec (%)**	**SD**	**Rec (%)**	**SD**	**Rec (%)**	**SD**
Chlorpyrifos	15.1	5.1	42.2	N/A	138.0	46.3	21.8	5.9	65.7	-	-	-
Triallate	32.2	9.4	28.7	6.4	73.2	N/A	45.9	6.0	49.6	4.8	106.7	-
Tylosin	43.8	32.2	50.3	16.6	34.3	13.9	100.4	13.4	110.0	22.6	98.3	0.3
Sulfapyridine	76.8	4.7	50.0	5.6	47.1	5.9	101.8	21.1	75.0	1.5	84.3	20.8
Sarafloxacin	37.3	6.6	30.5	8.8	36.3	7.9	134.0	45.5	84.1	39.4	110.9	36.3
Ceftiofur	11.0	8.9	30.2	7.0	39.1	14.4	19.4	17.5	63.6	18.6	82.8	30.5
Diazinon	74.8	17.6	67.8	15.0	60.7	14.1	75.2	11.9	72.5	13.0	81.6	18.0

Table S9 Solid matrix absolute and corrected recoveries

	Absolute				Corrected			
	SPM		Digested Solids		SPM		Digested Solids	
**Compound**	**Rec (%)**	**SD**	**Rec (%)**	**SD**	**Rec (%)**	**SD**	**Rec (%)**	**SD**
Benzophenone-1	41.4	8.4	8.1	2.2	120.0	9.1	69.7	58.6
Benzophenone-2	34.3	2.8	11.1	0.8	141.3	9.7	105.5	19.0
Benzophenone-3	-	-	-	-	-	-	-	-
Benzophenone-4	17.5	2.8	7.6	1.8	72.6	5.5	47.0	9.9
Methylparaben	44.2	7.7	18.1	11.9	149.1	22.4	110.3	81.5
Ethylparaben	40.2	7.8	19.5	0.7	109.5	26.7	118.7	13.0
Propylparaben	49.5	4.1	16.0	0.6	200.4	14.6	153.6	30.8
Butylparaben	36.8	2.8	11.2	0.7	247.0	10.4	179.6	14.3
Bisphenol A	35.7	10.0	11.9	-	58.8	12.0	95.7	-
E1	16.2	1.9	5.9	2.1	115.3	8.5	106.6	28.0
E2	14.0	1.5	6.4	0.8	109.4	12.6	113.6	9.9
EE2	-	-	-	-	-	-	-	-
Sulfasalazine	-	-	-	-	-	-	-	-
Clarithromycin	-	-	-	-	-	-	-	-
Azithromycin	7.6	6.0	14.0	12.0	22.4	4.8	60.5	17.7
Trimethoprim	69.3	17.2	44.9	2.5	146.0	30.9	138.5	6.6
Sulfamethoxazole	36.1	2.9	24.4	2.4	62.7	2.3	74.4	5.8
Triclosan	-	-	-	-	-	-	-	-
Amoxicillin	22.9	18.7	26.3	25.2	-	-	-	-
Metronidazole	8.0	1.6	5.6	0.3	121.2	18.4	103.1	6.9
Sulfadiazine	9.9	1.7	-	-	151.6	19.3	166.0	36.4
Cefalexin	-	-	-	-	-	-	-	-
Ofloxacin	6.3	3.9	-	-	-	-	-	-
Ciprofloxacin	9.3	3.7	0.9	1.0	-	-	-	-
Tetracycline	-	-	12.5		-	-	-	-
Danofloxacin	-	-	0.3	0.4	-	-	5.6	0.7
Oxytetracycline	-	-	-	-	-	-	-	-
Chloramphenicol	26.3	2.3	17.2	3.5	125.3	9.2	179.5	19.5
Penicillin G	-	-	-	-	-	-	-	-
Penicillin V	1.9	0.7	29.0	18.4	8.7	2.3	-	-
Erythromycin	-	-	-	-	-	-	-	-
Prulifloxacin	-0.8		-41.8	22.9	156.0		-	-
Norfloxacin	-	-	-	-	-	-	-	-
Griseofulvin	17.8	3.1	6.6	1.2	82.0	10.1	60.6	9.5
Ketoconazole	8.5	1.7	-3.2	18.3	13.3	1.8	100.6	17.8
Valsartan	-	-	-	-	-	-	-	-
Irbesartan	-	-	-	-	-	-	-	-
Lisinopril	67.6	6.7	37.7	2.9	109.1	10.9	127.8	14.5
Ketoprofen	28.9	1.0	23.1	3.8	98.2	5.0	119.4	8.6
Ibuprofen	42.3	13.9	14.9	4.0	101.0	7.8	105.5	9.6
Naproxen	44.1	9.9	16.4	2.3	96.5	14.6	107.9	9.6
Diclofenac	41.9	12.3	4.2	1.7	88.3	13.9	40.8	14.6
Acetaminophen	14.8	3.6	3.9	0.5	129.8	25.8	57.7	10.8

Compound	Absolute				Corrected			
	SPM		Digested Solids		SPM		Digested Solids	
	Rec (%)	SD	Rec (%)	SD	Rec (%)	SD	Rec (%)	SD
Bezafibrate	32.3	2.5	16.4	2.2	98.0	8.7	112.5	5.5
Atorvastatin	-	-	-	-	-	-	-	-
Gemfibrozil	7.5	3.9	2.9	0.3		-	26.3	1.6
Candesartan Cilexetil	-	-	-	-	-	-	-	-
Fexofenadine	-	-	-	-	-	-	-	-
Cetirizine	-	-	-	-	-	-	-	-
Sildenafil	35.6	2.2	26.2	1.5	110.9	6.4	116.7	4.9
Metformin	-	-	-	-	-	-	-	-
Gliclazide	-	-	-	-	-	-	-	-
Sitagliptin	42.0	3.7	30.0	3.8	79.4	7.9	99.2	8.1
Pholcodine	29.7	2.6	22.8	2.7	62.0	4.2	87.8	11.0
Atenolol	52.1	4.6	30.8	2.7	105.2	9.4	121.0	10.8
Metoprolol	57.7	2.1	36.6	1.6	95.1	3.9	99.7	4.9
Propranolol	42.7	5.6	20.9	4.8	103.3	8.4	91.5	14.0
Bisoprolol	38.1	5.9	26.6	3.8	125.8	9.9	126.1	8.6
Ranitidine	61.7	8.3	21.5	1.9	117.8	17.8	82.1	3.6
Cimetidine	-	-	-	-	-	-	-	-
Iopromide	-	-	-	-	-	-	-	-
Buprenorphine	18.1	3.6	12.4	2.0	59.9	9.4	88.0	8.5
Ephedrine/pseudoephedrine	40.0	3.1	28.2	5.2	72.9	2.8	97.9	21.0
Norephedrine	37.2	3.9	27.0	5.6	64.0	5.1	92.6	18.9
Azathioprine	-	-	-	-	-	-	-	-
Methotrexate	48.2	1.5	16.9	2.6	87.1	2.2	59.3	8.7
Ifosfamide	-	-	-	-	-	-	-	-
Tamoxifen	15.9	9.5	0.3	0.0	118.7	49.7	112.7	7.0
Imatinib	9.0	2.1	10.5	2.4	39.9	8.4	65.3	10.8
Capecitabine	4.8	2.5	5.0	1.0	22.1	0.5	53.4	5.8
Bicalutamide	23.0	3.5	21.8	4.0	87.0	8.3	176.0	30.6
Ketamine	48.5	3.3	29.0	0.9	101.7	4.3	105.3	4.3
Norketamine	41.8	5.4	30.3	1.6	96.3	3.6	109.7	4.3
Venlafaxine	40.7	10.1	11.3	22.5	70.3	20.8	61.9	6.4
Desmethylvenlafaxine	58.2	5.2	33.9	3.8	104.3	5.6	96.9	10.3
Fluoxetine	28.2	1.6	9.4	2.7	104.1	0.9	121.2	48.4
Norfluoxetine	34.7	6.0	7.4	0.9	120.3	18.5	95.1	37.5
Sertraline	72.1	98.5	5.8	6.2	-	-	101.9	14.8
Mirtazapine	33.0	2.0	18.5	2.3	89.2	4.6	98.2	9.9
Citalopram	46.4	2.5	30.4	5.1	105.3	17.7	112.7	37.3
Desmethylcitalopram	41.5	3.0	20.8	3.0	96.4	10.6	82.2	18.8
Paroxetine	18.3	2.5	15.8	3.4	83.5	6.1	103.0	15.6
Duloxetine	10.2	1.8	9.2	2.1	48.3	6.8	61.3	9.3
Amitriptyline	27.9	3.5	8.9	9.1	72.0	9.2	175.2	39.5
Nortriptyline	27.3	1.7	16.5	3.7	104.8	4.0	194.4	1.8
Norsertraline	22.0	32.0	19.2	12.2	39.7	2.7		
Carbamazepine	30.7	1.9	10.8	0.9	98.7	3.3	94.5	13.9

	Absolute				Corrected			
	SPM		Digested Solids		SPM		Digested Solids	
**Compound**	**Rec (%)**	**SD**	**Rec (%)**	**SD**	**Rec (%)**	**SD**	**Rec (%)**	**SD**
Carbamazepine 10,11-epoxide	-	-	-	-	-	-	-	-
10,11-Dihydro -10-hydroxycarbamazepine	31.6	3.5	11.5	0.6	101.6	8.3	100.1	2.8
Diltiazem	-	-	-	-	-	-	-	-
Verapamil	46.3	5.9	34.3	2.1	102.5	10.6	130.8	6.7
Temazepam	9.5	2.2	6.1	1.6	58.5	5.5	92.3	6.6
Oxazepam	0.5	N/A	1.4	0.7	115.0		115.1	15.5
Diazepam	24.4	4.0	17.3	2.4	82.2	6.8	99.3	1.7
Quetiapine	51.6	3.5	19.1	3.1	113.9	5.3	81.9	9.0
Risperidone	36.3	7.0	15.7	4.2	75.8	6.6	57.4	12.8
Donepezil	41.6	8.7	26.8	4.2	97.2	11.6	98.3	11.0
Memantine	20.0	1.6	19.6	3.7	36.6	9.2	71.4	10.6
Creatinine	-	-	-	-	-	-	-	-
Nicotine	50.1	7.8	45.6	4.0	92.7	12.5	165.4	24.4
Caffeine	-	-	-	-	-	-	-	-
Cotinine	10.9	1.5	4.1	1.7	102.7	2.8	112.1	11.6
1,7-dimethylxanthine	-	-	-	-	-	-	-	-
Morphine	27.1	3.8	15.8	6.3	137.0	28.6	114.0	30.9
Dihydromorphine	13.2	1.9	11.0	1.2	64.6	3.9	74.3	3.4
Normorphine	25.1	4.3	17.4	4.0	127.2	14.6	109.9	17.2
Methadone	46.3	2.4	29.2	0.7	95.5	3.8	102.8	3.2
EDDP	35.6	3.4	25.5	2.8	98.2	5.7	103.8	9.3
Codeine	58.7	8.3	30.2	1.9	116.5	25.7	105.3	19.0
Norcodeine	39.1	5.3	24.0	1.9	77.9	5.6	87.8	10.8
Dihydrocodeine	44.5	4.4	27.8	2.1	91.1	5.6	101.0	10.4
Tramadol	47.1	3.0	30.7	2.1	80.8	3.6	87.7	6.1
N-desmethyltramadol	44.1	4.5	24.7	1.5	84.3	4.1	83.4	10.7
O-desmethyltramadol	-	-	-	-	-	-	-	-
Amphetamine	60.8	3.7	34.1	2.5	110.7	4.2	118.3	8.7
Methamphetamine	53.4	2.1	33.8	1.8	113.2	5.0	104.4	2.1
MDMA	45.0	2.5	28.1	1.3	96.0	2.6	105.6	1.9
MDA	-	-	-	-	-	-	-	-
Cocaine	52.0	2.3	32.8	1.6	99.2	3.7	104.7	2.9
Benzoylecgonine	54.4	4.4	35.7	1.9	100.4	3.9	117.3	7.3
Anhydroecgoninemethylester	-	-	-	-	-	-	-	-
Cocaethylene	48.5	2.9	30.0	1.3	99.8	3.8	98.9	6.5
Mephedrone	23.3	3.2	15.9	1.5	61.9	3.3	99.4	4.1
MDPV	44.0	4.6	24.6	2.7	88.3	5.2	79.7	8.3
Heroin	27.7	1.8	17.9	2.2	98.7	4.9	91.7	6.5
6-acetylmorphine	-	-	-	-	-	-	-	-
Thiamethoxam	20.1	4.8	15.1	3.5	95.3	20.6	140.7	26.1
Imidacloprid	29.0	7.3	24.2	2.9	103.2	21.4	-	-
Clothianidin	32.6	6.8	29.9	3.0	159.3	27.1	-	-
Metazachlor	26.0	3.4	8.9	1.8	92.7	5.5	96.1	8.0

	Absolute				Corrected			
	SPM		Digested Solids		SPM		Digested Solids	
**Compound**	**Rec (%)**	**SD**	**Rec (%)**	**SD**	**Rec (%)**	**SD**	**Rec (%)**	**SD**
Terbuthylazine	13.3	1.6	8.4	1.9	45.3	7.6	47.4	4.9
Methiocarb	22.3	1.6	12.6	1.1	104.3	5.5	117.5	3.7
Dichlofluanid	-	-	0.0	0.2	-	-	-	-
Flufenacet	14.7	2.0	5.6	1.0	45.9	4.6	57.9	10.5
Oxadiazon	3.0	1.1	2.5	0.6	14.8	1.9	25.0	4.0
Chlorpyrifos	4.6	2.4	7.9	1.9	-	-	86.1	20.0
Triallate	2.3	0.9	4.1	1.7	-	-	-	-
Tylosin	-	-	-	-	-	-	-	-
Sulfapyridine	26.5	5.1	23.5	4.5	-	-	-	-
Sarafloxacin	6.3	2.8	-	-	-	-	-	-
Ceftiofur	-	-	-	-	-	-	-	-
Diazinon	0.5	0.3	1.1	0.5	145.8	4.5	80.8	9.3

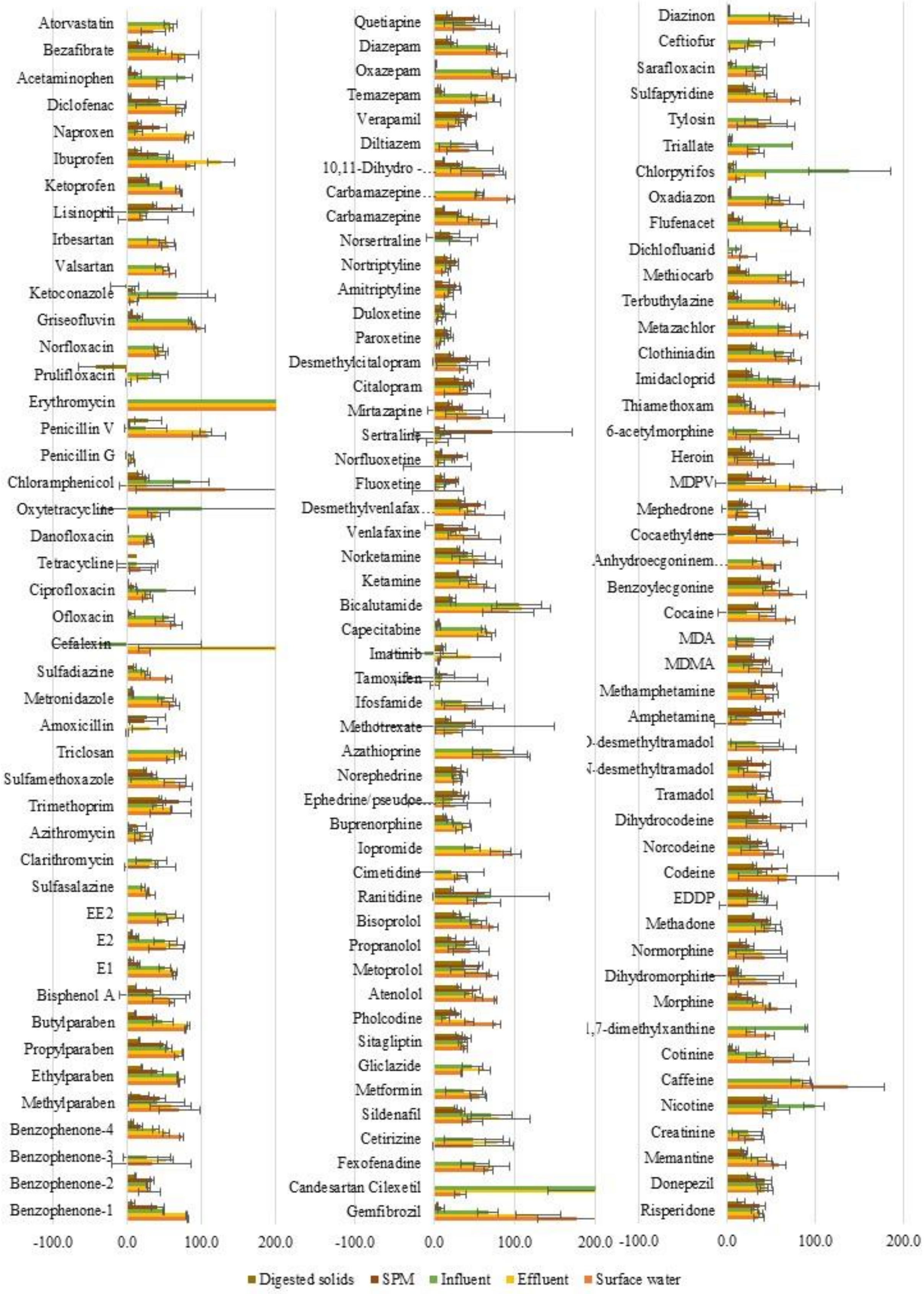

Fig. S2 Absolute recoveries for all matrices, error bars indicate the range of standard deviation

Table S10 Matrix suppression

Compound	Surface water (%)	SD	Effluent (%)	SD	Influent (%)	SD
Benzophenone-1	0.6	2	2	4.5	30.6	0.1
Benzophenone-2	43.8	1.1	49.2	1.9	55.5	1
Benzophenone-3	-5.1	1	7.7	3.3	20.7	1.1
Benzophenone-4	-1.2	9.4	23	6.8	42.5	1.7
Methylparaben	41.9	3.6	49.1	1.5	60.2	1.4
Ethylparaben	27.8	3.4	29.8	0.6	37	2.9
Propylparaben	21.7	4.3	21.6	2.1	37.3	4.2
Butylparaben	8	1.9	11	1.4	35.9	2.4
Bisphenol A	15	0.2	12.4	2.6	29.4	2.9
E1	4.5	4.3	5	1.1	28.6	1.4
E2	8.3	8.1	8	5.9	31.8	0.9
EE2	-6.1	1.7	-24.4	3.9	13.2	4.8
Sulfasalazine	-105.8	9.9	-81.7	3.1	-53.4	5.1
Clarithromycin	52	1.6	54.1	0.5	54	1.2
Azithromycin	72.7	0.6	73.9	1.9	68.8	3.9
Trimethoprim	38.2	0.8	51	2.7	49.1	4.5
Sulfamethoxazole	28.4	0.4	38.1	1.5	47.6	0.8
Triclosan	-62.3	0.3	-100.9	3.1	-99	4.8
Amoxicillin	99.8	0.7	93.9	0.7	99.0	0.7
Metronidazole	24.5	1.3	47.4	5.9	51.3	1.5
Sulfadiazine	37.1	1.1	64.6	1.8	71.1	1.1
Cefalexin	37.7	4.8	28.4	12.9	31.5	5.6
Ofloxacin	29.3	9.9	40.2	3.3	48.4	3.3
Ciprofloxacin	67.8	5.4	69.5	1.0	74.1	3.5
Tetracycline	6.5	1.3	27.3	0.9	36.7	9.0
Danofloxacin	70.2	5.7	73.7	2.7	81.3	3.8
Oxytetracycline	13.9	0.8	29.3	3.4	22.5	22.6
Chloramphenicol	33.4	2.4	39.3	1.2	45.7	3.3
Penicillin G	101.1	0.1	100.5	0.2	96.7	0.8
Penicillin V	-15.4	10.8	-13.1	33.7	-2.5	6.9
Erythromycin	36.5	11.2	-195.6	14.7	57.8	6.8
Prulifloxacin	107.9	1.9	87.4	18.7	44.9	26.5
Norfloxacin	52.7	3.9	62.2	3.1	61.5	19.3
Griseofulvin	4.3	0.5	9.9	2.0	17.3	4.4
Ketoconazole	90.4	4.2	44.6	0.1	38.0	3.0
Valsartan	-139.2	2.6	-105.4	5.9	-77.4	5.7
Irbesartan	-23.8	1.2	-25.3	1.3	-14.3	0.2
Lisinopril	-12.3	2.5	8.6	0.5	23.1	0
Ketoprofen	8.5	0.2	9.9	3.2	36.3	1.5
Ibuprofen	-13.6	4.4	-24.3	9.9	-15.6	5
Naproxen	-13.2	1.6	-18.7	0.7	29.8	34.1
Diclofenac	-127.3	0.8	-141.7	5.7	-104.5	2.2
Acetaminophen	27.8	1.9	50.6	0.6	22.2	19
Bezafibrate	-24.5	10.8	-19.6	3.7	0.7	0.5

	Surface water		Effluent		Influent	
**Compound**	**(%)**	**SD**	**(%)**	**SD**	**(%)**	**SD**
Atorvastatin	-155.7	2.9	-160.3	1.4	-152.8	14.5
Gemfibrozil	-70.3	20.5	-64.4	1.9	29.5	1.0
Candesartan Cilexetil	100.0	23.2	-108.3	37.4	-78.1	47.6
Fexofenadine	10.3	1.4	11.2	1.2	27.1	1.9
Cetirizine	16.9	3	18	0.1	30.5	0.4
Sildenafil	67.2	1.6	32.4	4.7	47.3	1.9
Metformin	44.8	1	47	1.2	63.3	0.6
Gliclazide	19	4.6	23.6	1.1	38.2	1.3
Sitagliptin	23.8	0.0	30.2	0.5	38.4	1.1
Pholcodine	11.4	2.3	45.5	0.6	90	1.4
Atenolol	22.3	1.1	48	0.7	59.7	1.6
Metoprolol	27.6	0.5	49.5	0.1	65.7	0.1
Propranolol	31.2	3.7	59	1.4	56.7	0.6
Bisoprolol	10.5	0.0	37.8	4.0	32.2	1.2
Ranitidine	18.2	4	35	0.8	56.8	1.1
Cimetidine	23.4	4.7	48.4	0.8	61.9	1.6
Iopromide	11.8	2.7	26.1	3.5	39.1	0
Buprenorphine	52.0	2.5	34.1	3.4	45.7	4.3
Ephedrine/pseudoephedrin e	36.5	1.6	59.8	0.1	67.7	0.1
Norephedrine	18.2	2.6	38.4	1.2	41.3	1
Azathioprine	32.8	0.9	45	3.1	51.1	0.9
Methotrexate	43.2	1.8	52.3	0.2	51.1	1.1
Ifosfamide	26.1	1	46.7	0.7	55.9	0.8
Tamoxifen	93.2	1.3	14.6	5.3	30.3	3.2
Imatinib	93.3	1.1	86.4	1.5	88.1	0.1
Capecitabine	3.4	4.0	15.3	1.2	23.8	0.7
Bicalutamide	8.8	10.2	-22.8	1.8	-16.3	1.9
Ketamine	25.8	0.2	42.7	0.7	51.5	0.4
Norketamine	21.6	1.8	34.6	0.9	45.4	0.6
Venlafaxine	27.5	2.7	56.5	1	71.5	0.2
Desmethylvenlafaxine	29.9	0.6	59.1	0.6	65.1	4.7
Fluoxetine	75.1	4.7	84.9	0.7	53.9	1.3
Norfluoxetine	79.5	3.4	86.6	0.3	54.8	0.4
Sertraline	88.1	3	88.4	0.5	57.1	0.1
Mirtazapine	26	1.1	50.2	0.7	65.2	0.2
Citalopram	52	2.8	70.1	0.4	63.7	0.8
Desmethylcitalopram	40.6	3.1	65.6	0.1	54.5	0.7
Paroxetine	93.7	0.2	86.7	0.5	70.5	1.6
Duloxetine	90.5	0.7	83.4	0.2	60.2	3.9
Amitriptyline	78.9	1.4	71.2	0.0	61.4	1.5
Nortriptyline	84.1	1.4	77.3	1.6	62.5	2.8
Norsertraline	98.5	0.3	84.2	1.8	52.9	1.5
Carbamazepine	61.4	2.5	46.6	0.5	61.4	0.4
Carbamazepine 10,11-epoxide	18.3	1.7	32.2	0.5	41.3	1.8

Compound	Surface water (%)	SD	Effluent (%)	SD	Influent (%)	SD
10,11-Dihydro -10-hydroxycarbamazepine	20.3	1.6	33.2	0.2	37.3	0.2
Diltiazem	37.9	4.3	53.6	0.1	61.4	3.6
Verapamil	65.7	1.3	51.9	2.1	57.3	4.6
Temazepam	14.4	0.5	22.3	2	36	0.3
Oxazepam	7.2	0.8	9.6	1.5	21.8	3.0
Diazepam	14.2	1.4	20.2	2.7	25.0	3.0
Quetiapine	35	3.7	50.9	0.8	51.7	0.9
Risperidone	54.7	0.1	58.1	0.1	64.3	1.4
Donepezil	60.3	5.4	53.5	1.4	62.6	1.5
Memantine	38.1	3.1	51.3	2.4	60.8	1.3
Creatinine	69	0.4	75.2	0.1	77.2	0.6
Nicotine	1.2	3.2	5	2.2	47	1.6
Caffeine	22.2	3.2	53.4	8.5	17.1	34.5
Cotinine	33	2.1	55.6	1.4	67.3	0.9
1,7-dimethylxanthine	28.1	0.2	44.9	1.4	10.7	18.1
Morphine	30.1	3.8	47.3	2.3	69	3.3
Dihydromorphine	32.9	1.2	49.9	1.3	67.5	0.1
Normorphine	27.7	7.6	46.8	4.3	60.3	0.7
Methadone	32	3.8	48.4	0.8	45.2	0.3
EDDP	23.4	1.7	45.1	0.2	55.4	1
Codeine	25.9	3.9	53.7	0.5	59.8	1.3
Norcodeine	33.1	1.8	49.1	2.4	53.5	1.3
Dihydrocodeine	21.7	2.8	49.7	0.8	58	2.1
Tramadol	31.8	3.6	54.5	2.2	65.1	0
N-desmethyltramadol	30.2	0.3	55.6	0.5	80.4	0.2
O-desmethyltramadol	34.6	0.2	59.2	1.1	62.5	0.6
Amphetamine	23.6	1.6	36.5	1.2	50.2	0.4
Methamphetamine	23	1.9	44.1	1.6	67	0.2
MDMA	36.3	0.8	58.8	0	69.2	3.1
MDA	29.7	1	44.9	0.7	50.5	1.2
Cocaine	32.7	1.1	49.8	0.2	73.4	0.4
Benzoylecgonine	21.9	5.2	52	0.6	61.6	0.5
Anhydroecgoninemethylester	12.5	0.3	28	1.6	48.4	0.8
Cocaethylene	31.6	1.4	51.8	1	64.3	0.2
Mephedrone	24.6	2.8	44.6	0.3	64.6	0.7
MDPV	22.7	1.1	42.1	0.2	85.6	0.7
Heroin	25	1.6	45.4	0.4	61.4	2.2
6-acetylmorphine	46	2.1	65.1	1.8	66.9	1.7
Thiamethoxam	37.4	2.3	70.7	0.0	75.0	0.0
Imidacloprid	2.3	11.6	33.7	13.0	37.2	8.3
Clothianidin	11.7	1.7	28.7	7.9	25.1	1.6
Metazachlor	14.8	0.8	33.9	1.7	35.4	0.1
Terbuthylazine	36.5	3.4	31.2	2.0	43.0	0.6
Methiocarb	25.4	5.6	25.2	2.0	27.7	1.7

Compound	Surface water (%)	SD	Effluent (%)	SD	Influent (%)	SD
Dichlofluanid	59.7	0.7	94.0	6.3	57.8	6.4
Flufenacet	22.1	0.4	27.3	2.0	36.3	1.1
Oxadiazon	41.8	9.7	19.8	1.6	28.9	2.2
Chlorpyrifos	87.5	5.8	32.8	6.7	-222.8	0.5
Triallate	84.5	1.8	59.4	4.7	-23.1	2.6
Tylosin	31.4	1.1	43.4	0.4	64.9	0.5
Sulfapyridine	26.0	1.4	46.5	2.9	52.5	0.4
Sarafloxacin	66.8	1.4	62.7	3.2	64.5	5.7
Ceftiofur	18.5	28.7	37.0	7.4	10.0	1.8
Diazinon	51.0	4.6	19.6	11.2	14.0	2.6

Table S11 Surface water data (ng L^{-1})

Compound	Day 1 A	Day 1 B	Day 2 A	Day 2 B	Day 3 A	Day 3 B	Day 4 A	Day 4 B	Day 5 A	Day 5 B	Day 6 A	Day 6 B	Day 7 A	Day 7 B
Benzophenone-1	<MQL	<MQL	<MQL	<MQL	<MQL	<MQL	<MQL	<MQL	<MQL	<MQL	<MQL	<MQL	<MQL	<MQL
Benzophenone-2	<MQL	<MQL	<MQL	<MQL	<MQL	<MQL	<MQL	<MQL	<MQL	<MQL	<MQL	<MQL	<MQL	<MQL
Benzophenone-3	21.3	21.5	21	19.2	20.8	21.7	19.7	18.1	17.1	16.4	16.3	15.8	18.7	18.4
Benzophenone-4	476.7	410.3	482	463.4	1339.1	1303.9	878.2	951.3	403	433.7	290.7	280.6	416.1	381.8
Methylparaben	7	6.1	14.3	11.4	4.2	4.3	7.4	7.6	6.4	7	6.2	5.9	5.2	5.5
Ethylparaben	<MQL	<MQL	<MQL	<MQL	<MQL	<MQL	<MQL	<MQL	<MQL	<MQL	<MQL	<MQL	<MQL	<MQL
Propylparaben	5.3	4.5	4.9	3.8	3.2	3.1	3.5	4.9	3.6	3.2	2.5	2.7	3.5	3.7
Butylparaben	<MQL	<MQL	<MQL	<MQL	<MQL	<MQL	<MQL	<MQL	<MQL	<MQL	<MQL	<MQL	<MQL	<MQL
Bisphenol A	75.6	39.6	38.1	27.4	56.5	55.2	22.4	25.9	15.2	15.2	50.4	46.9	39.8	24.7
E1	<MQL	<MQL	<MQL	<MQL	<MQL	<MQL	<MQL	<MQL	<MQL	<MQL	<MQL	<MQL	<MQL	<MQL
E2	<MQL	<MQL	<MQL	<MQL	<MQL	<MQL	<MQL	<MQL	<MQL	<MQL	<MQL	<MQL	<MQL	<MQL
EE2	<MQL	<MQL	<MQL	<MQL	<MQL	<MQL	<MQL	<MQL	<MQL	<MQL	<MQL	<MQL	<MQL	<MQL
Sulfasalazine	49	63.3	58.2	54.9	66.6	57.3	46.9	57.6	54	54.8	47.1	29.6	62.2	48.5
Clarithromycin	115.9	121.3	105.9	98.9	140.1	146.1	105.8	113.5	94.3	93.6	47.4	48.3	98.4	98
Azithromycin	2.5	1	<MQL	<MQL	5.4	5.2	<MQL	0.3	2.5	14.3	<MQL	<MQL	<MQL	<MQL
Trimethoprim	22.8	23.3	23.8	21.8	30.8	25.7	27.8	28.1	25.9	31.2	15.7	15.2	36.3	34.2
Sulfamethoxazole	30	32.2	37.1	36.4	34.6	36	35.8	37.8	34.2	34	29.5	29.6	29	29.1
Triclosan	<MQL	<MQL	<MQL	<MQL	<MQL	<MQL	<MQL	<MQL	<MQL	<MQL	<MQL	<MQL	<MQL	<MQL
Amoxicillin	N/A	N/A	N/A	N/A	N/A	N/A	N/A	N/A	N/A	N/A	N/A	N/A	N/A	N/A
Metronidazole	<MQL	<MQL	<MQL	<MQL	<MQL	<MQL	<MQL	<MQL	<MQL	<MQL	<MQL	<MQL	10.1	<MQL
Sulfadiazine	<MQL	<MQL	<MQL	<MQL	<MQL	<MQL	<MQL	<MQL	<MQL	<MQL	<MQL	<MQL	<MQL	<MQL
Cefalexin	<MQL	<MQL	<MQL	<MQL	<MQL	<MQL	<MQL	<MQL	<MQL	<MQL	<MQL	<MQL	<MQL	<MQL
Ofloxacin	112.8	<MQL	56.5	<MQL	<MQL	<MQL	45.4	<MQL	<MQL	<MQL	<MQL	<MQL	<MQL	<MQL
Ciprofloxacin	<MQL	<MQL	<MQL	<MQL	<MQL	<MQL	<MQL	<MQL	<MQL	<MQL	<MQL	<MQL	<MQL	<MQL
Tetracycline	<MQL	<MQL	<MQL	<MQL	<MQL	<MQL	<MQL	<MQL	<MQL	<MQL	<MQL	<MQL	<MQL	<MQL
Danofloxacin	<MQL	<MQL	<MQL	<MQL	<MQL	<MQL	<MQL	<MQL	<MQL	<MQL	<MQL	<MQL	<MQL	<MQL
Oxytetracycline	<MQL	<MQL	<MQL	<MQL	<MQL	<MQL	<MQL	<MQL	<MQL	<MQL	<MQL	<MQL	<MQL	<MQL

	Day 1		Day 2		Day 3		Day 4		Day 5		Day 6		Day 7	
**Compound**	**A**	**B**	**A**	**B**	**A**	**B**	**A**	**B**	**A**	**B**	**A**	**B**	**A**	**B**
Chloramphenicol	<MQL	<MQL	<MQL	<MQL	<MQL	<MQL	<MQL	<MQL	<MQL	<MQL	<MQL	<MQL	<MQL	<MQL
Penicillin G	<MQL	<MQL	<MQL	<MQL	<MQL	<MQL	<MQL	<MQL	<MQL	<MQL	<MQL	<MQL	<MQL	<MQL
Penicillin V	<MQL	<MQL	<MQL	<MQL	<MQL	<MQL	<MQL	<MQL	<MQL	<MQL	<MQL	<MQL	<MQL	<MQL
Erythromycin	2285	2380.4	2064.4	2080.1	2435.8	2385.3	2083.6	2048.9	2062.6	2059	1849.3	1912.7	2153.7	2274.1
Prulifloxacin	N/A	N/A	N/A	N/A	N/A	N/A	N/A	N/A	N/A	N/A	N/A	N/A	N/A	N/A
Norfloxacin	<MQL	<MQL	<MQL	<MQL	<MQL	<MQL	<MQL	<MQL	<MQL	<MQL	<MQL	<MQL	<MQL	<MQL
Griseofulvin	<MQL	<MQL	<MQL	<MQL	<MQL	<MQL	<MQL	<MQL	<MQL	<MQL	<MQL	<MQL	<MQL	<MQL
Ketoconazole	56.9	<MQL	44.4	<MQL	<MQL	42.8	<MQL	<MQL	<MQL	41.9	<MQL	<MQL	<MQL	<MQL
Valsartan	<MQL	<MQL	<MQL	<MQL	<MQL	<MQL	<MQL	<MQL	<MQL	<MQL	<MQL	<MQL	<MQL	<MQL
Irbesartan	161.1	107.8	98	99.9	117.5	122.2	122.3	126.8	108.8	100.8	84.4	93.5	112.3	104.5
Lisinopril	<MQL	<MQL	<MQL	<MQL	<MQL	<MQL	<MQL	<MQL	<MQL	<MQL	<MQL	<MQL	<MQL	<MQL
Ketoprofen	<MQL	<MQL	<MQL	<MQL	<MQL	<MQL	<MQL	<MQL	<MQL	<MQL	<MQL	<MQL	<MQL	<MQL
Ibuprofen	76	75.7	59.9	57.2	65	75.1	52.7	46.5	34.4	43.4	31.8	31.9	96	101.2
Naproxen	243.9	235.3	216.8	193.5	250.2	230	211.9	221.3	249.3	234.8	154.1	179.1	337	332
Diclofenac	82.3	88.3	85.8	77.6	93.4	93.7	98.1	99.7	87.7	96.2	69.1	81.6	92.4	88.5
Acetaminophen	216	214.6	171.7	142.7	223.5	220.3	204.9	173.1	169.3	167.7	127	104.7	260	306
Bezafibrate	114.2	119.8	95.4	96.8	113.8	119.7	111.6	106.7	93.7	96.2	75.2	69.3	124.6	113.7
Atorvastatin	39.3	48.3	40.7	37.7	52.3	50	48.1	46.8	38.8	44	28.9	31.9	60.9	54.1
Gemfibrozil	<MQL	<MQL	<MQL	<MQL	<MQL	<MQL	<MQL	<MQL	<MQL	<MQL	<MQL	<MQL	<MQL	<MQL
Candesartan Cilexetil	<MQL	<MQL	<MQL	<MQL	<MQL	<MQL	<MQL	<MQL	<MQL	<MQL	<MQL	<MQL	<MQL	<MQL
Fexofenadine	165	240.6	227.1	239.3	225.3	201.6	273.9	253.5	201.8	224.1	135.5	180	225.7	133.7
Cetirizine	188.6	196.3	183.9	205.2	276.8	263.9	268.2	254.1	273.1	297.8	211.1	212.8	269.1	253.1
Sildenafil	2.1	1.1	3.9	3.3	3.7	3.8	1.9	2.1	2.3	4.3	<MQL	0.6	0.6	0.6
Metformin	3750.4	3877.1	3477.7	3526.4	3974.5	3974.5	3594.6	3623.8	3429	3360.8	2727.6	2864	4179.1	4140.1
Gliclazide	51.3	37	36.4	38.6	45	49.9	58.1	54.9	61.2	58.9	44.8	44.7	39.5	42.4
Sitagliptin	170.4	157.2	112.8	153.6	153.3	152.6	150.1	143.8	149.3	142.8	115.3	123.4	128.6	142.2
Pholcodine	<MQL	<MQL	<MQL	<MQL	<MQL	<MQL	<MQL	<MQL	<MQL	<MQL	<MQL	<MQL	<MQL	<MQL
Atenolol	61.7	58.2	49.1	50.4	65.3	60.2	53.3	51.3	48	46.7	32.8	37.5	71.7	68.4
Metoprolol	<MQL	<MQL	<MQL	<MQL	<MQL	<MQL	<MQL	<MQL	<MQL	<MQL	<MQL	<MQL	<MQL	<MQL

	Day 1		Day 2		Day 3		Day 4		Day 5		Day 6		Day 7	
**Compound**	**A**	**B**	**A**	**B**	**A**	**B**	**A**	**B**	**A**	**B**	**A**	**B**	**A**	**B**
Propranolol	23.3	22.4	17.9	19	27.8	25	22.9	21.3	21.3	20.6	15	13.7	19.4	18.8
Bisoprolol	<MQL	<MQL	<MQL	<MQL	<MQL	<MQL	<MQL	<MQL	<MQL	<MQL	<MQL	<MQL	<MQL	<MQL
Ranitidine	135.7	161.6	136.6	140.3	170.9	176.1	174.1	187.7	137.8	179.4	69.2	85.4	150.7	169.3
Cimetidine	<MQL	<MQL	<MQL	<MQL	<MQL	<MQL	<MQL	<MQL	<MQL	<MQL	<MQL	<MQL	<MQL	<MQL
Iopromide	<MQL	<MQL	<MQL	<MQL	<MQL	<MQL	<MQL	<MQL	<MQL	<MQL	<MQL	<MQL	<MQL	<MQL
Buprenorphine	<MQL	6.1	<MQL	6.5	<MQL	<MQL	<MQL	<MQL	<MQL	<MQL	<MQL	<MQL	<MQL	<MQL
Ephedrine/pseudoephedrine	28.3	26.5	24.7	26.2	28.2	26.4	23.6	23.8	24.1	24.5	21.3	23.5	28.2	28.6
Norephedrine	<MQL	<MQL	<MQL	<MQL	<MQL	<MQL	<MQL	<MQL	<MQL	<MQL	<MQL	<MQL	<MQL	<MQL
Azathioprine	<MQL	<MQL	<MQL	<MQL	<MQL	<MQL	<MQL	<MQL	<MQL	<MQL	<MQL	<MQL	<MQL	<MQL
Methotrexate	<MQL	<MQL	<MQL	<MQL	<MQL	<MQL	<MQL	<MQL	<MQL	<MQL	<MQL	<MQL	<MQL	<MQL
Ifosfamide	<MQL	<MQL	<MQL	<MQL	<MQL	<MQL	<MQL	<MQL	<MQL	<MQL	<MQL	<MQL	<MQL	<MQL
Tamoxifen	<MQL	<MQL	<MQL	<MQL	<MQL	<MQL	<MQL	<MQL	<MQL	<MQL	<MQL	<MQL	<MQL	<MQL
Imatinib	<MQL	<MQL	<MQL	<MQL	<MQL	<MQL	<MQL	37	<MQL	39.6	<MQL	<MQL	<MQL	<MQL
Capecitabine	<MQL	<MQL	<MQL	<MQL	<MQL	<MQL	<MQL	<MQL	<MQL	<MQL	<MQL	<MQL	<MQL	<MQL
Bicalutamide	58.9	60.1	58.9	59.5	59.9	61.6	59.9	60.7	59	62	56.5	59	59.2	57
Ketamine	12	11	11.2	12	14.9	14.8	14.2	14.6	14.1	14.5	7.1	8.1	8.2	8.3
Norketamine	<MQL	<MQL	<MQL	<MQL	<MQL	<MQL	<MQL	<MQL	<MQL	<MQL	<MQL	<MQL	<MQL	<MQL
Venlafaxine	87.6	91.7	82.4	83	104.5	100.6	91.7	92.5	93.7	92.1	58.4	57.9	81.8	78.8
Desmethylvenlafaxine	238.9	232.2	214	226.8	266.2	250	246.4	250.4	245.4	248.6	182.7	186.6	218.2	202
Fluoxetine	1.4	1.2	0.8	1.1	1.2	1.2	1.2	1.2	1.2	1.1	1.3	1.4	1.3	1.4
Norfluoxetine	<MQL	<MQL	<MQL	<MQL	<MQL	<MQL	<MQL	<MQL	<MQL	<MQL	<MQL	<MQL	<MQL	<MQL
Sertraline	<MQL	<MQL	<MQL	<MQL	<MQL	<MQL	<MQL	<MQL	<MQL	<MQL	<MQL	<MQL	<MQL	<MQL
Mirtazapine	5.2	5	3.4	3.9	6.2	6.8	5.3	5.1	3.7	4.3	0.8	1.2	4.5	3.8
Citalopram	<MQL	<MQL	<MQL	<MQL	<MQL	<MQL	<MQL	<MQL	<MQL	<MQL	<MQL	<MQL	<MQL	<MQL
Desmethylcitalopram	11.9	14.7	9.2	9.1	19.9	19.4	15.9	12.5	11	10	<MQL	<MQL	6.5	4.1
Paroxetine	<MQL	<MQL	<MQL	<MQL	<MQL	<MQL	<MQL	<MQL	<MQL	<MQL	<MQL	<MQL	<MQL	<MQL
Duloxetine	<MQL	<MQL	<MQL	<MQL	<MQL	<MQL	<MQL	<MQL	<MQL	<MQL	<MQL	<MQL	<MQL	<MQL
Amitriptyline	16.2	12.7	11.4	12.9	12.2	<MQL	10.4	<MQL	5.5	11.1	<MQL	4.5	<MQL	<MQL

	Day 1		Day 2		Day 3		Day 4		Day 5		Day 6		Day 7	
**Compound**	**A**	**B**	**A**	**B**	**A**	**B**	**A**	**B**	**A**	**B**	**A**	**B**	**A**	**B**
Nortriptyline	<MQL	2.8	<MQL	<MQL	7.9	<MQL	<MQL	<MQL	<MQL	12.6	<MQL	<MQL	<MQL	<MQL
Norsertraline	N/A	N/A	N/A	N/A	N/A	N/A	N/A	N/A	N/A	N/A	N/A	N/A	N/A	N/A
Carbamazepine	162.3	164.5	152	153	184.7	185.5	174.6	177.4	168.7	168.1	142.5	144.7	136.7	131.2
Carbamazepine 10,11-epoxide	32.7	33.4	32.8	31.4	36.6	36	32.1	34.7	31.1	33.2	24.3	23.5	25.1	26
10,11-Dihydro -10-hydroxycarbamazepine	4.1	4.5	2.9	3	8.3	8.5	7.8	8.2	6.9	8.2	4.3	3.1	5.2	4.5
Diltiazem	0	5.6	4.6	3.6	8.8	7.7	4	4.9	4.3	5.6	<MQL	<MQL	2.7	1.1
Verapamil	<MQL	<MQL	<MQL	<MQL	<MQL	<MQL	<MQL	<MQL	<MQL	<MQL	<MQL	<MQL	<MQL	<MQL
Temazepam	<MQL	<MQL	<MQL	<MQL	<MQL	<MQL	<MQL	<MQL	<MQL	<MQL	<MQL	<MQL	<MQL	<MQL
Oxazepam	3.9	0.9	2.1	1.1	1.2	0.9	2.9	1.6	2.3	3.2	1	1.9	1.7	3.5
Diazepam	6.2	5.5	6	6	5.9	6	5.9	6	5.6	6	5.8	6	8.1	8.5
Quetiapine	<MQL	<MQL	<MQL	<MQL	<MQL	<MQL	<MQL	<MQL	<MQL	<MQL	<MQL	<MQL	<MQL	<MQL
Risperidone	5.1	1.3	0.3	0.2	<MQL	<MQL	<MQL	<MQL	<MQL	0.1	<MQL	<MQL	<MQL	<MQL
Donepezil	<MQL	<MQL	<MQL	<MQL	<MQL	<MQL	<MQL	<MQL	<MQL	<MQL	<MQL	<MQL	<MQL	<MQL
Memantine	<MQL	<MQL	<MQL	<MQL	<MQL	<MQL	<MQL	<MQL	<MQL	<MQL	<MQL	<MQL	<MQL	<MQL
Creatinine	<MQL	<MQL	<MQL	<MQL	<MQL	<MQL	<MQL	<MQL	<MQL	<MQL	<MQL	<MQL	<MQL	<MQL
Nicotine	31.1	30.5	24.2	20.5	31.2	32.6	23.4	21.2	23.9	22.8	64.5	65.9	54	56.8
Caffeine	429.2	426.3	409.7	408.2	460.4	467.2	392.8	383.7	375	373.2	309.2	297.2	620.7	617.6
Cotinine	39.4	38.9	36.9	34.8	38.7	42.1	34.2	36.7	35.4	32.9	28.6	31.5	48.9	50
1,7-dimethylxanthine	1051.7	1089.2	902.7	900.6	1038.6	1033.8	883.8	864	750.4	724.2	581.5	581.1	1235.1	1292.8
Morphine	<MQL	<MQL	<MQL	<MQL	<MQL	<MQL	<MQL	<MQL	<MQL	<MQL	<MQL	<MQL	<MQL	<MQL
Dihydromorphine	<MQL	<MQL	<MQL	<MQL	<MQL	<MQL	<MQL	<MQL	<MQL	<MQL	<MQL	<MQL	<MQL	<MQL
Normorphine	<MQL	<MQL	<MQL	<MQL	<MQL	<MQL	<MQL	<MQL	<MQL	<MQL	<MQL	<MQL	<MQL	<MQL
Methadone	4.3	3.7	2.8	2.4	4	4.1	3.5	3.5	3.1	3	1.6	1.8	2.5	2.3
EDDP	10.6	10.7	9.7	9.7	12.2	12.6	10.6	11.1	10.4	10	7.4	7.4	8.5	8.5
Codeine	96.7	83.6	80.8	72	100.4	100.2	91.5	87.1	76.3	77.9	51.2	53	119.9	108.2
Norcodeine	<MQL	<MQL	<MQL	<MQL	<MQL	<MQL	<MQL	<MQL	<MQL	<MQL	<MQL	<MQL	<MQL	<MQL
Dihydrocodeine	30.5	25	20.5	19.4	24.4	28.4	23.9	19.1	19.5	20.5	12.5	14	25.3	25.9

	Day 1		Day 2		Day 3		Day 4		Day 5		Day 6		Day 7	
**Compound**	**A**	**B**	**A**	**B**	**A**	**B**	**A**	**B**	**A**	**B**	**A**	**B**	**A**	**B**
Tramadol	328.9	323.8	291.6	301	370.9	352.4	344.2	323	352.1	348.9	277	280.5	312.3	296.7
N-desmethyltramadol	251.5	259	254.2	248	291.9	298.4	265.7	272.6	270.9	273.1	218.5	222.1	222.1	226.3
O-desmethyltramadol	310.4	305.2	278.6	273.3	338.8	347.5	327.9	319.2	313.9	316.4	242.8	235.4	277	271.8
Amphetamine	<MQL	<MQL	<MQL	<MQL	<MQL	<MQL	<MQL	<MQL	<MQL	<MQL	<MQL	<MQL	<MQL	<MQL
Methamphetamine	<MQL	<MQL	<MQL	<MQL	<MQL	<MQL	<MQL	<MQL	<MQL	<MQL	<MQL	<MQL	<MQL	<MQL
MDMA	3.6	3.6	2.7	2.5	3.7	3.6	4.6	5.1	8.2	8.5	2.3	2.2	5	4.5
MDA	11.7	9.3	11.1	11	9.1	9.1	12.4	9.8	9.1	9.8	8.1	9	9.7	10.3
Cocaine	5	5.1	3.4	3.6	7.1	7.8	5.7	6.2	4.2	4.2	2.3	2.4	5.7	5.8
Benzoylecgonine	54.9	55.9	42.3	41.3	43.3	45	47.4	48.5	44	45.9	25.1	26.3	57.5	59.6
Anhydroecgoninemethylester	<MQL	<MQL	<MQL	<MQL	<MQL	<MQL	<MQL	<MQL	<MQL	<MQL	<MQL	<MQL	<MQL	<MQL
Cocaethylene	<MQL	<MQL	<MQL	<MQL	<MQL	<MQL	<MQL	<MQL	<MQL	<MQL	<MQL	<MQL	<MQL	<MQL
Mephedrone	<MQL	<MQL	<MQL	<MQL	<MQL	<MQL	<MQL	<MQL	<MQL	<MQL	<MQL	<MQL	<MQL	<MQL
MDPV	<MQL	<MQL	<MQL	<MQL	<MQL	<MQL	<MQL	<MQL	<MQL	<MQL	<MQL	<MQL	<MQL	<MQL
Heroin	<MQL	<MQL	<MQL	<MQL	<MQL	<MQL	<MQL	<MQL	<MQL	<MQL	<MQL	<MQL	<MQL	<MQL
6-acetylmorphine	<MQL	<MQL	<MQL	<MQL	<MQL	<MQL	<MQL	<MQL	<MQL	<MQL	<MQL	<MQL	<MQL	<MQL
Thiamethoxam	<MQL	<MQL	<MQL	<MQL	<MQL	<MQL	<MQL	<MQL	<MQL	<MQL	<MQL	<MQL	<MQL	<MQL
Imidacloprid	55.8	55	48.9	55.2	49.7	57.4	60.7	80.2	74	87.5	45.1	33.7	20.3	18.7
Clothianidin	<MQL	<MQL	<MQL	<MQL	<MQL	<MQL	<MQL	<MQL	<MQL	<MQL	<MQL	<MQL	<MQL	<MQL
Metazachlor	<MQL	<MQL	<MQL	<MQL	<MQL	<MQL	<MQL	<MQL	<MQL	<MQL	<MQL	<MQL	3.9	5
Terbuthylazine	<MQL	<MQL	<MQL	<MQL	<MQL	<MQL	<MQL	<MQL	<MQL	<MQL	<MQL	<MQL	<MQL	<MQL
Methiocarb	<MQL	<MQL	<MQL	<MQL	<MQL	<MQL	<MQL	<MQL	<MQL	<MQL	<MQL	<MQL	<MQL	<MQL
Dichlofluanid	N/A	N/A	N/A	N/A	N/A	N/A	N/A	N/A	N/A	N/A	N/A	N/A	N/A	N/A
Flufenacet	22.4	22.5	22.6	22.3	22.2	22.6	22.4	22.7	22.9	23.4	25.6	26	29.6	28.4
Oxadiazon	<MQL	<MQL	<MQL	<MQL	<MQL	13.6	<MQL	<MQL	<MQL	21.5	<MQL	<MQL	15.5	<MQL
Chlorpyrifos	<MQL	<MQL	<MQL	<MQL	<MQL	<MQL	<MQL	<MQL	<MQL	<MQL	<MQL	<MQL	<MQL	<MQL
Triallate	<MQL	<MQL	<MQL	<MQL	<MQL	<MQL	<MQL	<MQL	<MQL	<MQL	<MQL	<MQL	<MQL	<MQL
Tylosin	<MQL	<MQL	<MQL	<MQL	<MQL	<MQL	<MQL	<MQL	<MQL	<MQL	<MQL	<MQL	<MQL	<MQL
Sulfapyridine	121.9	101	100.5	93.9	136.7	131.5	140.4	121	173.2	148.4	143.8	146.5	119.1	123.2

	Day 1		Day 2		Day 3		Day 4		Day 5		Day 6		Day 7	
**Compound**	**A**	**B**	**A**	**B**	**A**	**B**	**A**	**B**	**A**	**B**	**A**	**B**	**A**	**B**
Sarafloxacin	<MQL	<MQL	<MQL	<MQL	<MQL	<MQL	<MQL	<MQL	<MQL	<MQL	<MQL	<MQL	<MQL	<MQL
Ceftiofur	<MQL	<MQL	<MQL	<MQL	<MQL	<MQL	<MQL	<MQL	<MQL	<MQL	<MQL	31.2	<MQL	<MQL
Diazinon	6	6.1	6.1	6.2	6	6	5.9	5.9	6.2	7.4	6	6	6.5	5.9

Table S12 Effluent data (ng L^{-1})

Compound	Day 1 A	Day 1 B	Day 2 A	Day 2 B	Day 3 A	Day 3 B	Day 4 A	Day 4 B	Day 5 A	Day 5 B	Day 6 A	Day 6 B	Day 7 A	Day 7 B
Benzophenone-1	<MQL	<MQL	<MQL	<MQL	<MQL	<MQL	<MQL	<MQL	<MQL	<MQL	<MQL	<MQL	<MQL	<MQL
Benzophenone-2	36.2	27.9	39.2	40.8	104.2	141.9	57.3	48.9	<MQL	<MQL	<MQL	<MQL	<MQL	<MQL
Benzophenone-3	177.7	185.4	79.4	66.4	114.6	98.3	99.6	87.9	85.4	64.5	76.1	74.5	78.6	64.8
Benzophenone-4	2508.6	2695.1	2816.8	2775.3	6189.3	6422.8	12191.7	11422.4	3158	3357.4	1728.7	1747	1075.4	1058
Methylparaben	14.8	20.2	160.2	164.4	37.8	31.4	24.4	26.5	12.5	19.4	21.6	27.3	16.6	15
Ethylparaben	<MQL	<MQL	<MQL	<MQL	<MQL	<MQL	<MQL	<MQL	<MQL	<MQL	<MQL	<MQL	<MQL	<MQL
Propylparaben	23	20.7	54.9	56.8	24.8	32.3	30	25.5	18.1	19.2	36.4	38.2	35.8	33
Butylparaben	<MQL	<MQL	<MQL	<MQL	<MQL	<MQL	<MQL	<MQL	<MQL	<MQL	<MQL	<MQL	<MQL	<MQL
Bisphenol A	633.8	680.1	493.2	495.8	593.7	611.3	249.9	243	66.7	57.8	857.8	926.1	373.1	347.5
E1	4.1	7	1.4	5.5	8	7.4	5.2	6.8	6.4	5.3	10.2	7.4	4.9	8.8
E2	<MQL	<MQL	<MQL	<MQL	<MQL	<MQL	<MQL	<MQL	<MQL	<MQL	<MQL	<MQL	<MQL	<MQL
EE2	<MQL	<MQL	<MQL	<MQL	<MQL	<MQL	<MQL	<MQL	<MQL	<MQL	<MQL	<MQL	<MQL	<MQL
Sulfasalazine	281.6	307.9	219.1	233.4	241.8	237.1	216.2	269.1	241.5	239.2	167.7	222.8	251.3	251.7
Clarithromycin	843.2	870.1	921.1	915.4	854.6	919.7	824.6	837	673.2	708.9	674.3	703.7	563.4	558.4
Azithromycin	120.2	137.1	138.3	103.6	136.8	114.4	161.6	163.9	145.4	150.4	12.2	19.5	59.9	58.5
Trimethoprim	194.2	192.6	165.9	186.7	161.4	172.6	180.7	175.9	162.2	172.5	225	230.3	112.7	125.4
Sulfamethoxazole	55.5	61.8	61.4	60.9	52.3	50.9	51.2	56.2	47.5	54.1	60.3	62.7	43	42.5
Triclosan	279.2	233.6	132.1	154.7	344.9	282.1	267.2	234.5	241.2	157.5	201.5	197.7	175.3	190.9
Amoxicillin	<MQL	<MQL	<MQL	<MQL	<MQL	<MQL	<MQL	<MQL	<MQL	<MQL	<MQL	<MQL	<MQL	<MQL
Metronidazole	<MQL	<MQL	<MQL	27.5	40.8	42.5	45.8	47.8	36.9	<MQL	38.4	<MQL	33.7	27.5
Sulfadiazine	<MQL	<MQL	<MQL	<MQL	<MQL	<MQL	<MQL	<MQL	<MQL	<MQL	<MQL	<MQL	<MQL	<MQL
Cefalexin	<MQL	<MQL	<MQL	<MQL	<MQL	<MQL	<MQL	<MQL	<MQL	<MQL	<MQL	<MQL	<MQL	<MQL
Ofloxacin	241.3	124.1	<MQL	<MQL	<MQL	<MQL	<MQL	<MQL	<MQL	<MQL	79.3	<MQL	63.9	<MQL
Ciprofloxacin	<MQL	<MQL	<MQL	<MQL	<MQL	<MQL	<MQL	<MQL	<MQL	<MQL	<MQL	<MQL	<MQL	<MQL
Tetracycline	<MQL	<MQL	<MQL	<MQL	<MQL	<MQL	<MQL	<MQL	<MQL	<MQL	<MQL	<MQL	<MQL	<MQL
Danofloxacin	<MQL	<MQL	<MQL	<MQL	<MQL	<MQL	<MQL	<MQL	<MQL	<MQL	<MQL	<MQL	<MQL	<MQL
Oxytetracycline	<MQL	<MQL	<MQL	<MQL	<MQL	<MQL	<MQL	<MQL	<MQL	<MQL	<MQL	<MQL	<MQL	<MQL

Compound	Day 1 A	Day 1 B	Day 2 A	Day 2 B	Day 3 A	Day 3 B	Day 4 A	Day 4 B	Day 5 A	Day 5 B	Day 6 A	Day 6 B	Day 7 A	Day 7 B
Chloramphenicol	<MQL	<MQL	200.3	<MQL	<MQL	138.1	<MQL	<MQL	146.1	<MQL	165	<MQL	<MQL	139.9
Penicillin G	N/A	N/A	N/A	N/A	N/A	N/A	N/A	N/A	N/A	N/A	N/A	N/A	N/A	N/A
Penicillin V	<MQL	<MQL	<MQL	<MQL	<MQL	<MQL	<MQL	<MQL	<MQL	<MQL	<MQL	<MQL	<MQL	<MQL
Erythromycin	7087.1	6895.2	7239.8	7380.2	6739.1	6930.4	6278	6391.6	5945.6	5908.5	6752.1	6746.7	5602.2	5156.1
Prulifloxacin	<MQL	<MQL	<MQL	<MQL	<MQL	<MQL	<MQL	<MQL	<MQL	<MQL	<MQL	<MQL	<MQL	<MQL
Norfloxacin	<MQL	<MQL	<MQL	<MQL	<MQL	<MQL	<MQL	<MQL	<MQL	<MQL	<MQL	<MQL	<MQL	<MQL
Griseofulvin	<MQL	<MQL	<MQL	<MQL	<MQL	<MQL	<MQL	<MQL	<MQL	<MQL	<MQL	<MQL	<MQL	<MQL
Ketoconazole	82.2	65.2	65.3	67.6	57.5	64	66.3	<MQL	<MQL	<MQL	56.6	60.2	64.2	56
Valsartan	242.9	275.1	208.6	218.2	216.1	229.6	219.6	236.4	237.5	224.4	280.4	285.1	227.7	214.5
Irbesartan	311.5	315.6	307.1	316	280.2	323.7	293.1	291.8	284	298.7	286.8	285.1	223	213.8
Lisinopril	92.3	90.8	62.3	65.6	91.9	110.9	98.2	108.6	91.6	83.7	121.5	119	97.7	80.5
Ketoprofen	<MQL	<MQL	<MQL	<MQL	<MQL	<MQL	<MQL	<MQL	<MQL	<MQL	<MQL	<MQL	<MQL	<MQL
Ibuprofen	1396	1448.6	786.5	844.8	897.8	925.6	792.3	834.1	648.2	644.8	1769.9	1655.6	828	937.1
Naproxen	1711.4	1853.1	1193.4	1201.8	1292.6	1381.1	1429.2	1440.8	1231.9	1192.9	1661	1787.3	1480.5	1477.3
Diclofenac	335.3	387.8	367.4	395.3	356.8	337.5	410	397.4	344.2	348.3	341	366.8	227	223.8
Acetaminophen	2482.2	2457.3	1634	1634	1771.2	1721.3	1933.4	1521.8	1022.8	935.5	2232.7	2145.4	2157.9	2120.5
Bezafibrate	669	704.1	587.6	619.5	603.8	565.3	625.4	589.6	537.4	547.1	660.2	690.2	442.6	442.2
Atorvastatin	224.3	236.2	171	167.2	169.6	167.3	169.6	195.3	129.3	143.1	159.3	178	155.7	154.5
Gemfibrozil	<MQL	<MQL	<MQL	<MQL	<MQL	<MQL	<MQL	<MQL	<MQL	<MQL	<MQL	<MQL	<MQL	<MQL
Candesartan Cilexetil	N/A	N/A	N/A	N/A	N/A	N/A	N/A	N/A	N/A	N/A	N/A	N/A	N/A	N/A
Fexofenadine	792.8	874.9	797.7	835.5	702.7	645.9	794.9	740.1	679.1	723.1	803.8	824.3	514.4	526.8
Cetirizine	799.1	839.7	911.2	916.6	1018.7	1055.7	1115.5	1088	1181.3	1176.1	1300.9	1336.5	957.7	1017.8
Sildenafil	11.2	9.8	42.5	40.1	46.6	42.8	27.4	27.2	23.1	22.7	20.3	21.1	12.9	9.9
Metformin	16251.2	14983.1	12922.6	13137.1	14153.3	14367.8	14889.9	14190.6	12829.4	12810.7	15067.1	13845.7	13416.8	13845.7
Gliclazide	88.8	79.6	77.8	81.5	94.3	91.9	93.2	98.1	83.5	83.4	80.4	84.6	72.2	70.7
Sitagliptin	446.5	432	471.8	399.9	422.7	403.5	499.3	475	443.1	519.1	489.1	571.6	324.6	281.4
Pholcodine	<MQL	<MQL	<MQL	<MQL	<MQL	<MQL	<MQL	<MQL	<MQL	<MQL	<MQL	<MQL	<MQL	<MQL
Atenolol	346.9	350.7	314	298.9	346.4	306.4	364.6	367.1	289	309.3	371.9	370.5	303.7	265.6
Metoprolol	15	10.2	11	11.4	12.4	15.6	21	13	15.7	15.5	14.3	15.9	8.7	7.1

	Day 1		Day 2		Day 3		Day 4		Day 5		Day 6		Day 7	
**Compound**	**A**	**B**	**A**	**B**	**A**	**B**	**A**	**B**	**A**	**B**	**A**	**B**	**A**	**B**
Propranolol	103.5	103.5	103.4	105.1	108.2	110.7	121.9	109.8	93.2	98.5	111.7	113.4	111.5	98
Bisoprolol	14.8	14.6	<MQL	<MQL	<MQL	<MQL	<MQL	<MQL	<MQL	<MQL	<MQL	<MQL	<MQL	<MQL
Ranitidine	1277.1	1219.2	1336	1241.8	1227.8	1054.3	1206.6	1161.2	1095.3	980.3	1283.9	1176.8	1053.7	968
Cimetidine	<MQL	<MQL	<MQL	<MQL	<MQL	<MQL	<MQL	<MQL	<MQL	<MQL	<MQL	<MQL	<MQL	<MQL
Iopromide	<MQL	<MQL	<MQL	<MQL	<MQL	<MQL	<MQL	<MQL	<MQL	<MQL	<MQL	<MQL	<MQL	<MQL
Buprenorphine	<MQL	<MQL	<MQL	<MQL	<MQL	<MQL	<MQL	<MQL	<MQL	<MQL	<MQL	<MQL	5.7	<MQL
Ephedrine/pseudoephedrine	117.5	109.3	151.5	142.6	135.1	132.7	105.7	108.7	105.4	93.8	122.7	115.5	95.3	81.2
Norephedrine	<MQL	<MQL	<MQL	<MQL	<MQL	<MQL	<MQL	<MQL	<MQL	<MQL	<MQL	<MQL	<MQL	<MQL
Azathioprine	<MQL	<MQL	<MQL	<MQL	<MQL	<MQL	<MQL	<MQL	<MQL	<MQL	<MQL	<MQL	<MQL	<MQL
Methotrexate	<MQL	<MQL	<MQL	<MQL	<MQL	<MQL	<MQL	<MQL	<MQL	<MQL	<MQL	<MQL	<MQL	<MQL
Ifosfamide	<MQL	<MQL	<MQL	<MQL	<MQL	<MQL	<MQL	<MQL	<MQL	<MQL	<MQL	<MQL	<MQL	<MQL
Tamoxifen	<MQL	<MQL	<MQL	<MQL	<MQL	<MQL	<MQL	<MQL	<MQL	<MQL	<MQL	<MQL	<MQL	<MQL
Imatinib	<MQL	173	188.9	<MQL	150.3	<MQL	143.8	115.1	<MQL	<MQL	<MQL	<MQL	<MQL	75.1
Capecitabine	<MQL	<MQL	<MQL	<MQL	<MQL	<MQL	<MQL	<MQL	<MQL	<MQL	<MQL	<MQL	<MQL	<MQL
Bicalutamide	126	124.4	117.2	116.5	111.3	120.9	122	117.6	114.3	116.9	114.6	113.8	101.4	101.7
Ketamine	56.7	57.4	81.7	82	74.2	81.6	74.6	74.8	78.3	78.6	94.6	94.1	62.8	64
Norketamine	5	4.1	5.5	3.9	2.9	3.3	3	5.6	7.4	6.8	11.5	12.5	2.9	5.3
Venlafaxine	611.1	622	602	624.3	563.1	574.8	597.4	597.7	567.5	584.1	271.7	276.4	325.5	315.4
Desmethylvenlafaxine	1040.2	1075.7	1063.6	1041.3	977.9	1065.1	1003.3	1072.3	981.5	1017.5	1030.6	1027.1	700.9	637.3
Fluoxetine	37.6	48	45.1	51.9	43.7	44.5	50	53.5	39	47.3	42.8	39.8	56.7	46.1
Norfluoxetine	<MQL	<MQL	<MQL	<MQL	<MQL	<MQL	<MQL	<MQL	<MQL	<MQL	<MQL	<MQL	<MQL	<MQL
Sertraline	12.7	16.3	11	11.1	13.4	15	17.7	14.5	11.2	12.9	11.3	12.3	11	12.7
Mirtazapine	41.7	43.9	40	43.4	39.5	42.5	45.6	41.2	39.6	42.7	41.1	45.5	36	35.8
Citalopram	337.1	321.3	301.2	342.3	338.9	340	349.9	324.8	307.1	331.8	314.9	344.7	269.7	282.3
Desmethylcitalopram	121.6	116.2	104.7	112.5	125.7	124.4	127.6	122.2	116.4	114.3	136.3	142.8	104	106.7
Paroxetine	<MQL	<MQL	<MQL	<MQL	<MQL	<MQL	<MQL	<MQL	<MQL	<MQL	<MQL	<MQL	<MQL	<MQL
Duloxetine	<MQL	<MQL	<MQL	<MQL	<MQL	<MQL	<MQL	<MQL	<MQL	<MQL	<MQL	<MQL	<MQL	<MQL
Amitriptyline	61.3	65.3	63.4	55	52	45.8	52.2	46.5	50.5	47.5	57.8	62.3	46.9	39.9

	Day 1		Day 2		Day 3		Day 4		Day 5		Day 6		Day 7	
**Compound**	**A**	**B**	**A**	**B**	**A**	**B**	**A**	**B**	**A**	**B**	**A**	**B**	**A**	**B**
Nortriptyline	35.6	22.2	35.4	32.5	37.9	30.2	35	25.5	26.6	<MQL	27.6	38.9	33.1	21.1
Norsertraline	N/A	N/A	N/A	N/A	N/A	N/A	N/A	N/A	N/A	N/A	N/A	N/A	N/A	N/A
Carbamazepine	649	663.5	653.2	664.8	619.4	633.5	672.6	653.7	658	639.4	652.3	653.9	473	489
Carbamazepine 10,11-epoxide	157.4	161.1	164.3	171.7	163	156.1	152.4	154.5	164.9	145.9	99.9	78.2	81.7	84.2
10,11-Dihydro -10-hydroxycarbamazepine	46.3	45	24.3	22.6	27.6	29	47	48.2	48.4	52.3	66.3	65.2	39.5	43.5
Diltiazem	52.1	54.1	46.6	51.2	53.1	56	60.6	62.5	57.1	58.5	81.2	84.8	57.5	57.9
Verapamil	<MQL	<MQL	<MQL	<MQL	<MQL	<MQL	<MQL	<MQL	<MQL	<MQL	<MQL	<MQL	<MQL	<MQL
Temazepam	2.7	3.7	6.4	9.5	16.2	19.3	32.4	27.6	37.4	34.9	42.1	43.6	12.2	13.1
Oxazepam	16.7	22.3	16.6	17.9	17.7	16.6	17.3	18.7	17	22	19.7	24.1	7.4	8.7
Diazepam	14.2	14.2	11.9	<MQL	10.7	10.9	10.4	10.8	11.4	10.9	12.5	11.4	10.9	10.3
Quetiapine	<MQL	<MQL	<MQL	<MQL	<MQL	<MQL	<MQL	<MQL	<MQL	<MQL	<MQL	<MQL	<MQL	<MQL
Risperidone	4.2	2.4	1.8	<MQL	<MQL	<MQL	<MQL	<MQL	<MQL	<MQL	<MQL	<MQL	<MQL	<MQL
Donepezil	<MQL	<MQL	<MQL	<MQL	<MQL	<MQL	<MQL	<MQL	<MQL	<MQL	<MQL	<MQL	<MQL	<MQL
Memantine	<MQL	<MQL	<MQL	<MQL	<MQL	<MQL	<MQL	<MQL	<MQL	<MQL	<MQL	<MQL	<MQL	<MQL
Creatinine	<MQL	<MQL	<MQL	<MQL	<MQL	<MQL	<MQL	<MQL	<MQL	<MQL	<MQL	<MQL	<MQL	<MQL
Nicotine	214.1	220.5	137.2	115.3	187.1	181.9	187	198.4	163.6	159.7	242.3	249.3	147.5	170.3
Caffeine	3086.9	3187	2464.7	2453.1	2803.2	2814.7	3062.9	3070.2	2453.1	2540.8	3776.5	3683.5	2852.7	2895.6
Cotinine	261.8	275.2	182.9	197.4	210	204.3	220.4	221.2	189	191.1	241.1	238.9	189.5	180.3
1,7-dimethylxanthine	8959.6	9717.4	7212.3	6632.8	7604.5	7577.8	7827.4	9378.6	7970.1	7675.9	11010.1	9744.2	7488.6	8130.5
Morphine	171.3	178.5	139	139	107.2	138.8	145.9	151.8	139.7	128.4	152.4	155.5	132.2	104.6
Dihydromorphine	<MQL	<MQL	<MQL	<MQL	<MQL	<MQL	<MQL	<MQL	<MQL	<MQL	<MQL	<MQL	<MQL	<MQL
Normorphine	<MQL	<MQL	<MQL	<MQL	<MQL	<MQL	<MQL	<MQL	<MQL	<MQL	<MQL	<MQL	<MQL	<MQL
Methadone	21.8	20.4	20.5	23.1	22.5	23.9	24.8	23.8	21.2	23.4	22.5	23.1	19.8	18.8
EDDP	50.2	51.1	50	51.7	51.4	52.7	53.6	53.1	49.1	50.9	50.2	52.8	43.1	43.6
Codeine	597.2	602.1	606.2	584.7	563.6	587.6	623.7	645.3	539.2	509.1	702	594.9	485.4	449.6
Norcodeine	<MQL	<MQL	<MQL	<MQL	<MQL	<MQL	<MQL	<MQL	<MQL	<MQL	<MQL	<MQL	<MQL	<MQL
Dihydrocodeine	138.2	154.6	167.1	142.4	157.3	173.3	161	170.8	155.7	141.2	182.3	181.3	104.9	126.4

Compound	Day 1 A	Day 1 B	Day 2 A	Day 2 B	Day 3 A	Day 3 B	Day 4 A	Day 4 B	Day 5 A	Day 5 B	Day 6 A	Day 6 B	Day 7 A	Day 7 B
Tramadol	1370.4	1413.6	1354.8	1377.3	1322.1	1400.5	1400.8	1358.5	1289.4	1316.3	1213.2	1239.8	916.5	858.9
N-desmethyltramadol	1026	1003.8	1054.4	1112.1	1072.1	1045.5	1059	1027	980.8	1010.8	917.8	927.2	719.1	702.2
O-desmethyltramadol	1474	1478.7	1388	1403.6	1370.8	1435.4	1429.8	1473.2	1309	1341.6	1183.8	1198.6	859.6	860.1
Amphetamine	<MQL	<MQL	<MQL	<MQL	<MQL	<MQL	<MQL	<MQL	<MQL	<MQL	<MQL	<MQL	<MQL	<MQL
Methamphetamine	10.4	10.4	8.5	8.6	8.3	8.8	8.4	7.8	8.8	8.9	9.7	10	9.5	9.3
MDMA	34.6	35.9	25.6	27.3	26.9	27.1	55.4	55	87.1	91.8	124.4	123	56	60.9
MDA	27.8	27.1	18.7	22.9	22.9	21.3	26	27.5	32.4	39.6	44.5	43.2	36.3	31.3
Cocaine	40.8	41.5	35.7	36.6	49.4	50.7	72.5	67.7	54.3	55.4	43.6	45.5	33.3	32.3
Benzoylecgonine	223.7	238.7	206.4	210.5	241.2	242.8	413.8	426.8	406.9	404.8	361	369.9	161.9	168.1
Anhydroecgoninemethylester	<MQL	<MQL	<MQL	<MQL	<MQL	<MQL	<MQL	<MQL	<MQL	<MQL	<MQL	<MQL	<MQL	<MQL
Cocaethylene	2.7	2.6	2.7	2.8	3.2	2.9	4.8	5.5	4.9	5	4.4	4	2.5	2.2
Mephedrone	<MQL	<MQL	<MQL	<MQL	<MQL	<MQL	<MQL	<MQL	<MQL	<MQL	<MQL	<MQL	<MQL	<MQL
MDPV	<MQL	<MQL	<MQL	<MQL	<MQL	<MQL	<MQL	<MQL	<MQL	<MQL	<MQL	<MQL	<MQL	<MQL
Heroin	<MQL	<MQL	<MQL	<MQL	<MQL	<MQL	<MQL	<MQL	<MQL	<MQL	<MQL	<MQL	<MQL	<MQL
6-acetylmorphine	<MQL	<MQL	<MQL	<MQL	<MQL	<MQL	<MQL	<MQL	<MQL	<MQL	<MQL	<MQL	<MQL	<MQL
Thiamethoxam	<MQL	<MQL	<MQL	<MQL	<MQL	<MQL	<MQL	<MQL	<MQL	<MQL	<MQL	<MQL	<MQL	<MQL
Imidacloprid	437.1	349.5	298.7	289.1	267.5	243	403.8	362	495.4	460.5	501.3	548.3	94	102.9
Clothianidin	<MQL	<MQL	<MQL	<MQL	<MQL	<MQL	<MQL	<MQL	<MQL	<MQL	<MQL	<MQL	<MQL	<MQL
Metazachlor	<MQL	<MQL	<MQL	<MQL	<MQL	<MQL	<MQL	<MQL	<MQL	<MQL	<MQL	<MQL	<MQL	<MQL
Terbuthylazine	<MQL	<MQL	<MQL	<MQL	<MQL	<MQL	<MQL	<MQL	<MQL	<MQL	<MQL	<MQL	<MQL	<MQL
Methiocarb	<MQL	<MQL	3	<MQL	<MQL	<MQL	<MQL	<MQL	<MQL	<MQL	<MQL	<MQL	<MQL	<MQL
Dichlofluanid	N/A	N/A	N/A	N/A	N/A	N/A	N/A	N/A	N/A	N/A	N/A	N/A	N/A	N/A
Flufenacet	59.4	<MQL	60.7	<MQL	59.3	59.2	59.9	59.2	59.3	59.4	65.3	65.3	69.1	68.5
Oxadiazon	<MQL	34.5	28.2	<MQL	<MQL	<MQL	<MQL	<MQL	<MQL	<MQL	<MQL	<MQL	26.6	<MQL
Chlorpyrifos	<MQL	<MQL	<MQL	<MQL	<MQL	<MQL	<MQL	<MQL	<MQL	<MQL	<MQL	<MQL	<MQL	<MQL
Triallate	<MQL	<MQL	<MQL	<MQL	<MQL	<MQL	<MQL	<MQL	<MQL	<MQL	<MQL	<MQL	<MQL	<MQL
Tylosin	<MQL	<MQL	<MQL	<MQL	<MQL	<MQL	<MQL	<MQL	<MQL	<MQL	<MQL	<MQL	<MQL	<MQL
Sulfapyridine	580.5	540.9	797.7	786.2	506.4	498.5	587.4	497.9	662.1	580.9	722.4	565.2	344.4	393.6

	Day 1		Day 2		Day 3		Day 4		Day 5		Day 6		Day 7	
**Compound**	**A**	**B**	**A**	**B**	**A**	**B**	**A**	**B**	**A**	**B**	**A**	**B**	**A**	**B**
Sarafloxacin	<MQL	<MQL	<MQL	<MQL	<MQL	<MQL	<MQL	<MQL	<MQL	<MQL	<MQL	<MQL	<MQL	<MQL
Ceftiofur	<MQL	<MQL	<MQL	<MQL	<MQL	<MQL	<MQL	<MQL	<MQL	<MQL	321.7	264.9	123.3	154.4
Diazinon	11.8	13	13.6	11.6	11.9	11.5	12.1	11.6	11.7	11.7	11.5	11.8	12.6	11.8

Table S13 Influent data (ng L^{-1})

Compound	Day 1 A	Day 1 B	Day 2 A	Day 2 B	Day 3 A	Day 3 B	Day 4 A	Day 4 B	Day 5 A	Day 5 B	Day 6 A	Day 6 B	Day 7 A	Day 7 B
Benzophenone-1	589.6	557.7	856.8	805.5	2712.7	4675.3	463.7	454.4	230.3	246.9	542.7	518.4	320.9	313.3
Benzophenone-2	906.5	812	2902.1	2825.6	6575.2	6622.6	903.3	818.9	252.2	242.8	155.7	152.8	327.9	316.5
Benzophenone-3	3865.3	3678.5	1422.5	1410.7	3100	2927.2	2007.7	1906.3	1268.2	1216.3	1622	1579.7	1367.7	1349.1
Benzophenone-4	8481.2	8567.9	15251	14741.9	45395.6	40824.6	25292	29646.3	7593	7452.2	3444.5	4625.1	5090.9	5036.7
Methylparaben	6747.9	6546.1	109530.2	113301.5	11755.2	11919.2	19411.2	19587.8	5146.1	5827.2	4830.7	4944.2	3846.9	4225.3
Ethylparaben	2267	2564.3	1321.7	1390.4	1382	1278.9	8239.9	7629	1127.9	869.9	834.5	662.6	365	391.6
Propylparaben	4891	4904.7	12625.9	11980.2	3599.6	3324.8	7693.7	7872.3	2898.9	3352.3	3462.2	3379.7	3269.8	3132.4
Butylparaben	162.3	135	69.6	72.7	110.2	117.9	1440.7	1335.7	146.3	149.8	194.1	166.4	311.6	310.9
Bisphenol A	42439.1	40048.6	28356.2	25688.6	29291.6	30071.1	3741.6	3395.1	2650.3	2979.4	34522.9	37814.1	3066	1472.4
E1	47.2	55.1	52.1	70.1	38.5	49.5	55.5	34	64.3	55.2	45.6	34.1	29	27.4
E2	<MQL	<MQL	<MQL	<MQL	<MQL	<MQL	<MQL	<MQL	<MQL	<MQL	<MQL	<MQL	<MQL	<MQL
EE2	<MQL	<MQL	<MQL	<MQL	<MQL	<MQL	<MQL	<MQL	<MQL	<MQL	<MQL	<MQL	<MQL	<MQL
Sulfasalazine	1183.7	1093.5	552.8	593	765.3	655.2	601.7	621.4	618.7	416.6	499.5	430.1	296.8	252.4
Clarithromycin	1438	1342	1370.7	1336.8	1904.9	1860.7	1522.5	1461.1	1064.6	1104.9	956.1	900.5	1119.7	1113.5
Azithromycin	1040	985.1	880.7	872.7	506.1	516.5	450.7	363.8	606.1	685.7	1081	1231.7	913	969.9
Trimethoprim	616.9	640.3	615.8	631.6	687.5	745.7	671.2	651.6	866.5	922.1	471.8	459.7	392.4	393.5
Sulfamethoxazole	237	243.7	139.1	139.3	139.3	134.4	122.5	141.9	134.2	128.8	60.8	54.6	43.5	54.7
Triclosan	2161.2	2068.2	2155.5	1862.8	2720.2	2935.1	2527.6	2335.5	2127.8	1935.1	4488.9	4040.8	1811.5	1557.3
Amoxicillin	N/A	N/A	N/A	N/A	N/A	N/A	N/A	N/A	N/A	N/A	N/A	N/A	N/A	N/A
Metronidazole	103.7	95.3	<MQL	65.3	123.5	126.9	<MQL	<MQL	75.6	63.8	55.9	<MQL	67.6	80.4
Sulfadiazine	<MQL	<MQL	<MQL	<MQL	<MQL	<MQL	<MQL	<MQL	<MQL	<MQL	<MQL	<MQL	<MQL	<MQL
Cefalexin	<MQL	<MQL	<MQL	<MQL	<MQL	<MQL	<MQL	<MQL	<MQL	<MQL	<MQL	<MQL	<MQL	<MQL
Ofloxacin	<MQL	<MQL	<MQL	<MQL	<MQL	<MQL	<MQL	<MQL	<MQL	<MQL	<MQL	<MQL	<MQL	<MQL
Ciprofloxacin	<MQL	<MQL	<MQL	<MQL	<MQL	<MQL	<MQL	<MQL	<MQL	<MQL	<MQL	<MQL	<MQL	<MQL
Tetracycline	<MQL	<MQL	<MQL	322	<MQL	<MQL	181.1	<MQL	<MQL	<MQL	<MQL	<MQL	<MQL	<MQL
Danofloxacin	1468.1	440.3	<MQL	138.7	24.1	<MQL	<MQL	<MQL	<MQL	<MQL	<MQL	<MQL	<MQL	<MQL
Oxytetracycline	<MQL	<MQL	<MQL	<MQL	<MQL	<MQL	<MQL	<MQL	<MQL	<MQL	<MQL	<MQL	<MQL	<MQL

	Day 1		Day 2		Day 3		Day 4		Day 5		Day 6		Day 7	
**Compound**	**A**	**B**	**A**	**B**	**A**	**B**	**A**	**B**	**A**	**B**	**A**	**B**	**A**	**B**
Chloramphenicol	<MQL	<MQL	<MQL	<MQL	<MQL	<MQL	<MQL	<MQL	<MQL	<MQL	<MQL	<MQL	<MQL	<MQL
Penicillin G	N/A	N/A	N/A	N/A	N/A	N/A	N/A	N/A	N/A	N/A	N/A	N/A	N/A	N/A
Penicillin V	<MQL	<MQL	<MQL	<MQL	<MQL	<MQL	<MQL	<MQL	<MQL	<MQL	<MQL	<MQL	<MQL	<MQL
Erythromycin	16689.5	17722.8	15790.5	15730.6	15005.6	15297.1	13601.3	13215	11408.6	11267.2	12575.1	12710	9479.8	9788.1
Prulifloxacin	<MQL	<MQL	<MQL	<MQL	<MQL	<MQL	<MQL	<MQL	<MQL	<MQL	<MQL	<MQL	<MQL	<MQL
Norfloxacin	<MQL	<MQL	<MQL	<MQL	<MQL	<MQL	<MQL	<MQL	<MQL	<MQL	<MQL	<MQL	<MQL	<MQL
Griseofulvin	<MQL	<MQL	<MQL	<MQL	<MQL	<MQL	<MQL	<MQL	<MQL	<MQL	<MQL	<MQL	<MQL	<MQL
Ketoconazole	140.9	189.5	123	132.8	154.9	163.6	109	112.8	185.7	203.4	87.1	80.1	73.5	78
Valsartan	757.8	709.6	904.6	926.5	935.3	860.6	1422.5	1425.5	1023.4	745.1	929.6	739.6	301.3	287.6
Irbesartan	463.8	421.6	400.9	373.4	425.3	414.1	479.9	452	366.6	378.5	365.2	336.8	235.4	216.3
Lisinopril	740	796.1	926.8	779.5	855.6	928	1497.2	1388.7	1081.7	1067.1	629.1	648.8	607.6	520.5
Ketoprofen	<MQL	<MQL	<MQL	<MQL	<MQL	<MQL	<MQL	<MQL	<MQL	<MQL	<MQL	<MQL	<MQL	<MQL
Ibuprofen	26399.5	24884.9	23870.1	23627.8	22613	23234	16736.4	17266.5	16448.6	17357.3	17599.7	17175.6	9011.9	7542.7
Naproxen	19304	16694.3	16102.4	16331	17662.8	19573	17582.1	16855.7	16344.5	14528.4	9564.6	10533.1	11555.5	10708
Diclofenac	811.1	671.8	764.6	788.2	1081.2	1041.5	1691.3	1602.2	1092.4	898	991.1	707.3	338.4	347.6
Acetaminophen	497085.7	488975.2	440391.2	429786.3	470886.8	473172.4	453346.9	455736.9	423621.8	420226.2	247334.8	240282.2	236834.3	232145.7
Bezafibrate	2321.8	2286.9	2304.9	2369.4	2137.6	2180	2603.8	2397.9	2302.9	2296.1	1549.7	1528.1	1132.7	1123.8
Atorvastatin	1573.6	1852.7	1431	1556.5	1666.3	1429.8	1927.3	1854.6	1265.8	958.4	1371.6	1041.2	398.2	407.4
Gemfibrozil	<MQL	<MQL	<MQL	<MQL	<MQL	<MQL	<MQL	<MQL	<MQL	<MQL	<MQL	<MQL	<MQL	<MQL
Candesartan Cilexetil	N/A	N/A	N/A	N/A	N/A	N/A	N/A	N/A	N/A	N/A	N/A	N/A	N/A	N/A
Fexofenadine	802.8	1343.5	891.9	1370.5	167.4	160.4	223.1	219.9	1510.6	1591	2498.4	2025.2	685.1	680.3
Cetirizine	1046.9	1040.6	1099	1144.5	1013.5	1118.4	1204	1159.1	1310.1	1211.9	1084	1005.4	906.1	893.9
Sildenafil	3.7	8	125.2	134.5	18.8	21.4	17.4	18.3	15.1	20.8	6.8	6.5	4.2	5.6
Metformin	145236.8	138418.9	129059.7	122752.1	133968.9	135642.8	137459.5	139755.9	127100	120363.8	87468.7	83620.9	76884.7	73251.2
Gliclazide	118.4	124.4	120.4	125.4	203.5	211.7	195.6	191.2	106.5	113.7	102.9	105.8	94.5	96.3
Sitagliptin	628.8	594.2	547.4	544.3	385.8	418.8	454	442.9	473.5	487.8	607	631.1	549.2	581.1
Pholcodine	<MQL	<MQL	<MQL	<MQL	<MQL	<MQL	<MQL	<MQL	<MQL	<MQL	<MQL	<MQL	<MQL	<MQL
Atenolol	3302.6	3260.1	2658.5	2740.7	2768.2	2948.1	3040.6	2944.3	2757.2	2822.4	2196.1	1768.6	1454.6	1482.1

Compound	Day 1		Day 2		Day 3		Day 4		Day 5		Day 6		Day 7	
	**A**	**B**	**A**	**B**	**A**	**B**	**A**	**B**	**A**	**B**	**A**	**B**	**A**	**B**
Metoprolol	29.9	27.3	28.5	32	39.4	40.3	42.9	45.1	43	37	30.5	23.5	16.6	13.2
Propranolol	203.9	205	185.5	209.5	195.7	210.5	187.3	202.3	205.1	202	166.2	143.1	122.4	113.1
Bisoprolol	80	93.3	38.3	37.2	3	<MQL	14.3	6.4	43.6	38.4	<MQL	<MQL	2.7	6.5
Ranitidine	1625.7	1615.8	1940.2	1408.6	1436.7	1626.1	1560.9	1358.7	2033.2	2108.2	1554.5	1163.1	1085.6	1113.1
Cimetidine	277.6	288.4	177.1	231.2	159.9	132.1	202.9	188.1	193.1	177.9	109.4	101.3	77.7	67.8
Iopromide	<MQL	<MQL	<MQL	<MQL	<MQL	<MQL	<MQL	<MQL	<MQL	<MQL	<MQL	<MQL	<MQL	<MQL
Buprenorphine	<MQL	<MQL	41.1	<MQL	102.9	<MQL	<MQL	<MQL	108.7	<MQL	38.1	32.3	16.2	<MQL
Ephedrine/pseudoephedrine	944	920.1	957.4	974.4	879.7	768.3	605.9	608.7	645.5	598.6	347.9	338.5	292.3	296.2
Norephedrine	<MQL	<MQL	<MQL	<MQL	<MQL	<MQL	<MQL	<MQL	<MQL	<MQL	<MQL	<MQL	<MQL	<MQL
Azathioprine	<MQL	<MQL	<MQL	<MQL	<MQL	<MQL	<MQL	<MQL	<MQL	<MQL	<MQL	<MQL	<MQL	<MQL
Methotrexate	<MQL	<MQL	<MQL	<MQL	<MQL	<MQL	<MQL	<MQL	<MQL	<MQL	<MQL	<MQL	<MQL	<MQL
Ifosfamide	<MQL	<MQL	<MQL	<MQL	<MQL	<MQL	<MQL	<MQL	<MQL	<MQL	<MQL	<MQL	<MQL	<MQL
Tamoxifen	<MQL	<MQL	<MQL	<MQL	<MQL	<MQL	<MQL	<MQL	<MQL	<MQL	<MQL	<MQL	<MQL	<MQL
Imatinib	109	89.3	<MQL	62.4	94.4	113.4	89.7	<MQL	63	115.3	<MQL	<MQL	<MQL	<MQL
Capecitabine	7.2	13.3	8.6	8.1	6	3.5	3.9	3.7	10.9	11.8	5.4	5.5	<MQL	<MQL
Bicalutamide	169.6	178.7	161.4	158.4	144.7	152.4	154.6	160	154.6	147.2	142.8	155.2	139.7	141.3
Ketamine	203.6	207.5	249.2	260.6	147.4	157.4	163.4	164.2	246.2	218.6	89.9	85	66.3	67
Norketamine	3	5.3	13.5	13.4	10.9	9	15.3	16	23.5	22.7	18.9	15.9	1.6	1.3
Venlafaxine	532.6	521.4	413.3	397.7	641.1	706.9	588.9	566.8	467.3	510.5	281.3	251	318.7	275.8
Desmethylvenlafaxine	870.3	867.3	821	853.5	700.9	729.6	712.9	744	854.3	770.6	753.1	700.5	439.5	457
Fluoxetine	54.2	53.2	46.8	45.1	57.6	54.3	57.9	46	66	69.4	52.3	52.3	44.5	44.6
Norfluoxetine	31.9	28.1	21.9	24.8	41.3	25.5	30.4	27.8	26.1	34.4	53.8	38.6	30.5	30.4
Sertraline	52.2	48.8	42.2	46.5	54.1	42.8	54.2	56	48.8	54.5	36.6	28.9	41.6	41
Mirtazapine	85.8	78.4	77.5	84.6	87.5	92.9	90.5	87.1	83.5	85.1	75.2	68.9	48.1	50.3
Citalopram	685.9	691.5	566.1	596.9	617.6	654.6	636.7	573.2	594.8	602.5	443.9	443.8	349.8	347.4
Desmethylcitalopram	266.7	268.3	178.6	194	279	280.7	307.5	255.9	216.6	218.1	169.9	156.1	115.3	119.6
Paroxetine	<MQL	<MQL	<MQL	<MQL	<MQL	<MQL	<MQL	<MQL	<MQL	<MQL	<MQL	<MQL	<MQL	<MQL
Duloxetine	<MQL	<MQL	<MQL	<MQL	<MQL	<MQL	<MQL	<MQL	<MQL	<MQL	<MQL	<MQL	<MQL	<MQL

Compound	Day 1		Day 2		Day 3		Day 4		Day 5		Day 6		Day 7	
	A	B	A	B	A	B	A	B	A	B	A	B	A	B
Amitriptyline	189	168.7	139.8	161.3	196.9	204.1	143.9	180.3	161.3	179.4	131.6	133	119.9	122.6
Nortriptyline	7.7	15	<MQL	<MQL	<MQL	<MQL	<MQL	<MQL	<MQL	13.3	11.9	10.5	<MQL	2.5
Norsertraline	<MQL	<MQL	<MQL	<MQL	<MQL	<MQL	<MQL	<MQL	<MQL	<MQL	<MQL	157.8	<MQL	<MQL
Carbamazepine	442.9	439.6	640.2	653.3	580.6	604.8	685	646.7	547.8	540.4	426.2	419.3	334.7	335.8
Carbamazepine 10,11-epoxide	132.1	135.9	102.2	110.7	99.9	102.2	124.7	131.1	108	112.7	41.4	49.8	62.7	67.8
10,11-Dihydro -10-hydroxycarbamazepine	94.4	99.6	91.7	106.4	147.8	152.8	186.3	176.4	257.5	255	143	142.2	90.1	88.7
Diltiazem	278.3	264.2	250.4	261.9	261.5	283.4	308.2	289.5	287.2	269.2	237.4	215.5	119.6	120.1
Verapamil	<MQL	<MQL	<MQL	<MQL	<MQL	<MQL	<MQL	<MQL	<MQL	<MQL	<MQL	<MQL	<MQL	<MQL
Temazepam	2.7	1.2	2.4	5.3	12.3	13	25.6	16.8	21.9	17.3	10.6	4.1	<MQL	<MQL
Oxazepam	19.2	25.2	20.1	18.7	19.2	17.9	12.6	11.5	18.6	15.1	16.9	16.9	10.2	10.3
Diazepam	<MQL	<MQL	<MQL	<MQL	<MQL	<MQL	<MQL	<MQL	<MQL	<MQL	<MQL	<MQL	<MQL	<MQL
Quetiapine	132	132.8	50.8	59	36.9	40	58.6	55.8	56.2	54.6	76.7	71.7	27.8	29.4
Risperidone	1.2	0.8	<MQL	<MQL	<MQL	<MQL	<MQL	<MQL	<MQL	<MQL	<MQL	<MQL	<MQL	<MQL
Donepezil	<MQL	<MQL	<MQL	<MQL	<MQL	<MQL	<MQL	<MQL	<MQL	<MQL	<MQL	<MQL	<MQL	<MQL
Memantine	<MQL	<MQL	<MQL	<MQL	<MQL	<MQL	<MQL	<MQL	<MQL	<MQL	<MQL	<MQL	<MQL	<MQL
Creatinine	1340049.9	1495326.2	1413843.2	1576669.8	1573042.9	1656147.6	1628963.3	1641657.4	1439754.5	290325.8	255190.3	1435011.7	1432012.5	1597472.1
Nicotine	4177.1	3825.2	3496.1	3530.1	3938.7	4074.9	4233.9	4177.1	3496.1	3212.3	3155.5	3076.1	1884.2	2179.4
Caffeine	139854.1	138619.3	131782.7	126261.3	132545.7	131853	144020.3	140777.7	126482.1	125819.5	82621.5	82159.7	79398.9	78154.1
Cotinine	3561.1	3563.9	3345.8	3426.4	3401.1	3637.9	3601.3	3504.8	3227.8	3262	2507.2	2418.3	1471.8	1467.4
1,7-dimethylxanthine	195989	188964.1	168926.5	162623.2	179266.7	182908.8	174136	175229.8	164709.3	173064.8	94019.7	101800.2	98902.2	92328.3
Morphine	1588.3	1530.5	1423.7	1483.7	1465.9	1531.7	1651.5	1494.8	1601.6	1548.2	1062.2	1128.6	772	842.6
Dihydromorphine	100.6	118.3	149.7	177.3	156	146.9	200.8	163.2	110.8	131.8	131.2	129.8	72.5	82
Normorphine	198.3	242.8	241.6	230.4	179	226.1	192.2	251.6	209.5	194.2	128.7	139.1	102.8	71.5
Methadone	47.6	45.7	46.6	45.9	49.5	49.5	44.4	43.9	43.8	41.6	34.1	32.4	29.4	28.1
EDDP	75.9	75.5	68.5	75.9	70.4	72.2	78	79	63.7	63.6	53.4	50.7	39.9	40.8

	Day 1		Day 2		Day 3		Day 4		Day 5		Day 6		Day 7	
**Compound**	**A**	**B**	**A**	**B**	**A**	**B**	**A**	**B**	**A**	**B**	**A**	**B**	**A**	**B**
Codeine	2693.9	2721.7	2939.2	3023.7	3006.8	3053.9	2746.7	2934.8	2908.7	2961.6	2138.8	1991.6	1491.7	1372.6
Norcodeine	213.9	174.6	189.6	228.8	184.5	220.1	201.6	183.5	204.6	197.7	147.7	141.5	129.7	115.7
Dihydrocodeine	558.8	574.8	513.5	555.9	492.5	505.7	480	493.4	452.9	496.7	395.5	332.8	198.8	182
Tramadol	1642.3	1611.5	1434.4	1654.9	1360.6	1413.6	1409.1	1332	1222	1157	889.9	830.8	748.3	758.4
N-desmethyltramadol	1445.5	1423.6	1232.6	1357.8	1210.1	1257.4	1444.8	1322.1	1073.2	1092.2	720.6	679.8	403.1	408.2
O-desmethyltramadol	874.6	816.8	1002.8	890.6	766.1	796.6	1005.8	916.6	1030.2	1065	876.3	814.8	616.8	589.4
Amphetamine	557.9	534.7	475.5	502.1	517.6	593.8	561.4	511.1	631.3	669.5	349	379.3	202.2	217.3
Methamphetamine	20.4	19.8	9.3	11.1	11.2	11.4	14.4	15.3	10.6	11	18.7	17.8	11.7	11.6
MDMA	62.5	65.2	87.5	77.6	126.9	125.8	257.7	249.3	453.5	455	217.5	194.1	95.5	96.5
MDA	<MQL	<MQL	<MQL	<MQL	<MQL	<MQL	<MQL	<MQL	46.4	44.5	<MQL	<MQL	<MQL	<MQL
Cocaine	748.9	716.5	704	712.1	1067.3	1131.6	1165.9	1098.8	917.8	891.8	367.5	331.5	358.1	352.3
Benzoylecgonine	1931.7	1975.7	1799.7	1761	2424.9	2436.8	3466.5	3491.1	3169.2	2981.7	1205.8	1073.9	687.9	691.2
Anhydroecgoninemethylester	<MQL	<MQL	<MQL	<MQL	<MQL	<MQL	<MQL	<MQL	<MQL	<MQL	<MQL	<MQL	<MQL	<MQL
Cocaethylene	17.9	16.3	16.6	17.5	38	40.6	62	55	48.7	53.7	13.2	12.6	8.9	9.4
Mephedrone	<MQL	<MQL	<MQL	<MQL	<MQL	<MQL	<MQL	<MQL	<MQL	<MQL	<MQL	<MQL	<MQL	<MQL
MDPV	<MQL	<MQL	<MQL	<MQL	<MQL	<MQL	<MQL	<MQL	<MQL	<MQL	<MQL	<MQL	<MQL	<MQL
Heroin	<MQL	<MQL	<MQL	<MQL	<MQL	<MQL	<MQL	<MQL	<MQL	<MQL	<MQL	<MQL	<MQL	<MQL
6-acetylmorphine	<MQL	<MQL	<MQL	<MQL	<MQL	<MQL	<MQL	<MQL	<MQL	<MQL	<MQL	<MQL	<MQL	<MQL
Thiamethoxam	<MQL	<MQL	<MQL	<MQL	<MQL	<MQL	<MQL	<MQL	<MQL	<MQL	<MQL	<MQL	<MQL	<MQL
Imidacloprid	433.9	442	295.1	251.9	313.7	344.1	481.6	470.8	624.3	618.6	194.9	204.7	18.8	53.9
Clothianidin	<MQL	<MQL	<MQL	<MQL	<MQL	<MQL	<MQL	<MQL	<MQL	<MQL	<MQL	<MQL	<MQL	<MQL
Metazachlor	<MQL	<MQL	<MQL	<MQL	<MQL	<MQL	<MQL	<MQL	<MQL	<MQL	<MQL	<MQL	<MQL	<MQL
Terbuthylazine	<MQL	<MQL	<MQL	<MQL	<MQL	<MQL	<MQL	<MQL	<MQL	<MQL	<MQL	<MQL	<MQL	<MQL
Methiocarb	<MQL	<MQL	<MQL	<MQL	<MQL	<MQL	<MQL	3.1	<MQL	4.3	<MQL	<MQL	<MQL	<MQL
Dichlofluanid	29088.8	31767.8	30499.7	32272.8	32626.9	32962.3	25104.3	24874.5	<MQL	24288.3	25926.8	25299.9	21788.1	<MQL
Flufenacet	47.8	47.1	<MQL	<MQL	47.6	<MQL	<MQL	<MQL	49.2	49.1	80.9	81.7	60.5	58.9
Oxadiazon	<MQL	<MQL	<MQL	<MQL	<MQL	<MQL	<MQL	<MQL	<MQL	<MQL	<MQL	<MQL	<MQL	<MQL
Chlorpyrifos	N/A	N/A	N/A	N/A	N/A	N/A	N/A	N/A	N/A	N/A	N/A	N/A	N/A	N/A

Compound	Day 1		Day 2		Day 3		Day 4		Day 5		Day 6		Day 7	
	**A**	**B**	**A**	**B**	**A**	**B**	**A**	**B**	**A**	**B**	**A**	**B**	**A**	**B**
Triallate	<MQL	<MQL	<MQL	<MQL	<MQL	<MQL	<MQL	<MQL	<MQL	<MQL	<MQL	<MQL	<MQL	<MQL
Tylosin	<MQL	<MQL	<MQL	<MQL	<MQL	<MQL	<MQL	<MQL	<MQL	<MQL	<MQL	<MQL	<MQL	<MQL
Sulfapyridine	1740.3	1871.8	1382.8	1487.1	1695.3	1408.8	1212.3	1358.4	1496	1464.3	1006.5	1255.4	680.2	694.1
Sarafloxacin	<MQL	<MQL	<MQL	<MQL	<MQL	<MQL	<MQL	<MQL	<MQL	<MQL	<MQL	<MQL	<MQL	<MQL
Ceftiofur	256.9	208.2	309.7	398.4	484.5	471.8	582.5	616.9	285	621.7	661.9	524.2	<MQL	<MQL
Diazinon	17.7	18.2	17.4	17.3	<MQL	<MQL	17	17.1	17.2	19.3	17.3	17.7	17.2	16.9

Table S14 Solid particulate matter data (ng L^{-1})

Compound	Day 1		Day 2		Day 3		Day 4		Day 5		Day 6		Day 7	
	A	B	A	B	A	B	A	B	A	B	A	B	A	B
Benzophenone-1	11.9	13.3	8.8	8.5	47.7	36.2	12.4	16.9	8.4	11.1	3.8	4.4	5.5	4
Benzophenone-2	14.4	14.3	33.3	42.4	85.2	72.2	15.1	14.5	1.8	1.8	2.9	3.4	3.1	3.6
Benzophenone-3	N/A	N/A	N/A	N/A	N/A	N/A	N/A	N/A	N/A	N/A	N/A	N/A	N/A	N/A
Benzophenone-4	4.7	4.1	11.7	9.5	14.7	16.3	9	8.8	<MQL	<MQL	<MQL	<MQL	<MQL	<MQL
Methylparaben	39.3	43	315.9	345.3	138.7	267.6	84.1	99.4	75.3	62.2	37.7	27.3	76	97.4
Ethylparaben	10.4	8.7	<MQL	<MQL	6.1	91.1	16.7	31.2	<MQL	<MQL	2.5	1.4	<MQL	<MQL
Propylparaben	32	32.8	20.7	27.2	23.4	188.8	36.4	53.1	11.4	14.1	7	5.9	5.9	5.6
Butylparaben	6.6	9.6	2	2.1	16.2	11.6	137.4	262	79.1	18.3	5	4.1	5	5.6
Bisphenol A	3756.1	3209.9	1944.2	2312.4	2416.5	1949.7	264.1	229.7	1211.7	1647	106.7	106.6	101.7	118.8
E1	<MQL	<MQL	<MQL	<MQL	<MQL	<MQL	<MQL	<MQL	<MQL	<MQL	<MQL	<MQL	<MQL	<MQL
E2	<MQL	<MQL	<MQL	<MQL	<MQL	<MQL	<MQL	<MQL	<MQL	<MQL	<MQL	<MQL	<MQL	<MQL
EE2	N/A	N/A	N/A	N/A	N/A	N/A	N/A	N/A	N/A	N/A	N/A	N/A	N/A	N/A
Sulfasalazine	N/A	N/A	N/A	N/A	N/A	N/A	N/A	N/A	N/A	N/A	N/A	N/A	N/A	N/A
Clarithromycin	N/A	N/A	N/A	N/A	N/A	N/A	N/A	N/A	N/A	N/A	N/A	N/A	N/A	N/A
Azithromycin	<MQL	<MQL	<MQL	<MQL	<MQL	<MQL	<MQL	<MQL	<MQL	<MQL	<MQL	<MQL	<MQL	<MQL
Trimethoprim	17.4	16.3	20.5	19.5	20	18.1	40.9	16.9	10.4	12.5	10.5	10	10.9	9.9
Sulfamethoxazole	<MQL	<MQL	<MQL	<MQL	<MQL	<MQL	<MQL	<MQL	<MQL	<MQL	<MQL	<MQL	<MQL	<MQL
Triclosan	N/A	N/A	N/A	N/A	N/A	N/A	N/A	N/A	N/A	N/A	N/A	N/A	N/A	N/A
Amoxicillin	N/A	N/A	N/A	N/A	N/A	N/A	N/A	N/A	N/A	N/A	N/A	N/A	N/A	N/A
Metronidazole	<MQL	<MQL	<MQL	<MQL	<MQL	<MQL	<MQL	<MQL	<MQL	<MQL	<MQL	<MQL	<MQL	<MQL
Sulfadiazine	6	7.5	7.1	7.9	11.2	8.1	23.9	8.2	6.4	8.2	4.9	5.2	5.6	9.2
Cefalexin	N/A	N/A	N/A	N/A	N/A	N/A	N/A	N/A	N/A	N/A	N/A	N/A	N/A	N/A
Ofloxacin	N/A	N/A	N/A	N/A	N/A	N/A	N/A	N/A	N/A	N/A	N/A	N/A	N/A	N/A
Ciprofloxacin	N/A	N/A	N/A	N/A	N/A	N/A	N/A	N/A	N/A	N/A	N/A	N/A	N/A	N/A
Tetracycline	N/A	N/A	N/A	N/A	N/A	N/A	N/A	N/A	N/A	N/A	N/A	N/A	N/A	N/A
Danofloxacin	N/A	N/A	N/A	N/A	N/A	N/A	N/A	N/A	N/A	N/A	N/A	N/A	N/A	N/A
Oxytetracycline	N/A	N/A	N/A	N/A	N/A	N/A	N/A	N/A	N/A	N/A	N/A	N/A	N/A	N/A

	Day 1		Day 2		Day 3		Day 4		Day 5		Day 6		Day 7	
**Compound**	**A**	**B**	**A**	**B**	**A**	**B**	**A**	**B**	**A**	**B**	**A**	**B**	**A**	**B**
Chloramphenicol	<MQL	<MQL	<MQL	<MQL	<MQL	<MQL	<MQL	<MQL	<MQL	<MQL	<MQL	<MQL	<MQL	<MQL
Penicillin G	N/A	N/A	N/A	N/A	N/A	N/A	N/A	N/A	N/A	N/A	N/A	N/A	N/A	N/A
Penicillin V	<MQL	<MQL	<MQL	<MQL	<MQL	<MQL	<MQL	<MQL	<MQL	<MQL	<MQL	<MQL	<MQL	<MQL
Erythromycin	N/A	N/A	N/A	N/A	N/A	N/A	N/A	N/A	N/A	N/A	N/A	N/A	N/A	N/A
Prulifloxacin	N/A	N/A	N/A	N/A	N/A	N/A	N/A	N/A	N/A	N/A	N/A	N/A	N/A	N/A
Norfloxacin	N/A	N/A	N/A	N/A	N/A	N/A	N/A	N/A	N/A	N/A	N/A	N/A	N/A	N/A
Griseofulvin	<MQL	<MQL	<MQL	<MQL	<MQL	<MQL	<MQL	<MQL	<MQL	<MQL	<MQL	1.4	<MQL	<MQL
Ketoconazole	114.4	117	272.1	276.9	172.2	148	169.5	153.9	149.7	122.5	188.3	118.5	202.3	196.8
Valsartan	N/A	N/A	N/A	N/A	N/A	N/A	N/A	N/A	N/A	N/A	N/A	N/A	N/A	N/A
Irbesartan	N/A	N/A	N/A	N/A	N/A	N/A	N/A	N/A	N/A	N/A	N/A	N/A	N/A	N/A
Lisinopril	14.8	8.2	10.7	19	13.7	16.5	13.7	9.7	12.5	8.7	6.5	8.2	12.3	18.4
Ketoprofen	<MQL	<MQL	<MQL	<MQL	<MQL	<MQL	<MQL	<MQL	<MQL	<MQL	<MQL	<MQL	<MQL	<MQL
Ibuprofen	50.4	48	50.2	48.3	69	70.3	45.4	44.9	39.1	44.6	29.4	31.1	12.9	15.8
Naproxen	49	55	50	48.6	82.9	88.7	61.7	74.9	32.7	40.4	36	38.6	27.2	38.5
Diclofenac	<MQL	<MQL	<MQL	<MQL	<MQL	<MQL	<MQL	<MQL	<MQL	<MQL	<MQL	<MQL	<MQL	<MQL
Acetaminophen	29.2	27.9	9.7	8.4	50.6	37.2	13.9	22	13	13.8	56.6	99.4	5.3	5.9
Bezafibrate	2.7	2.9	4.6	2.9	4.2	4	4	4.4	1.4	1.8	2.5	2.5	0.9	1
Atorvastatin	N/A	N/A	N/A	N/A	N/A	N/A	N/A	N/A	N/A	N/A	N/A	N/A	N/A	N/A
Gemfibrozil	N/A	N/A	N/A	N/A	N/A	N/A	N/A	N/A	N/A	N/A	N/A	N/A	N/A	N/A
Candesartan Cilexetil	N/A	N/A	N/A	N/A	N/A	N/A	N/A	N/A	N/A	N/A	N/A	N/A	N/A	N/A
Fexofenadine	N/A	N/A	N/A	N/A	N/A	N/A	N/A	N/A	N/A	N/A	N/A	N/A	N/A	N/A
Cetirizine	N/A	N/A	N/A	N/A	N/A	N/A	N/A	N/A	N/A	N/A	N/A	N/A	N/A	N/A
Sildenafil	1	0.9	9.8	9.3	1.5	1.9	2.6	2.1	1.5	1.7	1	0.8	1.1	0.9
Metformin	N/A	N/A	N/A	N/A	N/A	N/A	N/A	N/A	N/A	N/A	N/A	N/A	N/A	N/A
Gliclazide	N/A	N/A	N/A	N/A	N/A	N/A	N/A	N/A	N/A	N/A	N/A	N/A	N/A	N/A
Sitagliptin	15.5	9.3	10.4	19.7	13.7	15.1	13.2	12.2	8.8	8.9	8.1	6.3	8.1	7.8
Pholcodine	<MQL	<MQL	<MQL	<MQL	<MQL	<MQL	<MQL	<MQL	<MQL	<MQL	<MQL	<MQL	<MQL	<MQL
Atenolol	21	11.8	17	14	20.6	25.9	62.4	29.6	17	25.6	11.7	29	25.4	15.6
Metoprolol	<MQL	<MQL	<MQL	<MQL	<MQL	<MQL	<MQL	<MQL	<MQL	<MQL	<MQL	<MQL	<MQL	<MQL

Compound	Day 1 A	Day 1 B	Day 2 A	Day 2 B	Day 3 A	Day 3 B	Day 4 A	Day 4 B	Day 5 A	Day 5 B	Day 6 A	Day 6 B	Day 7 A	Day 7 B
Propranolol	14.7	13.5	31.9	28.8	23.5	23.5	33.1	27.1	20.3	20.7	14.6	12.9	17.3	19.1
Bisoprolol	<MQL	<MQL	<MQL	<MQL	<MQL	<MQL	<MQL	<MQL	<MQL	<MQL	<MQL	<MQL	<MQL	<MQL
Ranitidine	<MQL	<MQL	<MQL	<MQL	<MQL	<MQL	<MQL	<MQL	<MQL	<MQL	<MQL	<MQL	<MQL	<MQL
Cimetidine	N/A	N/A	N/A	N/A	N/A	N/A	N/A	N/A	N/A	N/A	N/A	N/A	N/A	N/A
Iopromide	N/A	N/A	N/A	N/A	N/A	N/A	N/A	N/A	N/A	N/A	N/A	N/A	N/A	N/A
Buprenorphine	0.6	0.9	<MQL	0.8	<MQL	<MQL	<MQL	0.5	<MQL	<MQL	<MQL	<MQL	<MQL	0.3
Ephedrine/pseudoephedrine	6.7	5.4	4.5	5	6	6.6	5	4.6	2.8	2.7	3.3	3.5	4	2.7
Norephedrine	<MQL	<MQL	<MQL	<MQL	<MQL	<MQL	<MQL	<MQL	<MQL	<MQL	<MQL	<MQL	<MQL	<MQL
Azathioprine	N/A	N/A	N/A	N/A	N/A	N/A	N/A	N/A	N/A	N/A	N/A	N/A	N/A	N/A
Methotrexate	<MQL	<MQL	<MQL	<MQL	<MQL	<MQL	<MQL	<MQL	<MQL	<MQL	<MQL	<MQL	<MQL	<MQL
Ifosfamide	N/A	N/A	N/A	N/A	N/A	N/A	N/A	N/A	N/A	N/A	N/A	N/A	N/A	N/A
Tamoxifen	<MQL	<MQL	<MQL	<MQL	<MQL	<MQL	<MQL	<MQL	<MQL	<MQL	<MQL	<MQL	<MQL	<MQL
Imatinib	42.9	40.1	73.8	72.5	71.4	78.3	37.3	28	31.6	31.5	42.2	33.2	39.5	44.6
Capecitabine	<MQL	<MQL	<MQL	<MQL	<MQL	<MQL	<MQL	<MQL	<MQL	<MQL	<MQL	<MQL	<MQL	<MQL
Bicalutamide	9.4	10.7	8.4	8.6	9.1	<MQL	7.9	7.1	<MQL	<MQL	4	4.1	6.3	5.7
Ketamine	0.3	0.2	0.8	0.6	0.8	0.3	0.6	0.5	0.2	0.2	0.3	0.3	0.2	0.1
Norketamine	<MQL	<MQL	<MQL	<MQL	<MQL	<MQL	<MQL	<MQL	<MQL	<MQL	<MQL	<MQL	<MQL	<MQL
Venlafaxine	2.7	14.4	8	8.5	5.6	5.9	8.5	8.9	8.9	8.7	6.3	8.1	17.8	7.9
Desmethylvenlafaxine	2	1.4	2.4	2.7	2.4	2.3	4.1	3.7	5.5	4.3	2.6	2.7	2.2	1.8
Fluoxetine	170.7	69.8	46.9	48.8	31.4	28.1	27.8	30.2	27.6	28.5	21.8	21.7	29.2	29.1
Norfluoxetine	12.7	9.7	12.2	12.7	10.7	9.7	9.7	10.1	7	13.6	5.7	5.5	7.1	7.7
Sertraline	93.2	85.8	176.2	157.2	122	95.2	191.9	120.4	87.1	94.2	61.6	63.8	129.8	119.5
Mirtazapine	4.3	4.1	9.1	9.4	8.1	12.4	8.9	7.6	6.7	7.6	4	4	5.3	4.6
Citalopram	64.7	54.2	135.5	142.2	85.8	78.7	98.4	95.7	85.4	92	56.1	53.9	95.4	87.4
Desmethylcitalopram	21.1	18	45.9	47.6	30.4	27.8	35.1	39.2	33.3	34.7	20.9	20.5	37.3	32
Paroxetine	<MQL	<MQL	<MQL	<MQL	<MQL	<MQL	<MQL	<MQL	<MQL	<MQL	1.8	<MQL	<MQL	<MQL
Duloxetine	<MQL	<MQL	<MQL	<MQL	<MQL	<MQL	<MQL	<MQL	<MQL	<MQL	<MQL	<MQL	<MQL	<MQL
Amitriptyline	85.2	73.8	156.5	175.4	112.5	110.4	144.9	134.9	104.5	105.6	87.8	120.4	103.9	103.1

	Day 1		Day 2		Day 3		Day 4		Day 5		Day 6		Day 7	
**Compound**	**A**	**B**	**A**	**B**	**A**	**B**	**A**	**B**	**A**	**B**	**A**	**B**	**A**	**B**
Nortriptyline	6.2	6	15.9	16.7	10.9	10	10.4	8.7	8.1	8.8	6.9	9.3	10.9	10.1
Norsertraline	104.3	98.7	187	181.1	132.8	99.9	188.5	175.1	91.4	111.7	86	92.7	183.1	140.6
Carbamazepine	5.1	7.6	7	7.8	8.7	8.4	11.1	13.9	4.5	7.2	3.4	3.8	6.6	6.1
Carbamazepine 10,11-epoxide	N/A	N/A	N/A	N/A	N/A	N/A	N/A	N/A	N/A	N/A	N/A	N/A	N/A	N/A
10,11-Dihydro -10-hydroxycarbamazepine	<MQL	<MQL	<MQL	<MQL	<MQL	<MQL	<MQL	<MQL	<MQL	<MQL	<MQL	<MQL	<MQL	<MQL
Diltiazem	N/A	N/A	N/A	N/A	N/A	N/A	N/A	N/A	N/A	N/A	N/A	N/A	N/A	N/A
Verapamil	3	2.6	8.3	10.9	4.3	3.5	4.2	4.2	5.2	5	5.8	4.6	9.5	9.4
Temazepam	<MQL	<MQL	<MQL	<MQL	<MQL	<MQL	<MQL	<MQL	<MQL	<MQL	<MQL	<MQL	<MQL	<MQL
Oxazepam	N/A	N/A	N/A	N/A	N/A	N/A	N/A	N/A	N/A	N/A	N/A	N/A	N/A	N/A
Diazepam	0.7	<MQL	<MQL	<MQL	<MQL	<MQL	1.2	<MQL	<MQL	<MQL	<MQL	0.8	<MQL	<MQL
Quetiapine	4.9	4.5	3.3	3.1	1.2	1.4	2.6	3	7.1	3.8	1.6	1	1.6	1.2
Risperidone	0.1	0.2	0.1	0.1	0.1	0.1	0.1	0.1	0	<MQL	<MQL	<MQL	0.2	<MQL
Donepezil	<MQL	<MQL	<MQL	<MQL	<MQL	<MQL	0.6	<MQL	<MQL	<MQL	<MQL	<MQL	1.1	<MQL
Memantine	<MQL	<MQL	<MQL	<MQL	<MQL	<MQL	<MQL	<MQL	<MQL	<MQL	<MQL	<MQL	<MQL	<MQL
Creatinine	N/A	N/A	N/A	N/A	N/A	N/A	N/A	N/A	N/A	N/A	N/A	N/A	N/A	N/A
Nicotine	29.3	31.9	29.7	30.1	80.1	59.5	122	80.8	44.1	63	35.1	46.2	17.4	21.7
Caffeine	N/A	N/A	N/A	N/A	N/A	N/A	N/A	N/A	N/A	N/A	N/A	N/A	N/A	N/A
Cotinine	7.8	7.6	14.1	11.7	14.8	14	12.5	11.4	8.1	9.6	8.1	7.6	5.3	6.3
1,7-dimethylxanthine	N/A	N/A	N/A	N/A	N/A	N/A	N/A	N/A	N/A	N/A	N/A	N/A	N/A	N/A
Morphine	5.8	6.4	11.1	13.6	7.7	27.8	11.8	8.8	7.1	11.7	5.7	6	7.8	10.5
Dihydromorphine	<MQL	<MQL	<MQL	<MQL	<MQL	<MQL	<MQL	<MQL	<MQL	<MQL	<MQL	<MQL	<MQL	<MQL
Normorphine	<MQL	<MQL	<MQL	<MQL	<MQL	<MQL	<MQL	<MQL	<MQL	<MQL	<MQL	<MQL	<MQL	<MQL
Methadone	1.4	1	2.1	2.2	2	2.5	2.2	2.1	1.3	1.4	0.9	0.9	1	1.1
EDDP	5	5	13.6	14.4	10.3	16.2	19.3	22	9	11	4.9	5.2	9.4	8.4
Codeine	19	17	28	27.7	28.1	27.2	34.8	29.4	22.9	25.7	17.2	15.6	16.3	16.7
Norcodeine	<MQL	<MQL	<MQL	<MQL	<MQL	<MQL	<MQL	<MQL	<MQL	<MQL	<MQL	<MQL	<MQL	<MQL
Dihydrocodeine	2.6	2	4.6	3.6	3.8	3.6	5.1	4.5	3.6	4.9	2.4	2.6	2.4	2.4

	Day 1		Day 2		Day 3		Day 4		Day 5		Day 6		Day 7	
**Compound**	**A**	**B**	**A**	**B**	**A**	**B**	**A**	**B**	**A**	**B**	**A**	**B**	**A**	**B**
Tramadol	3.8	3	4.6	4.2	4.3	4.6	5.9	5.2	3.1	3.4	2.8	2.8	2.6	2.2
N-desmethyltramadol	1.3	1	2.3	2.1	1.8	1.5	2.3	2	1.2	1.5	1.1	1	0.9	0.9
O-desmethyltramadol	N/A	N/A	N/A	N/A	N/A	N/A	N/A	N/A	N/A	N/A	N/A	N/A	N/A	N/A
Amphetamine	<MQL	<MQL	<MQL	<MQL	<MQL	<MQL	<MQL	<MQL	<MQL	<MQL	<MQL	<MQL	<MQL	<MQL
Methamphetamine	<MQL	<MQL	<MQL	<MQL	<MQL	<MQL	<MQL	<MQL	<MQL	<MQL	<MQL	<MQL	<MQL	<MQL
MDMA	0.4	0.3	0.8	0.8	1	0.9	1.9	1.8	1.3	5.3	1.8	1.6	0.9	0.9
MDA	N/A	N/A	N/A	N/A	N/A	N/A	N/A	N/A	N/A	N/A	N/A	N/A	N/A	N/A
Cocaine	5.3	5	6.3	6.1	10.1	10.1	13.9	12.7	4.4	5	5.1	4.8	4.3	3.9
Benzoylecgonine	1.5	1.4	1.1	1.1	2.9	2.7	3.9	3.7	0.9	1	2.1	2.4	0.7	0.5
Anhydroecgoninemethylester	N/A	N/A	N/A	N/A	N/A	N/A	N/A	N/A	N/A	N/A	N/A	N/A	N/A	N/A
Cocaethylene	<MQL	<MQL	<MQL	<MQL	0.2	0.2	0.8	0.9	0.1	0.4	0.4	0.3	<MQL	<MQL
Mephedrone	<MQL	<MQL	<MQL	<MQL	<MQL	<MQL	<MQL	<MQL	<MQL	<MQL	<MQL	<MQL	<MQL	<MQL
MDPV	<MQL	<MQL	<MQL	<MQL	<MQL	<MQL	<MQL	<MQL	<MQL	<MQL	<MQL	<MQL	<MQL	<MQL
Heroin	<MQL	<MQL	<MQL	<MQL	<MQL	<MQL	<MQL	<MQL	<MQL	<MQL	<MQL	<MQL	<MQL	<MQL
6-acetylmorphine	N/A	N/A	N/A	N/A	N/A	N/A	N/A	N/A	N/A	N/A	N/A	N/A	N/A	N/A
Thiamethoxam	<MQL	<MQL	<MQL	<MQL	<MQL	<MQL	<MQL	<MQL	<MQL	<MQL	<MQL	<MQL	<MQL	<MQL
Imidacloprid	<MQL	<MQL	<MQL	<MQL	<MQL	<MQL	<MQL	<MQL	<MQL	<MQL	<MQL	<MQL	<MQL	<MQL
Clothianidin	<MQL	<MQL	0.8	4.6	<MQL	<MQL	<MQL	<MQL	1.3	0.2	<MQL	<MQL	2.6	1.7
Metazachlor	<MQL	1.3	10.7	<MQL	<MQL	4.5	<MQL	<MQL	<MQL	0.1	<MQL	<MQL	<MQL	<MQL
Terbuthylazine	<MQL	<MQL	<MQL	<MQL	<MQL	<MQL	<MQL	<MQL	<MQL	<MQL	<MQL	<MQL	<MQL	<MQL
Methiocarb	0.3	0.2	0.9	0.9	0.6	1.4	0.2	0.5	3.2	0.8	<MQL	0.1	0.9	1
Dichlofluanid	N/A	N/A	N/A	N/A	N/A	N/A	N/A	N/A	N/A	N/A	N/A	N/A	N/A	N/A
Flufenacet	4.1	4.1	4.5	<MQL	5	5.2	<MQL	<MQL	4.1	3.6	<MQL	<MQL	2.9	2.9
Oxadiazon	<MQL	<MQL	<MQL	<MQL	<MQL	<MQL	9.7	<MQL	<MQL	<MQL	<MQL	<MQL	<MQL	8.9
Chlorpyrifos	N/A	N/A	N/A	N/A	N/A	N/A	N/A	N/A	N/A	N/A	N/A	N/A	N/A	N/A
Triallate	N/A	N/A	N/A	N/A	N/A	N/A	N/A	N/A	N/A	N/A	N/A	N/A	N/A	N/A
Tylosin	N/A	N/A	N/A	N/A	N/A	N/A	N/A	N/A	N/A	N/A	N/A	N/A	N/A	N/A
Sulfapyridine	N/A	N/A	N/A	N/A	N/A	N/A	N/A	N/A	N/A	N/A	N/A	N/A	N/A	N/A

Compound	Day 1 A	Day 1 B	Day 2 A	Day 2 B	Day 3 A	Day 3 B	Day 4 A	Day 4 B	Day 5 A	Day 5 B	Day 6 A	Day 6 B	Day 7 A	Day 7 B
Sarafloxacin	N/A	N/A	N/A	N/A	N/A	N/A	N/A	N/A	N/A	N/A	N/A	N/A	N/A	N/A
Ceftiofur	N/A	N/A	N/A	N/A	N/A	N/A	N/A	N/A	N/A	N/A	N/A	N/A	N/A	N/A
Diazinon	4.5	14.8	<MQL	17.9	<MQL	<MQL	<MQL	<MQL	<MQL	7.5	12.6	<MQL	<MQL	<MQL

Table S15 Digested solids data (ng g^{-1})

	Day 1		Day 2		Day 3	
**Compound**	**A**	**B**	**A**	**B**	**A**	**B**
Benzophenone-1	N/A	N/A	N/A	N/A	N/A	N/A
Benzophenone-2	21.3	10.9	7.9	8.9	5.2	7.0
Benzophenone-3	N/A	N/A	N/A	N/A	N/A	N/A
Benzophenone-4	<MQL	<MQL	<MQL	<MQL	<MQL	<MQL
Methylparaben	617.3	431.8	331.8	290.7	215.2	274.3
Ethylparaben	<MQL	<MQL	<MQL	<MQL	<MQL	<MQL
Propylparaben	<MQL	<MQL	<MQL	<MQL	<MQL	<MQL
Butylparaben	<MQL	<MQL	<MQL	<MQL	<MQL	<MQL
Bisphenol A	4984.4	4479.9	4220.1	4200.5	4010.5	4298.3
E1	40.5	47.0	39.0	40.0	43.8	38.9
E2	<MQL	<MQL	<MQL	<MQL	<MQL	<MQL
EE2	N/A	N/A	N/A	N/A	N/A	N/A
Sulfasalazine	N/A	N/A	N/A	N/A	N/A	N/A
Clarithromycin	N/A	N/A	N/A	N/A	N/A	N/A
Azithromycin	1.7	16.6	3.1	3.6	8.6	0.3
Trimethoprim	14.4	15.1	8.5	7.9	10.3	10.2
Sulfamethoxazole	<MQL	<MQL	<MQL	<MQL	<MQL	<MQL
Triclosan	N/A	N/A	N/A	N/A	N/A	N/A
Amoxicillin	N/A	N/A	N/A	N/A	N/A	N/A
Metronidazole	<MQL	<MQL	<MQL	<MQL	<MQL	<MQL
Sulfadiazine	<MQL	<MQL	<MQL	<MQL	<MQL	<MQL
Cefalexin	N/A	N/A	N/A	N/A	N/A	N/A
Ofloxacin	N/A	N/A	N/A	N/A	N/A	N/A
Ciprofloxacin	N/A	N/A	N/A	N/A	N/A	N/A
Tetracycline	N/A	N/A	N/A	N/A	N/A	N/A
Danofloxacin	<MQL	<MQL	<MQL	<MQL	<MQL	<MQL
Oxytetracycline	N/A	N/A	N/A	N/A	N/A	N/A
Chloramphenicol	<MQL	<MQL	359.4	368.8	328.7	453.6
Penicillin G	N/A	N/A	N/A	N/A	N/A	N/A
Penicillin V	N/A	N/A	N/A	N/A	N/A	N/A
Erythromycin	N/A	N/A	N/A	N/A	N/A	N/A
Prulifloxacin	N/A	N/A	N/A	N/A	N/A	N/A
Norfloxacin	N/A	N/A	N/A	N/A	N/A	N/A
Griseofulvin	<MQL	<MQL	<MQL	<MQL	<MQL	<MQL
Ketoconazole	1164.2	1234.1	602.8	860.5	867.4	847.0
Valsartan	N/A	N/A	N/A	N/A	N/A	N/A
Irbesartan	N/A	N/A	N/A	N/A	N/A	N/A
Lisinopril	<MQL	<MQL	<MQL	<MQL	<MQL	<MQL
Ketoprofen	<MQL	<MQL	<MQL	<MQL	<MQL	<MQL
Ibuprofen	298.3	266.0	178.4	230.9	213.5	203.8
Naproxen	114.1	110.0	100.6	111.9	101.4	112.2
Diclofenac	33.1	27.7	23.5	24.6	23.1	25.2
Acetaminophen	<MQL	<MQL	<MQL	<MQL	<MQL	<MQL
Bezafibrate	7.8	8.8	7.5	7.2	7.2	6.4

Compound	Day 1		Day 2		Day 3	
	A	B	A	B	A	B
Atorvastatin	N/A	N/A	N/A	N/A	N/A	N/A
Gemfibrozil	1115.2	866.8	814.3	1025.1	709.4	566.6
Candesartan Cilexetil	N/A	N/A	N/A	N/A	N/A	N/A
Fexofenadine	N/A	N/A	N/A	N/A	N/A	N/A
Cetirizine	N/A	N/A	N/A	N/A	N/A	N/A
Sildenafil	20.8	19.5	22.6	21.3	20.6	20.2
Metformin	N/A	N/A	N/A	N/A	N/A	N/A
Gliclazide	N/A	N/A	N/A	N/A	N/A	N/A
Sitagliptin	29.3	26.9	30.3	29.1	25.9	24.2
Pholcodine	<MQL	<MQL	<MQL	<MQL	<MQL	<MQL
Atenolol	<MQL	<MQL	<MQL	<MQL	<MQL	<MQL
Metoprolol	<MQL	<MQL	<MQL	<MQL	<MQL	<MQL
Propranolol	236.3	223.6	189.7	183.4	176.0	182.0
Bisoprolol	<MQL	<MQL	<MQL	<MQL	<MQL	<MQL
Ranitidine	<MQL	<MQL	<MQL	<MQL	<MQL	<MQL
Cimetidine	N/A	N/A	N/A	N/A	N/A	N/A
Iopromide	N/A	N/A	N/A	N/A	N/A	N/A
Buprenorphine	18.7	4.6	13.8	12.1	13.2	16.9
Ephedrine/pseudoephedrine	<MQL	<MQL	<MQL	<MQL	<MQL	<MQL
Norephedrine	<MQL	<MQL	<MQL	<MQL	<MQL	<MQL
Azathioprine	N/A	N/A	N/A	N/A	N/A	N/A
Methotrexate	<MQL	<MQL	<MQL	<MQL	<MQL	<MQL
Ifosfamide	N/A	N/A	N/A	N/A	N/A	N/A
Tamoxifen	<MQL	<MQL	<MQL	<MQL	<MQL	<MQL
Imatinib	164.9	188.9	94.8	113.5	75.5	100.2
Capecitabine	<MQL	<MQL	<MQL	<MQL	<MQL	<MQL
Bicalutamide	71.5	54.1	40.4	53.7	44.5	36.1
Ketamine	3.6	3.4	2.3	2.5	2.8	2.1
Norketamine	1.1	1.2	0.6	0.7	0.7	0.7
Venlafaxine	134.7	180.1	108.5	108.8	105.8	120.0
Desmethylvenlafaxine	25.2	23.6	23.2	21.5	20.0	20.9
Fluoxetine	238.8	242.3	168.2	172.7	169.1	171.3
Norfluoxetine	133.7	123.1	77.2	73.8	68.0	72.4
Sertraline	677.1	653.4	527.3	514.2	506.6	512.8
Mirtazapine	80.5	78.0	60.7	60.6	60.4	74.3
Citalopram	897.2	879.1	746.2	723.6	737.7	714.0
Desmethylcitalopram	392.4	374.1	263.1	257.7	246.9	240.1
Paroxetine	5.4	6.8	0.4	0.4	<MQL	<MQL
Duloxetine	25.9	25.0	16.6	13.8	11.1	12.8
Amitriptyline	546.3	513.1	453.9	458.2	415.5	444.3
Nortriptyline	86.7	82.2	55.8	52.8	48.9	53.0
Norsertraline	N/A	N/A	N/A	N/A	N/A	N/A
Carbamazepine	137.2	132.2	113.8	110.4	109.1	109.3
Carbamazepine 10,11-epoxide	N/A	N/A	N/A	N/A	N/A	N/A

Compound	Day 1		Day 2		Day 3	
	A	B	A	B	A	B
10,11-Dihydro -10-hydroxycarbamazepine	<MQL	<MQL	<MQL	<MQL	<MQL	<MQL
Diltiazem	N/A	N/A	N/A	N/A	N/A	N/A
Verapamil	60.6	58.5	46.8	45.8	46.2	48.6
Temazepam	<MQL	<MQL	<MQL	<MQL	<MQL	<MQL
Oxazepam	<MQL	<MQL	<MQL	<MQL	<MQL	<MQL
Diazepam	4.9	4.0	3.9	4.5	3.9	4.3
Quetiapine	18.4	18.0	13.9	13.9	13.1	26.7
Risperidone	<MQL	<MQL	<MQL	<MQL	<MQL	<MQL
Memantine	<MQL	<MQL	<MQL	<MQL	<MQL	<MQL
Donepezil	8.4	8.1	<MQL	<MQL	<MQL	<MQL
Creatinine	N/A	N/A	N/A	N/A	N/A	N/A
Nicotine	305.7	262.8	208.3	203.6	191.6	190.8
Caffeine	N/A	N/A	N/A	N/A	N/A	N/A
Cotinine	49.6	43.0	39.3	37.1	36.7	35.0
1,7-dimethylxanthine	N/A	N/A	N/A	N/A	N/A	N/A
Morphine	104.5	108.5	<MQL	<MQL	22.6	17.2
Dihydromorphine	<MQL	<MQL	<MQL	<MQL	<MQL	<MQL
Normorphine	<MQL	<MQL	<MQL	<MQL	<MQL	<MQL
Methadone	16.4	14.3	12.2	11.7	11.5	11.4
EDDP	75.8	62.4	25.5	24.2	17.3	24.4
Codeine	108.4	101.5	12.6	12.8	19.0	15.8
Norcodeine	<MQL	<MQL	<MQL	<MQL	<MQL	<MQL
Dihydrocodeine	30.8	29.2	23.0	22.1	22.6	21.0
Tramadol	50.1	37.7	31.6	32.2	29.3	26.4
N-desmethyltramadol	27.3	21.8	13.8	13.4	13.7	12.4
O-desmethyltramadol	N/A	N/A	N/A	N/A	N/A	N/A
Amphetamine	<MQL	<MQL	<MQL	<MQL	<MQL	<MQL
Methamphetamine	<MQL	<MQL	<MQL	<MQL	<MQL	<MQL
MDMA	7.8	7.1	5.0	5.7	5.6	5.4
MDA	N/A	N/A	N/A	N/A	N/A	N/A
Cocaine	<MQL	<MQL	<MQL	<MQL	<MQL	<MQL
Benzoylecgonine	<MQL	<MQL	<MQL	<MQL	<MQL	<MQL
Anhydroecgoninemethylester	N/A	N/A	N/A	N/A	N/A	N/A
Cocaethylene	<MQL	<MQL	<MQL	<MQL	<MQL	<MQL
Mephedrone	<MQL	<MQL	<MQL	<MQL	<MQL	<MQL
MDPV	<MQL	<MQL	<MQL	<MQL	<MQL	<MQL
Heroin	<MQL	<MQL	<MQL	<MQL	<MQL	<MQL
6-acetylmorphine	N/A	N/A	N/A	N/A	N/A	N/A
Thiamethoxam	<MQL	<MQL	<MQL	<MQL	<MQL	<MQL
Imidacloprid	<MQL	<MQL	<MQL	<MQL	<MQL	<MQL
Clothianidin	<MQL	<MQL	<MQL	<MQL	<MQL	<MQL
Metazachlor	<MQL	<MQL	<MQL	<MQL	<MQL	<MQL
Terbuthylazine	<MQL	<MQL	<MQL	<MQL	<MQL	<MQL
Methiocarb	<MQL	1.7	1.6	<MQL	<MQL	<MQL

Compound	Day 1 A	Day 1 B	Day 2 A	Day 2 B	Day 3 A	Day 3 B
Dichlofluanid	N/A	N/A	N/A	N/A	N/A	N/A
Flufenacet	<MQL	15.4	14.7	<MQL	14.0	14.7
Oxadiazon	<MQL	<MQL	<MQL	<MQL	<MQL	<MQL
Chlorpyrifos	<MQL	<MQL	93.8	<MQL	<MQL	<MQL
Triallate	<MQL	<MQL	<MQL	<MQL	<MQL	<MQL
Tylosin	N/A	N/A	N/A	N/A	N/A	N/A
Sulfapyridine	N/A	N/A	N/A	N/A	N/A	N/A
Sarafloxacin	<MQL	<MQL	<MQL	<MQL	<MQL	<MQL
Ceftiofur	<MQL	<MQL	<MQL	<MQL	<MQL	<MQL
Diazinon	17.4	<MQL	<MQL	10.8	<MQL	<MQL
